# An intracellular membrane protein GEP1 regulates xanthurenic acid induced gametogenesis of malaria parasites

**DOI:** 10.1038/s41467-020-15479-3

**Published:** 2020-04-09

**Authors:** Yuanyuan Jiang, Jun Wei, Huiting Cui, Chuanyuan Liu, Yuan Zhi, ZhengZheng Jiang, Zhenkui Li, Shaoneng Li, Zhenke Yang, Xu Wang, Pengge Qian, Cui Zhang, Chuanqi Zhong, Xin-zhuan Su, Jing Yuan

**Affiliations:** 1 State Key Laboratory of Cellular Stress Biology, Innovation Center for Cell Signaling Network, School of Life Sciences, Xiamen University, 361102 Xiamen, Fujian China; 2 Lingnan Guangdong Laboratory of Modern Agriculture, 510642 Guangzhou, China; 3 Laboratory of Malaria and Vector Research, National Institute of Allergy and Infectious Diseases, National Institutes of Health, Bethesda, MD 20892 USA

**Keywords:** Parasite biology, Parasite development, Parasite host response, Malaria

## Abstract

Gametocytes differentiation to gametes (gametogenesis) within mosquitos is essential for malaria parasite transmission. Both reduction in temperature and mosquito-derived XA or elevated pH are required for triggering cGMP/PKG dependent gametogenesis. However, the parasite molecule for sensing or transducing these environmental signals to initiate gametogenesis remains unknown. Here we perform a CRISPR/Cas9-based functional screening of 59 membrane proteins expressed in the gametocytes of *Plasmodium yoelii* and identify that GEP1 is required for XA-stimulated gametogenesis. GEP1 disruption abolishes XA-stimulated cGMP synthesis and the subsequent signaling and cellular events, such as Ca^2+^ mobilization, gamete formation, and gametes egress out of erythrocytes. GEP1 interacts with GCα, a cGMP synthesizing enzyme in gametocytes. Both GEP1 and GCα are expressed in cytoplasmic puncta of both male and female gametocytes. Depletion of GCα impairs XA-stimulated gametogenesis, mimicking the defect of GEP1 disruption. The identification of GEP1 being essential for gametogenesis provides a potential new target for intervention of parasite transmission.

## Abstract (web summary)

Mosquito-derived xanthurenic acid (XA) is a trigger for gametogenesis of *Plasmodium* parasites. Here the authors show that the putative amino acid transporter GEP1 is required for XA-stimulated gametogenesis in *Plasmodium yoelii* and that it interacts with guanylyl cyclase α (GCα), a cGMP synthesizing enzyme in gametocytes.

## Introduction

Male and female gametocytes are sexual precursor cells essential for malaria parasite transmission. Within 10–15 min after being taken up by a mosquito, gametocytes differentiate into gametes in mosquito midgut, a process known as gametogenesis. A female gametocyte forms a rounded female gamete, whereas a male gametocyte undergoes three mitotic divisions, assembles eight intracytoplasmic axonemes, and produces eight flagellated male gametes^1^. Both male and female gametes egress from their residing erythrocytes via an inside-out mechanism, during which the parasitophorus vacuole membrane (PVM) ruptures prior to the opening of the erythrocyte membrane (EM)^2^. After the release from erythrocytes, the male and female gametes fertilize to produce zygotes and then the motile ookinetes that penetrate mosquito midgut wall to develop into oocysts each containing thousands of sporozoites. The sporozoites then migrate to mosquito salivary glands and are injected into a new host when the mosquito bites again.

Gametogenesis is triggered by two stimuli, a drop in temperature of approximately 5 °C^3,4^ and the presence of xanthurenic acid (XA) that is a metabolite of tryptophan from mosquito^5,6^. An additional signal reported to induce gametogenesis is an increase in pH from 7.4 to 8^4^. Since the groundbreaking discovery of XA as a trigger for *Plasmodium* gametogenesis in mosquitoes, studies have shown that XA can enhance parasite guanylyl cyclase (GC) activity on gametocyte membrane fraction, leading to increased level of second messenger 3’−5’-cyclic guanosine monophosphate (cGMP)^7^. Two integral membrane GC proteins (GCα and GCβ) are found in *Plasmodium* parasites. GCα has been implicated to be responsible for cGMP synthesis during gametogenesis because disruption of GCβ has no effect on XA-induced gametogenesis^8–10^. The increased level of cGMP activates cGMP-dependent protein kinase G (PKG) that functions as a master regulator of the downstream signaling events during gametogenesis^11^. Inhibition of PKG using Compound 2 (C2) prevented gametocytes rounding up, gamete formation of both sexes, and gametes egress from erythrocytes in *P. falciparum* and *P. berghei*^11,12^. PKG-dependent Ca^2+^ mobilization was also observed in the cytosol of *P. falciparum* and *P. berghei* gametocytes 10–15 s after addition of XA^13,14^. PKG activates the synthesis of inositol (1,4,5)-trisphosphate (IP3) via phosphoinositide metabolism and triggers cytosolic mobilization of Ca^2+^ that likely originates from the endoplasmic reticulum^15^. Unfortunately, the molecule(s) responsible for sensing XA or transducing the XA-stimulated signal to activate the cGMP-PKG signaling remain unknown.

Membrane proteins are known to play critical roles in sensing, transporting, and/or transducing environmental signals to initiate cellular responses. To identify potential molecules involved in sensing or transducing XA signal during gametogenesis, we perform CRISPR/Cas9-mediated genetic deletion screens of 59 candidate genes encoding integral membrane proteins expressed in gametocytes of the rodent malaria parasite *P. yoelii*. We identify a multiple-spanning membrane protein GEP1 (gametogenesis essential protein 1) that was essential for XA-stimulated gametogenesis. Disruption of GEP1 completely abolishes XA-stimulated gametogenesis of both sexes. Parasites deficient of GEP1 show no synthesis of XA-stimulated cGMP and no downstream cellular and signaling events such as Ca^2+^ mobilization, parasite egress out of PVM and EM, genome replication and axoneme assembly in male gametocytes, and release of translational repression in female gametocytes. GEP1 interacts with GCα in gametocytes, and GCα depletion also impairs XA-stimulated gametogenesis, mimicking the effects of GEP1 disruption. This study identifies a molecule essential for the initiation of gametogenesis and a potential target for blocking parasite transmission.

## Results

### GEP1 is essential for XA-stimulated gametogenesis

To identify membrane proteins critical in sensing XA or transducing XA-induced signal during gametogenesis, we identified 59 *P. yoelii* genes that are expressed in gametocytes and encode proteins with 1 to 22 predicted transmembrane domains (TMs) from the PlasmoDB database (Supplementary Table 1). We designed single guide RNA (sgRNA) to disrupt each of these genes using CRISPR/Cas9 methods^16,17^ and were able to successfully knockout (KO) 45 (76%) of the genes in the *P. yoelii* 17XNL strain, obtaining at least two cloned lines for each mutant (Supplementary Fig. 1a, c, d, i). The remaining 14 genes (24%) were refractory to repeated deletion attempts using three independent sgRNA sequences, suggesting their essential roles for asexual blood-stage growth.

**Fig. 1:**
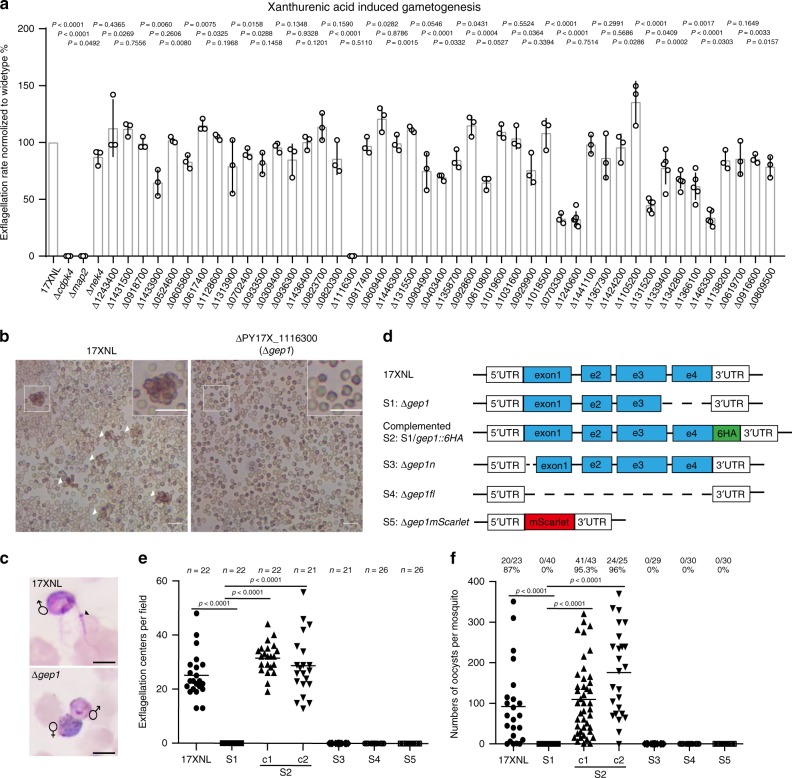
Membrane proteins screening identified *gep1* essential for gametogenesis. **a** In vitro XA stimulated exflagellation rates for *P. yoelii* 17XNL wild type (WT) and 45 mutant strains each with a specific gene disruption. The exflagellation rate of each mutant was normalized with that of WT parallelly tested each time. The numbers for the gene name are the gene IDs derived in PlasmoDB. Data are shown as mean ± SD from *n* = 3 independent experiments for strains except *n* = 5 for ∆*1315200*, ∆*1339400*, ∆*1342800*, ∆*1366100* and ∆*1463300*, and *n* = 6 for ∆*1240600*. **b** Representative images of XA stimulated exflagellation centers (ECs, white arrows) under light microscope (10×). Scale bar = 20 μm. **c** Images of the exflagellated male gametes (Black arrow) after Giemsa staining under light microscope (100×). Scale bar = 5 μm. **d** Diagrams of WT *gep1* gene structure and various mutants: S1 (∆*gep1*), deletion in C-terminus; S2 (∆*gep1/gep1::6HA*), reconstructed *gep1* with a 6HA tag; S3 (∆*gep1n*), deletion in N-terminus; S4 (∆*gep1fl*), deletion of the full coding region; S5 (∆*gep1mScarlet*), coding region replaced with *mScarlet* gene. **e** XA-stimulated EC counts from WT and the *gep1* mutants. c1 and c2 are two clones of S2 parasite. *n* is the numbers of microscopic fields counted (40×). **f** Oocyst counts from WT and the *gep1* mutants. Oocysts are counted from the mosquito midguts 7 days post blood feeding. *x*/*y* on the top is the number of mosquito containing oocyst/the number of mosquito dissected; the percentage number is the mosquito infection prevalence. Experiments were independently repeated six times in **b**, and three times in **c**, **e**, and **f**. Two-tailed unpaired Student’s *t* test was applied in **a**, **e**, and **f**. Source data of **a**, **e**, and **f** are provided as a Source Data file.

The 45 gene deletion mutants proliferated asexually in mouse blood normally and were able to produce both male and female gametocytes although the gametocytemia level varied among these mutants (Supplementary Fig. 2, Supplementary Fig. 3a). Next we measured the gametogenesis of male gametocyte by counting exflagellation centers (ECs) formed in vitro after stimulation with 50 μM XA at 22 °C. Only one mutant (PY17X_1116300 disruption) showed complete deficiency in EC formation and male gamete release (Fig. 1a–c). The PY17X_1116300 gene contains four exons (Fig. 1d) encoding a putative amino acid transporter protein that is essential for gametogenesis; we therefore name the gene *gep1* for gametogenesis essential protein 1. As controls, disruption of *P. yoelii cdpk4* or *map2* also caused defect in EC formation (Fig. 1a), confirming the phenotypes observed in *P. berghei*^13,18^. Consequently, the ∆*gep1* mutant parasite produced no ookinete in in vitro culture (Supplementary Fig. 3b), oocyst in *Anopheles stephensi* midgut (Fig. 1f), or sporozoite in mosquito salivary gland (Supplementary Fig. 3c).

To further confirm the phenotype of ∆*gep1*, we generated three additional *gep1* mutant parasites (∆*gep1n*, ∆*gep1fl*, and ∆*gep1mScarlet*) (Fig. 1d, Supplementary Fig. 1c–e). The ∆*gep1n* parasite had a 464 bp deletion at the 5’ coding region, causing a frameshift for the remaining coding region. The ∆*gep1fl* parasite had the whole *gep1* coding region deleted, and the ∆*gep1mScarlet* parasite had its *gep1* coding regions replaced with a gene encoding red florescent protein mScarlet. These mutations were confirmed by PCR and DNA sequencing (Supplementary Fig. 1j, k), and the mutant parasites displayed developmental phenotypes similar to those of ∆*gep1* in both mouse and mosquito stages (Fig. 1e, f, Supplementary Fig. 3a–c). We also reintroduced the 558 bp deleted segment plus a sextuple HA epitope (6HA) into the ∆*gep1* parasite to rescue the gene function using Cas9-mediated homologous replacement (Fig. 1d, Supplementary Fig. 1b, j). Two clones of the rescued parasite (∆*gep1*/*gep1::6HA*c1 and ∆*gep1*/*gep1::6HA*c2) showed expression of the GEP1::6HA protein in both Western blotting and immunofluorescence analysis (IFA) (Supplementary Fig. 3d, e). Importantly, both clones produced wild type (WT) levels of EC in vitro (Fig. 1e) and midgut oocyst in mosquitoes (Fig. 1f). The GEP1 protein is well-conserved among *P. yoelii, P. berghei*, and the human *P. falciparum* parasites (Supplementary Fig. 4), suggesting conserved function. Deletion of *P. berghei gep1* gene (PBANKA_1115100) resulted in parasite clones that failed to form XA-stimulated ECs in vitro and midgut oocyst in mosquitoes (Supplementary Fig. 1l, m, Supplementary Fig. 3f–h). Together, these results demonstrate that GEP1 depletion completely block male gametogenesis and mosquito transmission of malaria parasites.

### GEP1 is expressed in cytosol puncta of gametocytes

**Fig. 2:**
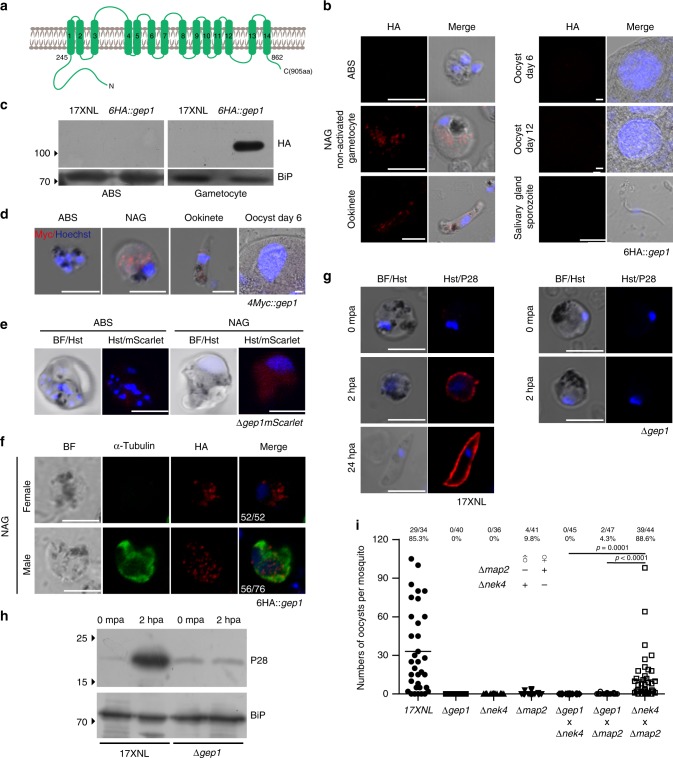
GEP1 is essential for gametogenesis of both sexes. **a** Predicted GEP1 protein structure with 14 TM domains (green bar) and cytoplasmic N-termini and C-termini. **b** IFA analysis of GEP1 expression in asexual blood stages (ABS), gametocytes, ookinetes, oocysts, and sporozoites of the *6HA::gep1* parasite using anti-HA antibody. Hoechst 33342 (Blue) is used for nuclear acid stain for all images in this figure. **c** Western blot analysis of GEP1 in ABS and gametocytes of the *6HA::gep1* parasite. BiP as loading control. **d** IFA analysis of GEP1 in the *4Myc::gep1* parasite using anti-Myc antibody. **e** mScarlet fluorescence protein expression driven by the endogenous *gep1* promoter in ABS and gametocytes of the ∆*gep1mScarlet* parasite. **f** Co-staining of GEP1 and α-Tubulin (male gametocyte specific) in the non-activated (NAG) *6HA*::*gep1* gametocytes. *x*/*y* in the figure is the number of cell displaying signal/the number of cell tested. **g** and **h**, P28 expression during in vitro gametocyte to ookinete differentiation. P28 expression is detected in female gametes, fertilized zygotes, and ookinetes in IFA (**g**) and western blot (**h**). mpa: minute post activation; hpa, hour post activation. **i** Day 7 midgut oocyst counts from mosquitoes infected with parasites, including 17XNL, ∆*gep1*, ∆*nek4*, or ∆*map2* parasite alone, as well as mixtures of ∆*gep1/*∆*nek*4, ∆*gep1/*∆*map*2, or ∆*map*2*/*∆*nek*4 parasites. ∆*nek*4 and ∆*map*2 are female and male gamete-defect parasites, respectively. *x*/*y* on the top is the number of mosquito containing oocyst/the number of mosquito dissected; Mosquito infection prevalence is shown above. Scale bar = 5 μm for all images in this figure. Experiments were independently repeated three times in **b**, **c**, **d**, **e**, **f**, **g**, and two times in **i**. Two-tailed unpaired Student’s *t* test in **i**.

GEP1 is a *Plasmodium*-specific protein with 905 residues and 14 predicted TMs (Fig. 2a). Previous transcriptomic study indicated the *gep1* gene is transcribed in gametocytes and ookinetes, but not asexual blood stages of *P. falciparum* and *P. berghei*^19,20^. To investigate protein expression and localization, we tagged the endogenous GEP1 with 6HA at N-terminus (Supplementary Fig. 1g, j), generating *6HA*::*gep1* parasite that had normal development throughout the life cycle (Supplementary Fig. 5a). The GEP1 protein is expressed in gametocytes and ookinetes, but not in asexual blood stages and other mosquito stages of the *6HA*::*gep1* parasite (Fig. 2b, c). We also tagged the GEP1 protein with quadruple Myc (4Myc) (Supplementary Fig. 1j, Supplementary Fig. 5b) and observed similar expression pattern in the *4Myc*::*gep1* parasite (Fig. 2d). In addition, mScarlet fluorescent signals driven by the endogenous *gep1* promoter were detected only in gametocytes, but not in asexual blood stages of the ∆*gep1mScarlet* parasite (Fig. 2e). Co-staining *6HA*::*gep1* gametocytes with anti-α-Tubulin (male gametocyte specific) and anti-HA antibody showed that GEP1 was expressed in both male and female gametocytes (Fig. 2f). Interestingly, GEP1 is not expressed in plasma membrane, but in punctate dots in the cytoplasm of gametocytes and ookinetes (Fig. 2b, d, f).

### GEP1 regulates both male and female gametogenesis

Because GEP1 is expressed in both male and female gametocytes, we asked whether GEP1 also regulates the gametogenesis of female gametocytes. P28 protein, a marker for female gamete^21^, is expressed in female gametes, fertilized zygotes, and ookinetes of 17XNL parasite, but not in the ∆*gep1* parasite 2 h after XA-stimulation (Fig. 2g, h), indicating that GEP1 depletion also cause defect in female gametogenesis. We next performed genetic crosses between ∆*gep1* and ∆*map2* (male gamete-deficient) or ∆*nek4* (female gamete-deficient) parasites^22,23^ (Supplementary Fig. 1j, k). No midgut oocyst was observed in mosquitoes from the ∆*gep1* × ∆*map2* or ∆*gep1* × ∆*nek4* cross day 7 post infection (pi), whereas the ∆*map2* × ∆*nek4* cross produced slightly fewer oocysts than the WT parasite (Fig. 2i), suggesting no functional male and female gametes in the ∆*gep1* parasite. Together, these results demonstrate that GEP1 is essential for both male and female gametogenesis.

The purified ∆*gep1* gametocytes had morphology indistinguishable from that of WT 17XNL parasite (Supplementary Fig. 6a); however, whether GEP1 depletion causes gametocyte death or affects the fitness of gametocytes remains to be determined. We analyzed cell viability by Trypan blue exclusion assay. No gametocyte of WT or ∆*gep1* parasites were stained by Trypan blue (Supplementary Fig. 6b). As a control, both gametocytes were stained after heating the parasites at 60 °C for 5 min. In addition, staining with propidium iodide (PI) also indicated that the ∆*gep1* gametocytes are viable (Supplementary Fig. 6c). To further confirm the observations, we disrupted the endogenous *gep1* in a *P. yoelii* reporter strain *DFsc7* that expressed GFP and mCherry in male and female gametocytes, respectively^24^ (Supplementary Fig. 1j, Supplementary Fig. 6d, e). The expressions of fluorescent proteins in both male and female gametocytes were comparable with those of the parental parasite (Supplementary Fig. 6f, g). These results suggest that GEP1-depleted gametocytes are viable, but lost the ability to produce functional male and female gametes.

### GEP1 depletion blocks PKG-mediated signaling

**Fig. 3:**
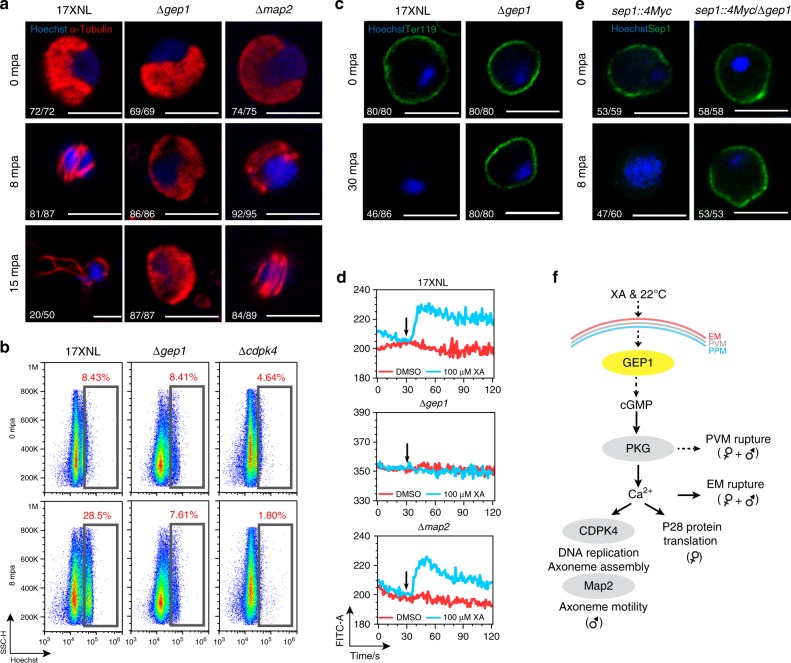
GEP1 acts upstream of PKG in the cGMP-PKG-Ca^2+^ signaling cascade. **a** α-Tubulin expression and distribution in differentiating male gametocytes from 17XNL, ∆*gep1* and ∆*map2* parasites after XA stimulation. mpa: minute post XA activation. **b** Flow cytometry analysis of genomic DNA content in XA-stimulated male gametocytes of 17XNL, ∆*gep1* and ∆*cdpk4* parasites. The parasites were fixed with 4% paraformaldehyde at indicated time and stained with Hoechst. **c** Representative images of gametocytes stained by anti-mouse TER119 antibody 0 and 30 min post XA stimulation (mpa). **d** Flow cytometry detection of cytosolic Ca^2+^ in gametocytes using Fluo-8 probe. Purified gametocytes were preloaded with Fluo-8, and signals were collected 30 s before addition of XA or DSMO. Black arrows indicate the time for DMSO or XA addition. **e** Representative IFA images of the *sep1::4Myc* and *sep1::4Myc/*∆*gep1* gametocytes stained by anti-Myc antibody. **f** Proposed location of GEP1 in the XA-PKG-Ca^2+^ signal cascade of gametogenesis. GEP1 depletion causes defect in both Ca^2+^-dependent and Ca^2+^-independent cellular events of gametogenesis. EM: erythrocyte membrane, PVM: parasitophorus vacuole membrane, PPM: parasite plasma membrane. *x*/*y* in **a**, **c**, and **e** are the number of cell displaying representative signal/the number of cell analyzed. Scale bar = 5 μm for all images in this figure. All experiments in this figure were repeated three times independently with similar results.

Upon stimulation, male gametocytes undergo tubulin polymerization into microtubules and three rounds of genome replication, resulting in release of eight flagellated gametes within 10–15 min^25^. The lack of exflagellation suggests defect in either axoneme assembly or egress from erythrocyte of the ∆*gep1* male gametes. Typical cytosolic distribution of *α*-Tubulin was observed in male gametocytes of WT, ∆*gep1*, and ∆*map2* parasites before XA stimulation (Fig. 3a). Assembled axonemes were formed and coiled around the nucleus of WT and ∆*map2* gametocytes 8 min post XA stimulation, but axoneme formation was not observed in the ∆*gep1* parasite (Fig. 3a). By 15 min, WT gametocytes released flagellated male gametes, but not ∆*map2* and ∆*gep1* gametocytes (Fig. 3a). Strikingly, *α*-Tubulin remained in cytosol of the ∆*gep1* male gametocytes (Fig. 3a). We also analyzed the genome replication in stimulated male gametocytes. Flow cytometry analysis of DNA content in Hoechst-stained gametocytes showed that fluorescence increased (from 8.4% to 28.5%) in WT, but not in the ∆*gep1* parasites (from 8.4% to 7.6%) after XA stimulation (Fig. 3b). As reported for *P. berghei*^13,22^, no genome replication occurs in the ∆*cdpk4* parasite (Fig. 3b, Supplementary Fig. 1j, k). These results show no axoneme assembly or mitotic division in the stimulated ∆*gep1* male gametocytes.

Differentiation of male and female gametes result in sequential rupture of PVM and EM for escaping from erythrocytes^2,26^. TER119 is a plasma membrane protein of mouse erythrocytes^27,28^, and anti-TER119 antibody showed no EM staining for stimulated WT male and female gametocytes (Fig. 3c). In contrast, intact EM was observed for the ∆*gep1* gametocytes 30 min post stimulation (Fig. 3c), indicating that GEP1 depletion affects EM lysis.

XA triggers a cytosolic Ca^2+^ mobilization event within 10–15 s post stimulation of gametocytes^13^, which is essential for gametes formation and EM rupture^11,13^. We next examined XA-stimulated Ca^2+^ mobilization in the ∆*gep1* gametocytes using Fluo-8 probe as described^29–31^. Fluo-8 did not affect the gametogenesis since WT gametocytes pre-loaded with Fluo-8 could form XA-stimulated ECs (Supplementary Fig. 7a) and responded to A23187, a Ca^2+^ ionophore^13^, in a dose-dependent manner using flow cytometry (Supplementary Fig. 7b). As expected, XA triggered a sharp increase in cytosolic Ca^2+^ signal in WT gametocytes, reaching maximal levels 10–15 s post stimulation, which resembled the observations in *P. berghei* using luminescence-based GFP::Aequorin sensor^13,15^. However, no Ca^2+^ response was detected in XA stimulated ∆*gep1* gametocytes (Fig. 3d). Ca^2+^ mobilization occurred in the ∆*map2* gametocytes as MAP2 functions downstream of Ca^2+^ signal^18,22^ (Fig. 3d).

Different from Ca^2+^-dependent EM rupture, PVM rupture is controlled by a Ca^2+^-independent mechanism^2^. To study PVM lysis, a parasite line *sep1::4Myc* was generated by C-terminally tagging a PVM protein SEP1 with 4Myc^27,28^ (Supplementary Fig. 1j). This parasite line developed normally throughout the life cycle (Supplementary Fig. 5e), indicating intact protein function of SEP1::4Myc. We next deleted the *gep1* gene in the *sep1::4Myc* parasite, generating *sep1::4Myc/*∆*gep1* mutant (Supplementary Fig. 1j). IFA showed lysis of Sep1::4Myc-labeled PVM in the *sep1::4Myc* gametocytes (Fig. 3e), while intact PVM was maintained in the *sep1::4Myc/*∆*gep1* gametocytes 8 min post XA stimulation (Fig. 3e), indicating no PVM lysis in stimulated ∆*gep1* gametocytes. Together, these results suggest that GEP1 functions upstream of PKG in XA-stimulated signaling cascade (Fig. 3f).

### Impaired cGMP synthesis in GEP1 deficient parasite

**Fig. 4:**
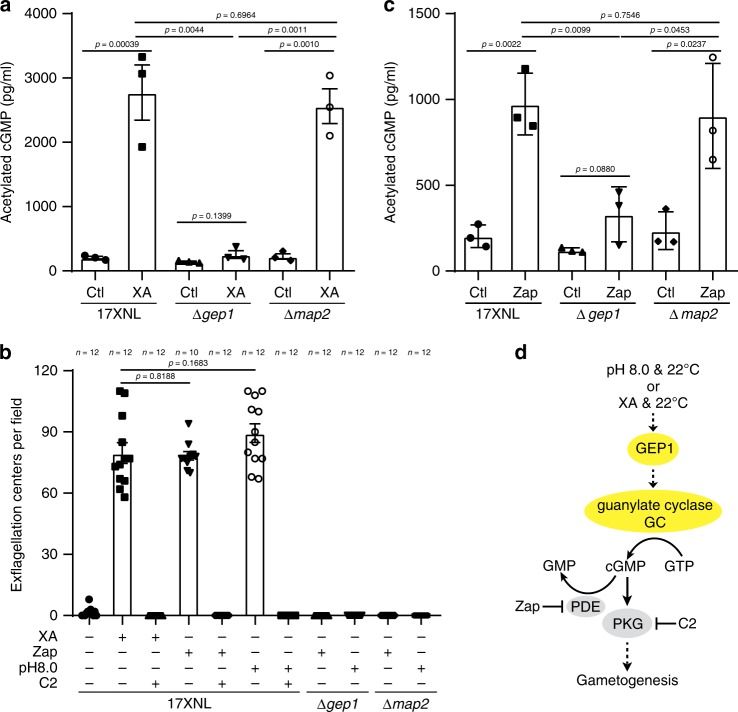
Impaired activity of cGMP synthesis in GEP1 deficient gametocytes. **a** Enzyme immunoassay detecting intracellular cGMP level in XA-stimulated gametocytes of the 17XNL, ∆*gep1*, and ∆*map2* parasites. Cells were incubated with 100 μM XA at 22 °C for 2 min before assay. Ctl are control groups without XA stimulation. **b** Exflagellation center counts of 17XNL, ∆*gep1*, and ∆*map2* parasites after treatment with XA (100 μM), Zaprinast (Zap, 100 μM), or pH 8.0 alone at 22 °C, or at the presence of compound 2 (C2, 5 μM). *n* is the numbers of microscopic fields counted (40×). **c** Enzyme immunoassay detecting intracellular cGMP level in Zap-treated gametocytes of the 17XNL, ∆*gep1*, and ∆*map2* parasites. Cells were incubated with 100 μM Zap at 22 °C for 2 min before assay. Ctl are control groups without Zap stimulation. **d** Proposed role of GEP1 in regulating cGMP synthesis activity of guanylyl cyclase in gametogenesis. All source data are provided as a Source Data file. Experiments in **a**, **b**, and **c** were repeated three times independently. Data are shown as mean ± SD; two-tailed unpaired Student’s *t* test.

Because cGMP is the direct upstream signal activating PKG in XA-stimulated gametogenesis^7,11,13,15^, we examined intracellular cGMP synthesis during gametogenesis. Purified gametocytes were stimulated with XA for 2 min, and cGMP levels were measured using an enzyme immunoassay^7,32^. Strikingly, XA induced a significant increase in cGMP level in WT gametocytes (Fig. 4a), consistent with previous observation in *P. falciparum*^7^. In contrast, the ∆*gep1* gametocytes failed to increase cGMP in response to XA stimulation (Fig. 4a). As a control, cGMP response occurred in ∆*map2* gametocytes because MAP2 functions downstream of both cGMP and Ca^2+^ signaling^18,22^. These results indicate that GEP1 regulates cGMP level, the most upstream intracellular signal known in *Plasmodium* gametogenesis.

cGMP level is tightly regulated by the opposing actions of cGMP-synthesizing GC and cGMP-hydrolyzing phosphodiesterase (PDE)^10,11,33^. Inhibition of PDE activity by specific inhibitor Zaprinast (Zap) has been shown to trigger *P. falciparum* gametogenesis in the absence of XA^11,33^. Indeed, treatment of WT gametocytes with 100 μM Zap also induced EC counts comparable to those induced by 50 μM XA (Fig. 4b), and gametogenesis stimulated by either XA or Zap could be blocked by a *Plasmodium* PKG protein inhibitor C2 (Fig. 4b), consistent with the established cGMP-PKG signal cascade of gametogenesis^14,15^. In contrast, the ∆*gep1* gametocytes failed to form ECs after treatment with Zap (Fig. 4b). No EC were observed in the control ∆*map2* gametocytes treated in either XA or Zap (Fig. 4b). Consistently, we examined the intracellular cGMP level in gametocytes treated with Zap for 2 min and detected significant increase in both WT and ∆*map2* gametocytes, but not in the ∆*gep1* gametocytes (Fig. 4c). Together, these results suggest that the GC activity for cGMP synthesis is impaired, and therefore no elevation of cGMP in the ∆*gep1* gametocytes after XA stimulation or Zap inhibition of PDE activity. In addition to XA and Zap, increasing pH from 7.4 to 8.0 has been reported to induce gametogenesis although the underlying mechanism is not clear^2,4^. Treating WT gametocytes with pH 8.0 at 22 °C indeed induced comparable number of ECs to those induced by XA or Zap (Fig. 4b), and gametogenesis could be blocked by C2 treatment (Fig. 4b)^15^, indicating that the signaling stimulated by pH 8.0 is also cGMP/PKG-dependent. However, pH 8.0 treatment could not induce gametogenesis of the ∆*gep1* gametocytes, further suggesting impaired activity of cGMP synthesis in GEP1 deficient parasite (Fig. 4d).

### GEP1 interacts and co-localizes with GCα

**Fig. 5:**
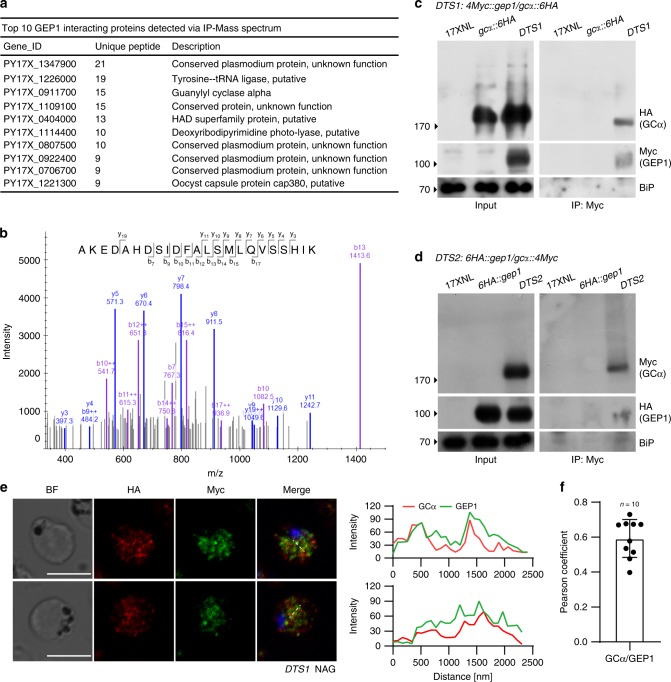
GEP1 interacts with GCα in gametocytes. **a** Top 10 GEP1 interacting proteins in the gametocytes of the *6HA::gep1* parasite detected by immunoprecipitation and mass spectrometry (MS), including guanylyl cyclase α (GCα) with 15 peptides detected. **b** MS2 spectrum of a representative peptide of the GCα protein. **c** Co-immunoprecipitation of Myc::GEP1 and GCα::HA proteins in gametocytes of the double tagged parasite *4Myc::gep1/gcα::6HA* (*DTS1*). IP-Myc, anti-Myc antibody was used. **d** Co-immunoprecipitation of HA::GEP1 and GCα::Myc proteins in gametocytes of the double tagged parasite *6HA::gep1/gcα:: 4Myc* (*DTS2*). IP-Myc, anti-Myc antibody was used. **e** Two-colored IFA of GEP1 and GCα proteins in the *DTS1* gametocytes using anti-HA (GCα) and anti-Myc (GEP1) antibodies (left panel). Cross sections (white dash line) of the cells show the co-localization of GEP1 and GCα (right panel). Scale bar = 5 μm. **f** Pearson coefficient analysis for GEP1 and GCα co-localization shown in **e**, data are shown as mean ± SD from *n* = 10 cells measured. Experiments in **c**, **d**, and **e** were repeated three times independently with similar results.

We next carried out immunoprecipitation and mass spectrometry experiments to identify molecules that may interact with GEP1 in gametocytes. By comparison of peptide signals (hits) between WT and *6HA::gep1* gametocyte samples from three biological replicates, we obtained 308 proteins that might interact with GEP1 (Supplementary Table 2), including GCα protein that is the enzyme presumably responsible for cGMP synthesis during gametogenesis (Fig. 5a, b)^8–10^. The *P. yoelii* GCα is a large protein (3850 amino acids) with 22 TMs distributed in an N-terminal P4-ATPase-like domain (ALD) and a C-terminal guanylate cyclase domain (GCD)^34,35^. To study the expression of GCα in gametocytes, we generated two parasite lines (*gcα::6HA* and *gcα::4Myc*) with endogenous GCα C-terminally tagged with 6HA and 4Myc, respectively (Supplementary Fig. 1j). These parasites developed normally in mouse and mosquito hosts (Supplementary Fig. 5c, d). Similar to GEP1, GCα was also expressed as cytoplasmic puncta in both male and female gametocytes of the *gcα::6HA* and *gcα::4Myc* parasites (Supplementary Fig. 8a). To further confirm the interaction between GEP1 and GCα, we generated a doubly tagged parasite line, *4Myc::gep1/gcα::6HA* (*DTS1*), by tagging the endogenous GEP1 with 4Myc in the *gcα::6HA* parasite (Supplementary Fig. 1j, Supplementary Fig. 5f–h). Results from immunoprecipitation using anti-Myc antibody indicated that GCα interacted with GEP1 in cell lysate of the *DTS1* gametocytes (Fig. 5c). We next generated another independent doubly tagged parasite, *6HA::gep1/gcα::4Myc* (*DTS2*) by tagging GCα with 4Myc in the *6HA::gep1* parasite (Supplementary Fig. 1j, Supplementary Fig. 5f–h) and detected similar interaction between GEP1 and GCα (Fig. 5d). As a control, no interaction between GEP1 and GCβ was detected in gametocytes of the *4Myc::gep1/gcβ::6HA* (*DTS3*) parasite (Supplementary Fig. 8b). These data demonstrate that GEP1 interacts with GCα in gametocytes. In addition, IFA results from the *DTS1* parasite showed that GEP1 and GCα are co-localized at cytosolic puncta in non-activated gametocytes (Fig. 5e, f). Together, these data suggest that GEP1 co-localizes and binds to GCα in gametocytes.

### GCα depletion causes defect in XA-stimulated gametogenesis

**Fig. 6:**
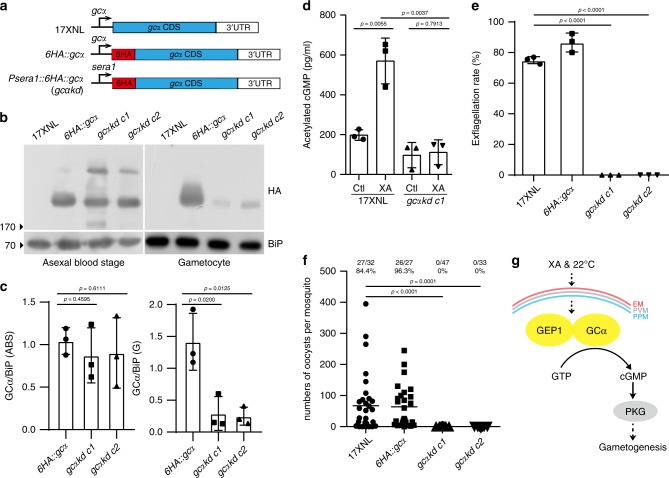
GCα knockdown in gametocytes results in gametogenesis defect. **a** Diagram showing a promoter swap strategy to knockdown *gcα* expression in gametocytes, generating HA-tagged *gcαkd* mutant with endogenous *gcα* promoter replaced with the *sera1* promoter. **b** Western blotting of GCα expression in asexual blood stages and gametocytes of the *gcαkd* parasite. The *6HA::gcα* as a control. **c** Quantitative analysis of GCα protein expression in **b**. **d** Intracellular cGMP level in XA-stimulated gametocytes of the 17XNL and *gcαkd* parasites. Cells were incubated with 100 μM XA at 22 °C for 2 min before assay. Ctl are control groups without XA stimulation. **e** In vitro exflagellation rates for 17XNL, *6HA::gcα*, and two clones of the *gcαkd* parasite after XA stimulation. **f** Day 7 midgut oocyst counts in mosquitos infected with 17XNL, *6HA::gcα*, and two clones of the *gcαkd* parasites. Mosquito infection prevalence is shown above. **g** A proposed model of GEP1/GCα interaction essential for XA-stimulated cGMP synthesis and gametogenesis. Experiments were independently repeated three times in **b**, **d**, **e**, and **f**. Data are shown as mean ± SD in **c**, **d**, and **e**. Two-tailed unpaired Student’s *t* test in **c**, **d**, **e**, and **f**. Source data of **c**, **d**, **e**, and **f** are provided as a Source Data file.

GCα has been implicated in cGMP synthesis during gametogenesis^8–10^; however, there has been no direct evidence to support the speculation. We attempted to disrupt the *gcα* gene but failed to obtain a GCα mutant parasite, indicating an essential function in asexual blood stage development, as reported in *P. falciparum* and *P. berghei* previously^10^. We used a promoter swap method described previously^36^ to replace 1322 bp of endogenous *gcα* promoter region with that (1626 bp) of *sera1* gene (PY17X_0305700) (Fig. 6a, Supplementary Fig. 1h), whose transcripts are expressed in asexual stages, but absent in gametocytes and mosquito stages^37^. In this editing, a 6HA tag was inserted in frame at the N-terminus of the GCα coding sequence. Correct modification in two parasite clones of the resulting mutant parasite *gcαkd* was confirmed by PCR (Supplementary Fig. 1j). The promoter replacement allowed expression of the GCα protein in asexual blood stages at a level comparable with that of another parallelly modified parasite *6HA::gcα* (Supplementary Fig. 1j), but significantly reduced GCα protein expression in gametocytes (Fig. 6b, c). Notably, the *gcαkd* parasite completely lost the ability to synthesize cGMP and form ECs after XA stimulation in vitro (Fig. 6d, e). In mosquitos fed with *gcαkd* parasite-infected mouse blood, no oocyst was detected in mosquito midgut (Fig. 6f). These results support that GCα is the GC responsible for XA-stimulated cGMP synthesis in gametogenesis (Fig. 6g). In addition, the phenotype caused by GCα knockdown in gametocytes resembles that of GEP1 defect.

Compared to the expression of GCα in both male and female gametocytes, GCβ expression was detected in *gcβ::6HA* female gametocytes only^8^ (Supplementary Fig. 8a, lower panel). In addition, GCβ depletion had no effect on XA-stimulated elevation of cGMP (Supplementary Fig. 8c) and in vitro EC formation (Supplementary Fig. 8d) in gametocytes of the ∆*gcβ* parasite^8^, in agreement with previous reports in *P. falciparum* and *P. berghei*^9,33^. These results exclude the involvement of GCβ in XA-stimulated cGMP signaling and gametogenesis.

### GEP1 depletion has no effect on GCα expression and localization

**Fig. 7:**
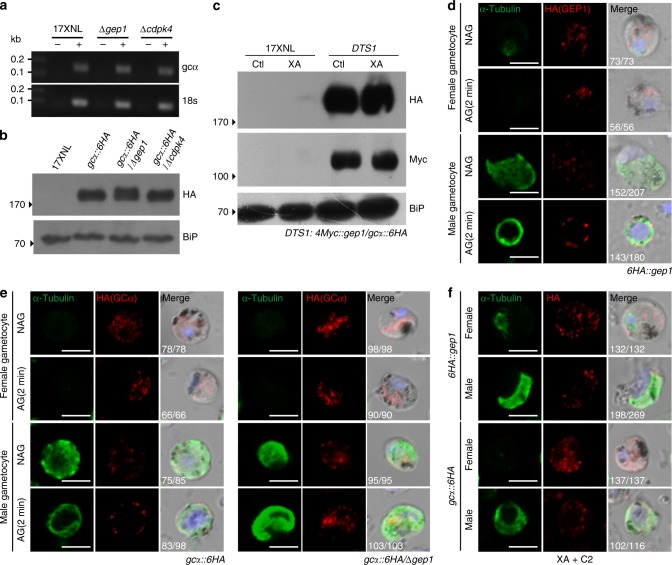
GCα expression and localization in the GEP1-depleted gametocytes. **a** RT-PCR analysis of *gcα* transcript in gametocytes of the 17XNL, ∆*gep1*, and ∆*cdpk4* parasites. **b** Western blotting detecting GCα protein in gametocytes of the 17XNL, *gcα::6HA, gcα::6HA/*∆*gep1*, and *gcα::6HA/*∆*cdpk4* parasites. **c** Western blotting detecting GEP1 (Myc) and GCα (HA) proteins expression in gametocytes of *DTS1* parasite 2 min post XA stimulation. Ctl are control groups without XA stimulation. **d** Co-staining of GEP1 and α-Tubulin expressions in gametocytes of the *6HA::gep1* parasite 2 min post XA stimulation. NAG: non-activated, AG: XA stimulation. **e** Co-staining of GCα and α-Tubulin expressions in the *gcα::6HA* and *6HA::gcα/*∆*gep1* gametocytes 2 min post XA stimulation. NAG: non-activated, AG: XA stimulation. **f** Co-staining of α-Tubulin and HA-tagged GEP1 or GCα expressions in the *6HA::gep1* (upper panel) and *gcα::6HA* (lower panel) gametocytes 2 min post XA stimulation plus C2 treatment. *x*/*y* in **d**, **e**, and **f** are the number of cell displaying representative signal/the number of cell analyzed. Scale bar = 5 μm for all images in this figure. All experiments in this figure were repeated three times independently.

As GCα and GEP1 interacted with each other and functioned upstream of cGMP signaling, we investigated whether GEP1 depletion would affect the expression and cellular localization of GCα in gametocytes. We deleted *gep1* gene in the *gcα::6HA* parasite, generating a *gcα::6HA/*∆*gep1* mutant parasite (Supplementary Fig. 1j, Supplementary Fig. 5i, j). GEP1 depletion had no effect on *gcα* mRNA level or GCα protein abundance in gametocytes of the *gcα::6HA/*∆*gep1* parasite compared to the parental parasite (Fig. 7a, b). As a control, depletion of CDPK4 had no effect on both mRNA and protein level of GCα either because CDPK4 functions downstream of cGMP signal (Fig. 7a, b). In addition, XA stimulation had no effect on protein abundance of both GEP1 and GCα in gametocytes of the *DTS1* parasite (Fig. 7c).

Next, we investigated the effect of XA stimulation in cellular localization of GEP1 and GCα proteins in gametocytes of the *6HA::gep1* or *gcα::6HA* parasite, respectively. Two minutes post XA stimulation, both GEP1 and GCα were expressed as cytoplasmic puncta in activated female gametocytes (Fig. 7d, e). Even 8 min post XA stimulation, both GEP1 and GCα still maintained in cytoplasmic puncta in activated female gametocytes (Supplementary Fig. 9a, b). Strikingly, both proteins were redistributed from cytoplasm to the cell periphery of activated male gametocytes 2 min post XA stimulation (Fig. 7d, e). We further investigated the localization of both GEP1 and GCα in activated gametocytes of the *DTS1* parasite. Two color IFA results indicate that GEP1 and GCα were co-localized in cytoplasm of activated female gametocytes but in cell periphery of activated male gametocytes 2 min post XA stimulation (Supplementary Fig. 9c, d), repeating the results from single color IFA. In activated male gametocytes, eight axonemes are assembled in the cytoplasm and coiled around the enlarged nucleus containing octaploid genome, likely pushing the cytosolic puncta to cell periphery. However, no redistribution of GCα was detected from cytoplasm to cell periphery in the stimulated *gcα::6HA/*∆*gep1* male gametocytes (Fig. 7e), which could be explained by no initiation of gametogenesis caused by GEP1 depletion. To further confirm the observations above, we treated the gametocytes with PKG inhibitor C2 to block the initiation of XA-stimulated gametogenesis. Indeed, no redistribution of either GEP1 or GCα was observed from cytoplasm to the cell periphery in the stimulated male gametocytes of the *6HA::gep1* and *gcα::6HA* parasite respectively (Fig. 7f). Together, these results indicate that GEP1 does not regulate the expression level and localization of GCα in non-activated male and female gametocytes, but affects the localizations of GCα in XA activated male gametocytes.

### XA stimulation likely enhances the GEP1/GCα interaction

**Fig. 8:**
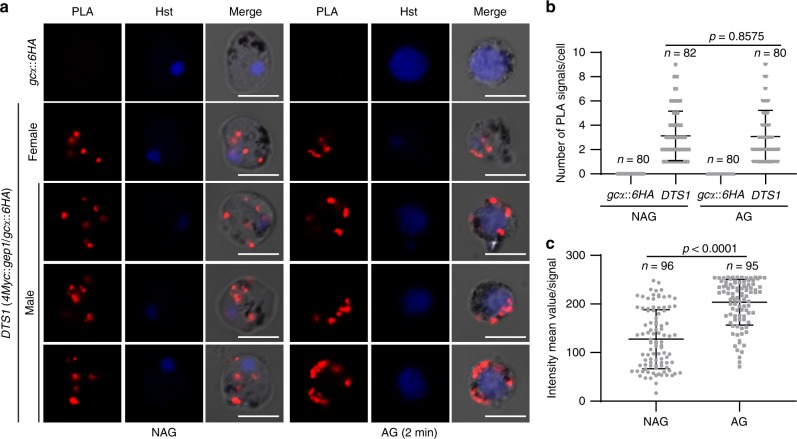
XA stimulation likely enhances the interaction between GEP1 and GCα. **a** Proximity Ligation Assay (PLA) detecting protein interaction between GEP1 and GCα in *DTS1* gametocytes. NAG: non-activated, AG: 2 min after XA stimulation. Activated male gametocytes were observed with enlarged nucleus containing replicated genome. Scale bar = 5 μm. **b** Number of PLA signal dot in each cell shown in **a**, *n* is the number of cells counted. **c** Fluorescence intensity value for each PLA signal dot shown in **a**. *n* is the number of PLA signal dot measured. Source data are provided as a Source Data file. Experiment was repeated three times independently. Data are shown as mean ± SD; two-tailed unpaired Student’s *t* test.

Lastly we asked whether XA stimulation could enhance the interaction between GEP1 and GCα in gametocytes. Proximity Ligation Assay (PLA) is a homogeneous immunohistochemical tool that couples the specificity of ELISA with the sensitivity of PCR, which allows in situ detection of endogenous proteins interaction with high specificity and sensitivity^38,39^. We performed the PLA to investigate the protein interaction in both non-activated gametocytes and activated gametocytes 2 min post XA stimulation. Robust PLA signals were detected in cytoplasm of the non-activated gametocytes of *DTS1* parasite when both anti-Myc and anti-HA primary antibodies were present (Fig. 8a), indicative of GEP1 and GCα interaction. As a control, no PLA signal was detected in gametocytes of the single tagged *gcα::6HA* parasite. 2 min post XA stimulation, the PLA signals were detected in cytoplasm of activated female gametocytes but in cell periphery of activated male gametocytes (Fig. 8a), which is consistent with the protein localization in IFA analysis (Fig. 7d, e, Supplementary Fig. 9c). Quantifying the number of PLA signal dots in each cells of gametocytes showed no difference between non-activated and activated gametocytes (Fig. 8b). However, the fluorescence intensity of PLA signal in the XA-activated gametocytes is significantly higher than that of the non-activated gametocytes (Fig. 8c), suggesting possible enhanced interaction between GEP1 and GCα in gametocytes after XA stimulation. We performed the PLA experiment in another independent doubly tagged parasite *DTS2* and observed the same results (Supplementary Fig. 10a–c).

## Discussion

It has been well-established that the XA-cGMP-PKG-Ca^2+^ signaling drives gametogenesis of *Plasmodium* parasites^7,11,13^ since the discovery of mosquito-derived XA as an inducer for gametogenesis more than two decades ago^5,6^. However, how the parasite senses external stimuli such as XA and reduction in environmental temperature to activate the cGMP signaling pathway remains unknown. In this study, we identified a membrane protein (GEP1) that responds to XA stimulation and binds to GCα, leading to activation of cGMP-PKG-Ca^2+^ signaling pathway and gametogenesis after functional screening 59 genes encoding integral membrane proteins expressed in gametocytes. Using CRISPR/Cas9 method, we successfully obtained gene deletion mutant parasites for 45 out of 59 candidate genes. To the best of our knowledge, our study is the first CRISPR/Cas9-based gene functional screening performed in malaria parasites, and the results from our CRISPR/Cas9-based screen largely matched the outcomes of a recent gene disruption screening using conventional homologous recombination in *P. berghei*^40^. Of the 45 genes, 25 orthologs of *P. berghei* were shown to be dispensable for asexual blood stage proliferation, 8 orthologs were resistant for disruption, and 12 orthologs were not tested in the screening of *P. berghei* (Supplementary Table 1)^40^. For the 14 disruption-resistant genes in our hands, all of the *P. berghei* orthologs also failed deletion attempts^40^.

After establishing the causative relationship of GEP1 deletion and gametogenesis defect, we investigated the position where GEP1 exerts its function in the XA-stimulated signaling cascade during gametogenesis. Previous studies have shown that cGMP enhances exflagellation of *P. berghei* and *P. falciparum*^41,42^. In addition, XA was shown to increase cGMP synthesis by GC from isolated membrane preparations of *P. falciparum* gametocytes^7^, suggesting that XA-stimulated gametogenesis is mediated by elevated GC activity and cGMP synthesis. Consistent with these observations, we detected significant increases in cytosolic cGMP level in WT gametocytes 2 min after XA stimulation, but not in ∆*gep1* gametocytes. GEP1 depletion resulted in impaired cGMP production in response to XA, indicating that GEP1 locates upstream of cGMP in the XA-cGMP-PKG-Ca^2+^ cascade. Compared with the 10–15 min required for whole process of gametogenesis, XA rapidly triggers a cytosolic Ca^2+^ mobilization within 10–15 s post stimulation, which was also observed in other studies^13^. These results suggest that GEP1 functions at an early or initiating step of gametogenesis. Consistently, disruption of *gep1* causes defects in all PKG-downstream cellular and signaling events during gametogenesis, including Tubulin polymerization for axoneme assembly, genome replication in male gametocytes, release of P28 translational repression in female gametocytes, PVM and EM rupture for egressing of both male and female gametes from erythrocytes, and Ca^2+^ mobilization. These results suggest that GEP1 functions upstream of cGMP-PKG-Ca^2+^ cascade in XA-stimulated gametogenesis.

The cytosolic cGMP level is balanced by the activities of cGMP-synthesizing GC and cGMP-hydrolyzing PDE^10,11,33^. That inhibition of PDE activity by inhibitor Zap could trigger gametogenesis in the absence of XA suggests the existence of low and sub-threshold endogenous cGMP level precluding PKG activation in gametocytes^11,33^. Strikingly, the ∆*gep1* gametocytes not only failed to initiate XA-stimulated gametogenesis, but also could not undergo Zap-induced gametogenesis. Consistently, we detected no significant Zap-induced elevation of cytosolic cGMP level in the ∆*gep1* gametocytes as seen in WT gametocytes. These results suggest that GEP1 is an essential component of the GC synthesis machinery, and its depletion completely impairs parasite ability to synthesize cGMP, resulting in no accumulation of basal level cGMP in gametocytes.

Two large guanylyl cyclases (GCα and GCβ) for cGMP synthesis are found in *Plasmodium* parasites^34^. GCα and GCβ in *P. yoelii* consist of 3850 and 3015 amino acids, respectively, and both proteins are predicted to have 22 TMs distributed in an N-terminal P4-ATPase-like domain (ALD) and a C-terminal guanylate cyclase domain (GCD). GC enzymes possessing the ALD/GCD structure are observed in many protozoan species^34,43^. Whereas the GCD is responsible for cGMP synthesis, the function of the ALD is still obscure. Both *P. berghei* and *P. falciparum* parasites without GCβ can produce functional male gametes^9,10^. Consistent with these reports, our study also showed deletion of *gcβ* did not affect XA-stimulated cGMP elevation and male gamete formation, confirming that GCβ is not the enzyme for cGMP synthesis during gametogenesis. Using unbiased immunoprecipitation and mass spectrometry analysis, we found that GEP1 interacted with GCα and this interaction was confirmed by co-immunoprecipitation and co-localization analyses. Furthermore, we attempted to disrupt the *gcα* gene, but were not able to obtain a viable mutant parasite, consistent with previous reports in other *Plasmodium* species^10^. Alternatively, we generated a mutant parasite with decreased GCα expression in gametocytes. Specific knockdown of GCα in gametocytes blocked XA-stimulated cGMP elevation and the consequent gametogenesis, mimicking the defect of GEP1 disruption. These results indicate that GCα is the enzyme for cGMP synthesis in gametogenesis.

Interestingly, GEP1 and GCα proteins were expressed as cytoplasmic puncta in female gametocytes either before or after XA stimulation. In the contrast, both proteins were redistributed from cytoplasm to the cell periphery of male gametocytes post XA stimulation. Once gametogenesis is initiated after XA stimulation, eight axonemes are assembled and coiled around the enlarged nucleus containing octaploid genome^18,22^, possibly occupying most cytoplasmic space and pushing cytoplasmic vesicles, including the GEP1/GCα residing puncta or possible membrane vesicle, to the periphery of the stimulated male gametocytes. Consistent with our observations, Carucci et al. also revealed that GCα displayed a peripheral localization in the *P. falciparum* stimulated gametocytes using immunoelectron microscopy^34^. In addition, these results also suggest that GEP1 likely exerts its function in controlling cGMP synthesis by directly binding GCα and regulating GCα conformation because GEP1 depletion had no effect in the expression and cellular localization of GCα in gametocytes.

GEP1 possesses 14 predicted TM domains, encoding a possible sodium-neurotransmitter symporter or amino acid transporter family protein. Three independent studies recently revealed that the *Toxoplasma gondii*, another Apicomplexan parasite, regulates natural egress of tachyzoites from host cell via a guanylate cyclase receptor platform^44–46^. Similar to *Plasmodium* GCα and GCβ, *T. gondii* guanylate cyclase (TgGC) also possesses the atypical ALD/GCD structure. By crosslinking experiment coupled to immunoprecipitation and mass spectrometry, 55 TgGC-interacting proteins were identified^44^, including a top 5th hit (TGGT1_208420) encoding a putative sodium-neurotransmitter symporter family protein. Notably, TGGT1_208420 displays some similarity in protein sequence with GEP1. These results suggest the interaction between GC and sodium-neurotransmitter symporter family protein is conserved in *Plasmodium* and *T. gondii*. Similar to *P. yoelii* GEP1, depletion of this protein does not cause tachyzoite growth defect^44^, suggesting a dispensable role in asexual lytic cycle of *T. gondii* although its function in sexual cycle is unknown. In addition, these studies also identified another *T. gondii* GC-interacting protein UGO that is believed to act as a chaperone^44^. Whether the *Plasmodium* UGO ortholog protein (PY17X_1204500) plays a similar role in the GC machinery remains to be determined.

Based on our results, we proposed a model for GEP1/GCα mediated cGMP signaling in XA-stimulated gametogenesis. The membrane protein GEP1 acts as a binding partner of GCα. In the absence of XA, GEP1 supports a functional conformation of GCα that maintains its basal catalytic activity and synthesizes low and sub-threshold endogenous cGMP level precluding PKG activation. In the presence of XA, the stimulation enhances the interaction of GEP1/GCα, leading to enhanced GC activity of GCα and increased cGMP level for PKG activation. In the GEP1-deficient gametocytes, GCα loses catalytic activity of cGMP synthesis and therefore fails to elevate cGMP level in response to XA, Zap treatment, or environmental pH. Currently, we could not exclude the possibility that there is an unknown molecule as the XA sensor residing in cytoplasm or plasma membrane and functioning upstream of GEP1/GCα complex. XA-stimulated gametocyte to gamete differentiation in the midgut is the first and essential step for mosquito transmission of malaria parasites, and elucidating the mechanisms involved may facilitate development of measures to block disease transmission.

## Methods

### Animal usage and ethics statement

Animal experiments were performed in accordance with the approved protocols (XMULAC20140004) by the Committee for Care and Use of Laboratory Animals of Xiamen University. ICR mice (female, 5 to 6 weeks old) were purchased and housed in the Animal Care Center of Xiamen University and kept at room temperature under a 12 h light/dark cycle at a constant relative humidity of 45%.

### Mosquito maintenance

The *Anopheles stephensi* mosquito (strain Hor) was reared at 28 °C, 80% relative humidity and at a 12 h light/dark cycle. Mosquitoes were fed on a 10% sucrose solution.

### Plasmid construction and parasite transfection

CRISPR/Cas9 plasmid pYCm was used for all the genetic modifications. For gene deleting, 5’-genomic and 3’-genomic segments (400 to 700 bp) of the target genes were amplified as left and right homologous arms, respectively, using gene specific primers (Supplementary Table 3). The PCR products were digested with appropriate restriction enzymes, and the digested products were inserted into matched restriction sites of pYCm. Oligonucleotides for sgRNAs were annealed and ligated into pYCm^17^. For each deletion modification, two sgRNAs were designed to disrupt the coding region of a target gene (Supplementary Table 3) using the online program ZiFit^47^. For gene tagging, a 400 to 800 bp segment from N-terminal or C-terminal of the coding region and 400 to 800 bp sequences from 5’UTR or 3’UTR of a target gene were amplified and fused with a DNA fragment encoding 6HA or 4Myc in frame at N-terminal or C-terminal of the gene. For each tagging modification, two sgRNAs were designed to target sites close to the C-terminal or N-terminal of the gene coding region. Infected red blood cells (iRBC) were electroporated with 5 μg circular plasmid DNA using Lonza Nucleofector. Transfected parasites were immediately injected *i.v*. into a naive mouse and treated with pyrimethamine (6 μg/ml) in drinking water. Parasites with transfected plasmids usually appear 5 to 7 days post drug selection.

### Genotype analysis of transgenic parasites

All transgenic parasites were generated from *P. yoelii* 17XNL strain or *P. berghei* ANKA strain. The schematic for different genetic modifications and the results of parasite transfection, single cloning and genetic verification of modified strains are summarized in Supplementary Fig. 1. Blood samples from infected mice were collected from the orbital sinus, and blood cells were lysed using 1% saponin in PBS. Parasite genomic DNAs were isolated from blood stage parasites using DNeasy Blood kits (QIAGEN). For each parasite, both 5’ and 3’ homologous recombination events were detected using specific PCR primers (Supplementary Fig. 1). PCR products from some modified parasites were DNA sequenced. All the primers used in this study are listed in Supplementary Table 3. Parasite clones with targeted modifications were obtained after limiting dilution. At least two clones for each gene-modified parasite were used for phenotype analysis. Parasite growth characteristics in mouse and in mosquito for the modified parasite strains are shown in Supplementary Fig. 5.

### Negative selection with 5-fluorouracil

Parasites subjected to sequential modifications were negatively selected with 5-Fluorouracil (5FC, Sigma, F6627) to remove episomal plasmid. 5FC (2 mg/ml) in drinking water was provided to mice in a dark bottle for 8 days with a change of drug on day 4. Clearance of episomal plasmid in parasites after negative selection was confirmed by checking the parasite survival after reapplying pyrimethamine pressure (6 μg/ml) in new infected mice.

### Gametocyte induction

ICR mice were treated with phenylhydrazine (80 μg/g mouse body weight) through intraperitoneal injection. Three days post treatment, the mice were infected with 3.0 × 10^6^ parasites through tail vein injection. Gametocytemia usually peaks at day 3 post infection. Male and female gametocytes were counted via Giemsa staining of thin blood smears. Gametocytemia was calculated as the ratio of male or female gametocyte over parasitized erythrocytes. All experiments were repeated three times independently.

### Male gametocyte exflagellation assay

Two and a half microliters of mouse tail blood with 4–6% gametocytemia were added to 100 μl exflagellation medium (RPMI 1640 supplemented with 10% fetal calf serum and 50 μM XA, pH 7.4) containing 1 μl of 200 units/ml heparin. After 10 min of incubation at 22 °C, the numbers of EC and RBC were counted in a hemocytometer under a light microscope. The percentage of RBCs containing male gametocytes was calculated from Giemsa-stained smears, and the number of ECs per 100 male gametocytes was then calculated as exflagellation rate. Compound 2 (5 μM) and Zaprinast (100 μM) were added to exflagellation medium with or without XA (for Zaprinast) to evaluate their effects on exflagellation.

### In vitro ookinete differentiation

In vitro culture for ookinete differentiation was prepared as described previously^13^. Briefly, mouse blood with 4–6% gametocytemia was collected in heparin tubes and immediately added to ookinete culture medium (RPMI 1640 medium containing 25 mM HEPES, 10% fetal calf serum, 100 μM XA, and pH 8.0) in a blood/medium volume ratio of 1:10. The cultures were incubated at 22 °C for 12 h to allow gametogenesis, fertilization, and ookinete differentiation. Ookinete formation was monitored by Giemsa-staining of culture smears. Ookinete conversion rate was calculated as the number of ookinetes (including mature and immature) per 100 female gametocytes.

### Mosquito feeding and transmission assay

Thirty female mosquitoes were allowed to feed on an anaesthetized mouse with 4–6% gametocytemia for 30 min. Mosquito midguts were dissected on day 7 post blood-feeding and stained with 0.1% mercurochrome for detection of oocyst. Salivary glands from 20–30 mosquitoes were dissected on day 14 post blood-feeding, and the number of sporozoites per mosquito was calculated.

### Parasite genetic cross

Genetic crosses between two different parasite lines were performed by infecting phenylhydrazine pre-treated mice with equal numbers of both parasites. Day 3 pi, 30 female mosquitoes were allowed to feed on mice carrying gametocytes for 30 min. Mosquito midguts were dissected on day 7 post blood-feeding and stained with 0.1% mercurochrome for oocyst counting.

### Gametocyte purification

Gametocytes were purified using the method described previously^48^. Briefly, mice were treated with phenylhydrazine 3 days before parasite infection. From day 3 pi, infected mouse were treated with sulfadiazine at 20 mg/l in drinking water to eliminate asexual blood stage parasites. After 48 h treatment with sulfadiazine, mouse blood containing gametocytes was collected from orbital sinus into a heparin tube. Gametocytes were separated from the uninfected erythrocyte by centrifugation using 48% Nycodenz solution (27.6% w/v Nycodenz in 5 mM Tris-HCl, 3 mM KCl, 0.3 mM EDTA, pH 7.2,) and prepared in gametocyte maintenance buffer (GMB, 137 mM NaCl, 4 mM KCl, 1 mM CaCl_2_, 20 mM glucose, 20 mM HEPES, 4 mM NaHCO_3_, pH 7.24–7.29, 0.1% BSA)^48^. Gametocytes were harvested from the interphase and washed three times in the GMB buffer. All the operations were performed at 19–22 °C.

### Trypan blue staining

Purified gametocytes were prepared in PBS and mixed with 0.4% trypan blue solution at a 1:9 volume ratio. The mixtures were incubated at room temperature for 5 min and examined under a light microscope.

### Propidium iodide staining

Purified gametocytes were prepared in PBS and stained with Propidium iodide (PI) at a final concentration of 50 μg/ml. The mixtures were incubated at room temperature for 10 min, washed with PBS twice, and then examined under a fluorescencec microscope.

### Flow cytometry analysis

For measuring DNA content in gametocytes, half of purified gametocytes were immediately fixed and half were transferred to exflagellation medium for gametogenesis for 8 min before fixation. Cells were fixed in 4% paraformaldehyde (PFA) for 20 min, washed in PBS and stained with Hoechst 33342 (0.5 μg/ml) for 30 min. Hoechst fluorescence signal of gametocytes was collected using Novocyte 3130 flow cytometer. For detecting GFP and mCherry in gametocytes, the gametocytes were stained with Hoechst 33342 and washed with PBS twice, GFP and mCherry fluorescence signal of gametocytes was collected using BD LSR Fortessa flow cytometer. Cell gating strategies are provided in Supplementary Fig. 11.

### Ca^2+^ mobilization assay using flow cytometry

Purified gametocytes were washed three times with Ca^2+^ free buffer (CFB, 137 mM NaCl, 4 mM KCl, 20 mM glucose, 20 mM HEPES, 4 mM NaHCO3, pH 7.2–7.3, 0.1% BSA) and then incubated in CFB containing 5 μM Fluo-8 at 37 °C for 20 min. Fluo-8 loaded gametocytes were washed twice with CFB and suspended in RPMI 1640 for flow cytometer analysis. Fluo-8 fluorescence signal reflecting cellular Ca^2+^ content in gametocytes were collected using BD LSR Fortessa flow cytometer. Signals were consecutively collected at 30 s before until 90 s post addition of XA (100 μM) or A23187 (0.1 and 1 μM). Cell gating strategies are provided in Supplementary Fig. 11.

### Detection of cellular cGMP

The assay for measuring cGMP levels in gametocytes was performed using a cyclic cGMP enzyme immunoassay kit (Cayman Chemical, #581021). For each test, more than 1.5 × 10^7^ gametocytes were collected and maintained in GMB buffer on ice. After treatment with 100 μM XA or 100 μM Zap for 2 min, cells were immediately lysed by 0.2 M cold hydrochloric acid on ice for 10 min, vortexed, and passed through a 22-gauge needle. For each replicate, three equal volumes of cell extract from each parasite preparation were parallel tested according to manufacturer’s instructions.

### Antibodies and antiserum

The primary antibodies used were: rabbit anti-HA (Western blot, 1:1000 dilution, IFA, 1:500 dilution) and rabbit anti-Myc (Western blot, 1:1000 dilution, IFA, 1:500 dilution) from Cell Signaling Technology; mouse anti-HA (IFA, 1:200) and mouse anti-Myc (IFA, 1:200) from Santa Cruz; mouse anti-α-Tubulin II from Sigma-Aldrich (IFA, 1:1000). The secondary antibodies used were: goat anti-rabbit IgG HRP-conjugated and goat anti-mouse IgG HRP-conjugated secondary antibodies from Abcam (1:5000); the Alexa 555 labeled goat anti-rabbit IgG, Alexa 555 labeled goat anti-mouse IgG, and Alexa 488 labeled goat anti-mouse IgG secondary antibodies from Thermo Fisher Scientific (1:500); Alexa 488 labeled anti-mouse TER-119 IgG antibody from BioLegend (IFA, 1:1000), biotinylated anti-rabbit IgG (H+L) antibody from Cell Signaling Technology (IFA, 1:1000); Streptavidin-ACP from Bioscience (IFA, 1:500). The anti-sera, including rabbit anti-Hep17 (Western blot, 1:1000), rabbit anti-P28 (Western blot, 1:1000, IFA, 1:1000), rabbit anti-BiP (Western blot, 1:1000) were prepared by immunization of synthetic peptides or recombinant protein as described previously^8^.

### Immunofluorescence assays

Purified parasites or chemical-treated parasites were fixed in 4% PFA and transferred onto a poly-L-Lysine pre-treated coverslip. The fixed cells were permeabilized with 0.1% Triton X-100 PBS solution for 7 min, blocked in 5% BSA solution for 60 min at room temperature or 4 °C overnight, and incubated with the primary antibodies diluted in PBS with 3% BSA at 4 °C for 12 h. The coverslip was incubated with fluorescently conjugated secondary antibodies. Cells were stained with Hoechst 33342, mounted in 90% glycerol solution, and sealed with nail polish. All images were captured and processed using identical settings on a Zeiss LSM 780 confocal microscope.

### Proximity ligtaion assay

The PLA assay detecting in situ protein interaction was performed using the kit (Sigma-Aldrich: DUO92008, DUO92001, DUO92005, and DUO82049). Non-activated and activated gametocytes were fixed with 4% PFA for 30 min, permeabilized with 0.1% Triton X-100 for 10 min, and blocked with a blocking solution overnight at 4 °C. The primary antibodies were diluted in the Duolink Antibody Diluent, added to the cells and then incubated in a humidity chamber overnight at 4 °C. The primary antibodies were removed and the slides were washed with Wash Buffer A twice. The PLUS and MINUS PLA probe were diluted in Duolink Antibody Diluent, added to the cells and incubated in a pre-heated humidity chamber for 1 h at 37 °C. Next, cells were washed with Wash Buffer A and incubated with the ligation solution for 30 min at 37 °C. Then, cells were washed with Wash Buffer A twice and incubated with the amplification solution for 100 min at 37 °C in the dark. Cells were washed with 1× Wash Buffer B twice and 0.01× Wash Buffer B once. Finally, cells were incubated with Hoechst 33342 and washed with PBS. Images were captured and processed using identical settings on a Zeiss LSM 780 confocal microscope.

### Protein extraction and western blotting

Proteins were extracted from asexual blood parasites and gametocytes using buffer A (0.1% SDS, 1 mM DTT, 50 mM NaCl, 20 mM Tris-HCl, pH 8.0) containing protease inhibitor cocktail and PMSF. After ultrasonication, the protein solution was kept on ice for 15 min before centrifugation at 14,000 × *g* for 10 min at 4 °C. The supernatant was lysed in Laemmli sample buffer. GEP1 protein was separated in 9% SDS-PAGE and transferred to PVDF membrane (Millipore, IPVH00010). GCα and GCβ proteins were separated in 4.5% SDS-PAGE. The membrane was blocked with TBST buffer (0.3 M NaCl, 20 mM Tris-HCl, 0.1% Tween 20, pH 8.0) containing 5% skim milk and incubated with primary antibodies. After incubation, the membrane was washed three times with TBST and incubated with HRP-conjugated secondary antibodies. The membrane was washed five times in TBST before enhanced chemiluminescence detection.

### Immunoprecipitation

For immunoprecipitation analysis, 6.0 × 10^7^ gametocytes were lysed in 1 ml protein extraction buffer A plus (0.01% SDS, 1 mM DTT, 50 mM NaCl, 20 mM Tris-HCl; pH8.0). After ultrasonication, the protein solution was incubated on ice for 15 min before centrifugation at 14,000 × *g* at 4 °C for 10 min. Rabbit anti-Myc antibody (1 μg, CST, #2272 s) or Rabbit anti-HA antibody (1 μg, CST, #3724 s) was added to the supernatant, and the solution was incubated on a vertical mixer at 4 °C for 15 h. After incubation, 20 μl buffer A plus pre-balanced protein A/G beads (Pierce, #20423) was added and incubated for 5 h. The beads were washed three times with buffer A plus before elution with Laemmli buffer.

### Mass spectrometry

After immunoprecipitation as described above, proteins were eluted twice with 0.3% SDS in 20 mM Tris-HCl (pH 8.0). Eluted proteins were precipitated using 20% trichloroacetic acid (TCA), washed twice with 1 ml cold acetone, and dried in centrifugation vacuum. The protein pellets were dissolved in buffer containing 1% SDC, 10 mM TCEP, 40 mM CAA, Tris-HCl pH 8.5 and were digested with trypsin (1:100 ratio) at 37 °C for 12–16 h after dilution with water to reduce SDS content to 0.5%. Peptides were desalted using SDB-RPS StageTips. For timsTOF Pro, an ultra-high pression nano-flow chromatography system (Elute UHPLC, Bruker) was coupled. Liquid chromatography was performed on a reversed-phase column (40 cm × 75 μm i.d.) at 50 °C packed with Magic C18 AQ 3-μm 200-Å resin with a pulled emitter tip. The timsTOF Pro was operated in PASEF mode^49^. Bruker.tdf raw files were converted to mgf files with the vendor provided software. The mgf files were searched against *P. yoelii* 17X genome database (downloaded from Uniprot) using PEAKS Studio X (BSI, Canada). Candidate peptides of targeted proteins were systematically validated by manual inspection of spectra.

### Bioinformatics analysis and tools

The genomic sequences of *Plasmodium* genes were downloaded from the *Plasmodium* database of PlasmoDB (http://plasmodb.org). Transmembrane domains of proteins were identified using the TMHMM Server (http://www.cbs.dtu.dk/services/TMHMM/). Multiple sequence alignments were performed by ClustalW in MEGA7.0 [41]. Flow cytometry data were analyzed using FlowJo v10.

### Quantification and statistical analysis

Statistical analysis was performed using GraphPad Software 8.0. Two-tailed Student’s *t*-test or Whiney Mann test was used to compare differences between treated groups. *P*-value in each statistical analysis was indicated within the figures.

### Reporting summary

Further information on research design is available in the Nature Research Reporting Summary linked to this article.

## Supplementary information

Supplementary Information

Reporting Summary

## Data Availability

The data supporting the findings of this study are available within the paper and its Supplementary Information files or are available from the corresponding author on reasonable request. The source data underlying Figs. 1a, e, f, 2i, 4a–c, 5e–f, 6c–f, 8b-c and Supplementary Figs. 2a, 3a–c, f, 5a–j, 6d-e, 8c-d, 9c-d, and 10b-c are provided as a Source Data file.

## Source Data

Source Data
