# Supplementary material for: An intracellular membrane protein GEP1 regulates xanthurenic acid induced gametogenesis of malaria parasites
Source: Nat Commun. 2020 Apr 9;11:1764. doi: 10.1038/s41467-020-15479-3 (PMC7145802; doi:10.1038/s41467-020-15479-3)
Supplement: Supplementary file 1 — Supplementary Information [file 41467_2020_15479_MOESM1_ESM.pdf]

Supplementary Information

An intracellular membrane protein GEP1 regulates xanthurenic acid induced gametogenesis of malaria parasites

Jiang et al.

1. Supplementary Figures 1-11 and figure legends

2. Supplementary Table 1. List of candidate gene for screening in this study

3. Supplementary Table 2. GEP1 interacted proteins detected by Mass spectrum

4. Supplementary Table 3. Primers and oligonucleotides used in this study

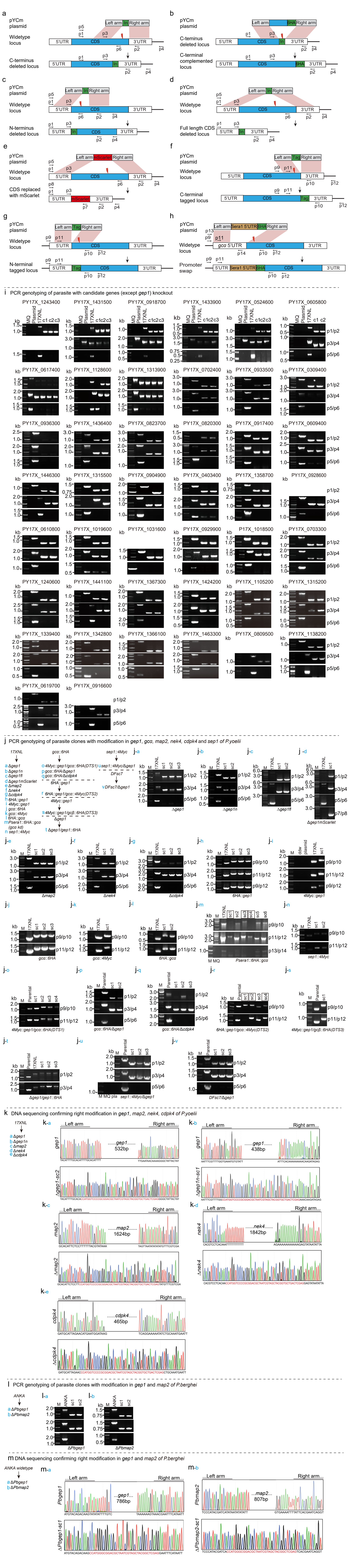

a
b
c
d
e
f
g
h
pYCm plasmid
Widetype locus
C-terminus deleted locus
C-terminal complemented locus
N-terminus deleted locus
Full length CDS deleted locus
CDS replaced with mScarlet
C-terminal tagged locus
N-terminal tagged locus
Promoter swap
Left arm
Right arm
5'UTR
3'UTR
CDS
i PCR genotyping of parasite with candidate genes (except gep1) knockout
p1/p2
p3/p4
p5/p6
j PCR genotyping of parasite clones with modification in gep1, gco, map2, nek4, cdpk4 and sep1 of P.yoelii
k DNA sequencing confirming right modification in gep1, map2, nek4, cdpk4 of P.yoelii
l PCR genotyping of parasite clones with modification in gep1 and map2 of P.berghei
m DNA sequencing confirming right modification in gep1 and map2 of P.berghei

Supplementary Fig. 1. Genotyping and DNA sequencing of modified parasites.

(a to h) Schematic for CRISPR/Cas9-mediated gene modification, including gene deletion in the C-terminus **(a)**, gene re-constitution in the C-terminal **(b)**, gene deletion in the N-terminus **(c)**, deletion of full length CDS **(d),** CDS replaced with mScarlet **(e)**, N-terminal **(f)** or C-terminal **(g)** tagging of genes with epitope tag, and promoter swap **(h)** via double cross homologous recombination. **(i to j** and **l)** For each modification, both 5' and 3' homologous recombination was detected using gene specific PCR pair (Supplementary Table 3) to confirm correct integration of the homologous template. At least two parasite clones (sc) for each modification were obtained after limiting dilution and phenotype analysis. **(k and m)** DNA sequencing to confirm correct modifications in some mutant parasites. Experiments in this figure were performed one time to ensure the correct genotype of the modified parasites.

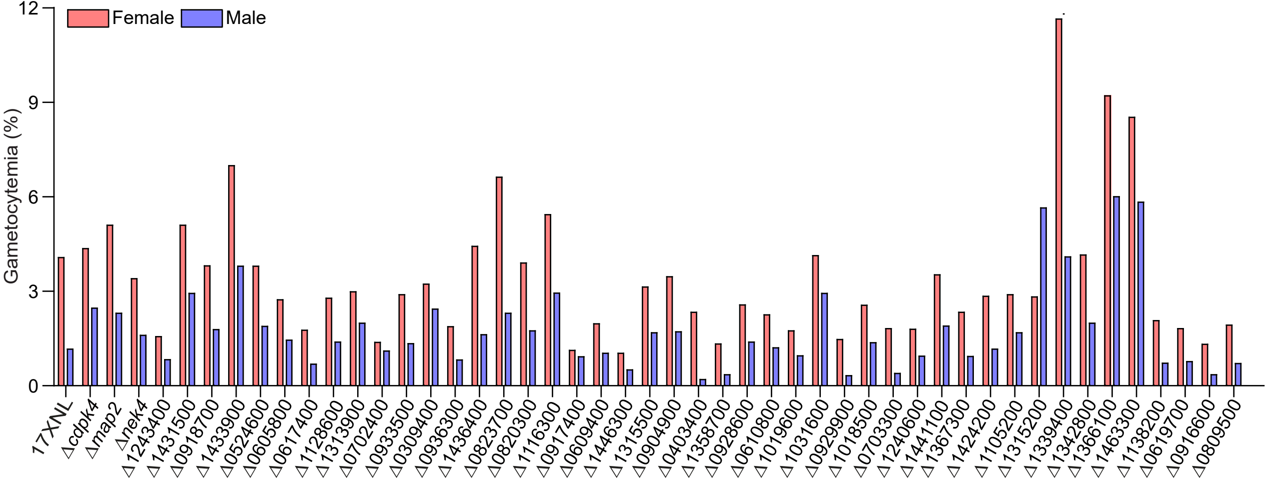
Female
Male
Gametocytemia (%)
12
9
6
3
0
17XNL
Δcdpk4
Δmap2
Δnek4
Δ1243400
Δ1431500
Δ0918700
Δ1433900
Δ0524600
Δ0605800
Δ0617400
Δ1128600
Δ1313900
Δ0702400
Δ0933500
Δ0309400
Δ0936300
Δ1436400
Δ0823700
Δ0820300
Δ1116300
Δ0917400
Δ0609400
Δ1446300
Δ1315500
Δ0904900
Δ0403400
Δ1358700
Δ0928600
Δ0610800
Δ1019600
Δ1031600
Δ0929900
Δ1018500
Δ0703300
Δ1240600
Δ1441100
Δ1367300
Δ1424200
Δ1105200
Δ1315200
Δ1339400
Δ1342800
Δ1366100
Δ1463300
Δ1138200
Δ0619700
Δ0916600
Δ0809500

Supplementary Fig. 2. Gametocyte formation in the mice.

The male and female gametocytemia in mice infected with 17XNL and 45 mutant parasites with candidate gene disrupted. The numbers shown in X axis are gene names (gene IDs) derived from the PlasmoDB database. Experiment was performed one time to ensure the formation of gametocyte in the mutants tested.

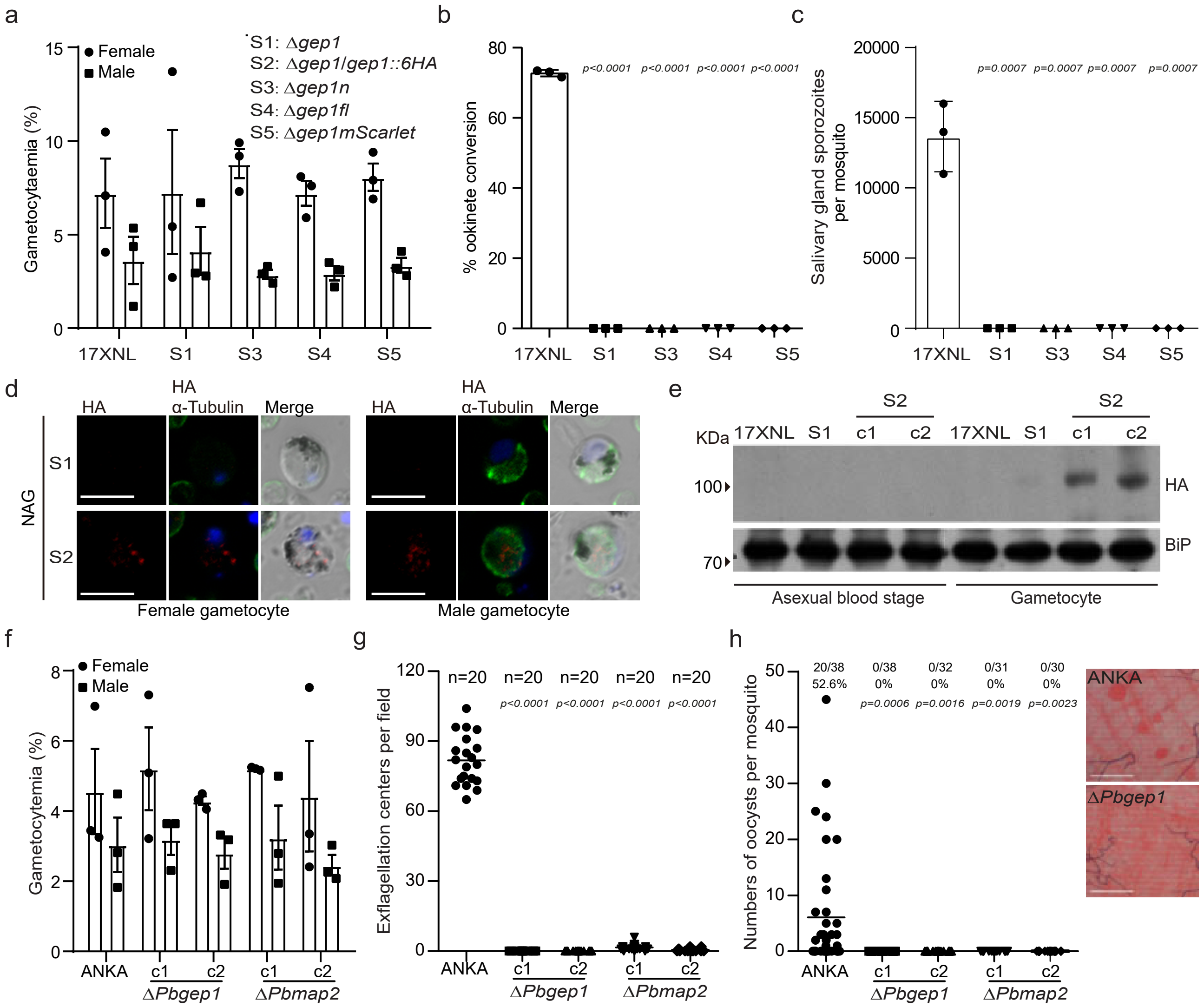

a
S1: Δgep1
S2: Δgep1/gep1::6HA
S3: Δgep1n
S4: Δgep1fl
S5: Δgep1mScarlet
Female
Male
Gametocytaemia (%)
17XNL S1 S3 S4 S5
b
% ookinete conversion
p<0.0001 p<0.0001 p<0.0001 p<0.0001
17XNL S1 S3 S4 S5
c
Salivary gland sporozoites per mosquito
p=0.0007 p=0.0007 p=0.0007 p=0.0007
17XNL S1 S3 S4 S5
d
HA
HA α-Tubulin
Merge
NAG
S1
S2
Female gametocyte
Male gametocyte
e
KDa
17XNL S1 S2 c1 c2 17XNL S1 S2 c1 c2
100
70
HA
BiP
Asexual blood stage
Gametocyte
f
Female
Male
Gametocytemia (%)
ANKA c1 c2 ΔPbgep1 c1 c2 ΔPbmap2
g
n=20 n=20 n=20 n=20 n=20
p<0.0001 p<0.0001 p<0.0001 p<0.0001
Exflagellation centers per field
ANKA c1 c2 ΔPbgep1 c1 c2 ΔPbmap2
h
20/38 52.6%
0/38 0% p=0.0006
0/32 0% p=0.0016
0/31 0% p=0.0019
0/30 0% p=0.0023
Numbers of oocysts per mosquito
ANKA c1 c2 ΔPbgep1 c1 c2 ΔPbmap2
ANKA
ΔPbgep1

Supplementary Fig. 3. GEP1 disruption causes defect in gametogenesis.

a, Gametocytemia in mice infected with 17XNL or *gep1* mutant parasites: S1 (Δ*gep1*), deletion in C-terminus; S3 (Δ*gep1n*), deletion in N-terminus; S4 (Δ*gep1fl*), deletion of the full coding region; S5 (Δ*gep1mScarlet*), coding region replaced with *mScarlet* gene. **b**, *In vitro* ookinete conversion rates for 17XNL and the *gep1* mutants. **c**, Numbers of salivary gland sporozoites in mosquitoes 14 day after feeding on mice infected with the parasites. **d**, Co-staining of GEP1 and α-Tubulin (male gametocyte specific) in the non-activated gametocytes (NAG) of S1 and S2 parasites. Scale bar = 5 μm. **e**, Western blot of GEP1 in ABS and gametocytes of S1 and S2 parasites. c1/c2: two independent clones of S2 parasite. **f**, Gametocytemia in mice infected with *P. berghei* ANKA and parasite with disrupted *Pbgep1* (Δ*Pbgep1*) or *Pbmap2* (Δ*Pbmap2*). **g**, Numbers of exflagellation centers (ECs) per microscopic field (40X) for the parasites in **f**. n is the numbers of microscopic fields counted (40X). **h,** Day 7 midgut oocyst counts in mosquitoes infected with the parasites in **f**. x/y on the top are the number of mosquito containing oocyst / the number of mosquito dissected; the percentage number indicates the mosquito infection prevalence. two-tailed unpaired Student's t test. Experiments were independently repeated three times in **a**, **b**, **c**, **d**, **f**, **g**, and two times in **e** and **h** with similar results. Data are shown as mean ± SD; two-tailed unpaired Student's t test in **b**, **c**, **g** and **h**.

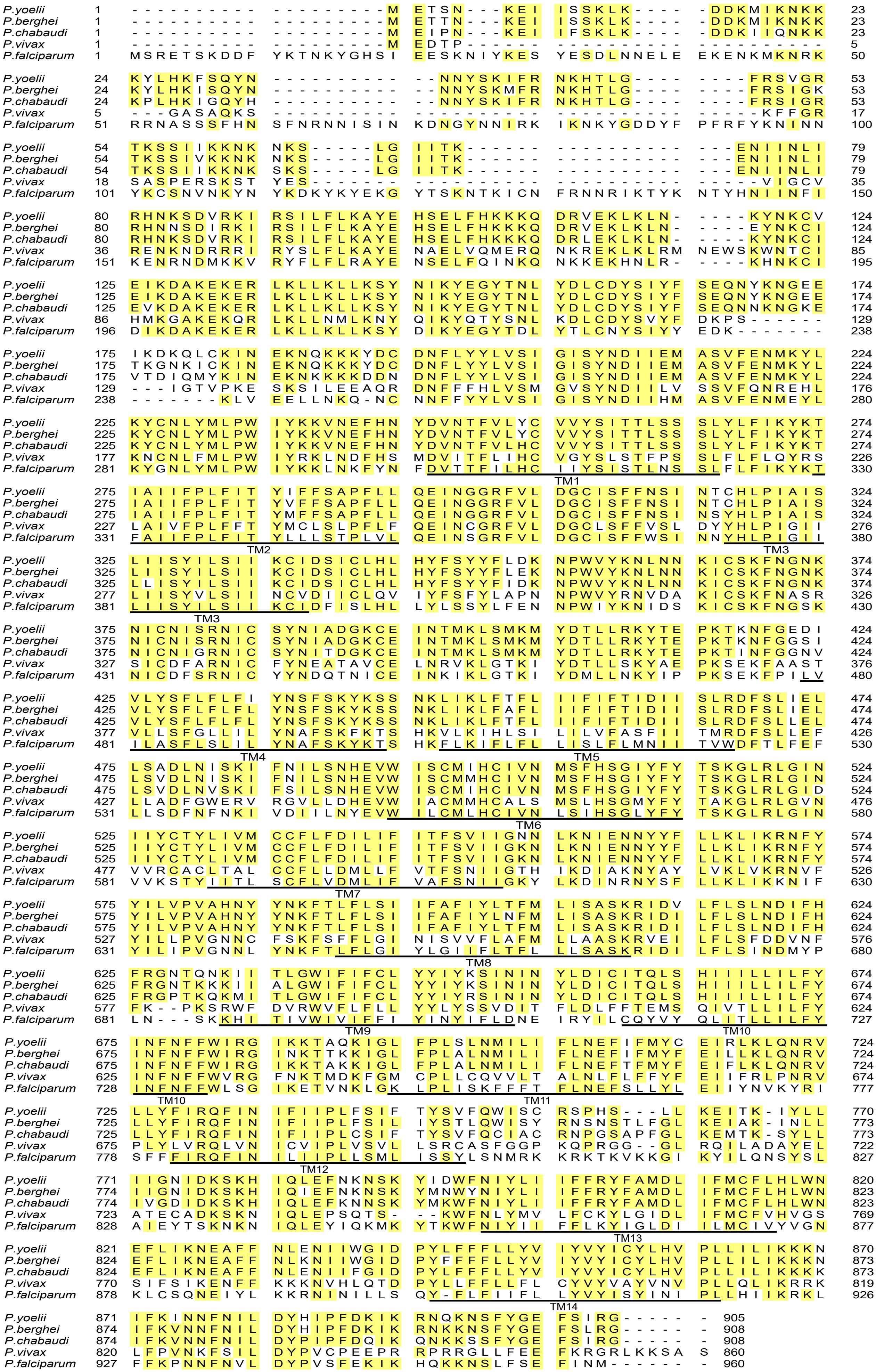

P.yoelii 1 ---------- ---------M ETSN---KEI ISSKLK---- -DDKMIKNKK 23
P.berghei 1 ---------- ---------M ETTN---KEI ISSKLK---- -DDKMIKNKK 23
P.chabaudi 1 ---------- ---------M EIPN---KEI IFSKLK---- -DDKIIQNKK 23
P.vivax 1 ---------- ---------M EDTP------ ---------- ---------- 5
P.falciparum 1 MSRETSKDDF YKTNKYGHSI EESKNIYKES YESDLNNELE EKENKMKNRK 50
P.yoelii 24 KYLHKFSQYN ---------- --NNYSKIFR NKHTLG---- ----FRSVGR 53
P.berghei 24 KYLHKISQYN ---------- --NNYSKMFR NKHTLG---- ----FRSIGK 53
P.chabaudi 24 KPLHKIGQYH ---------- --NNYSKIFR NKHTLG---- ----FRSIGR 53
P.vivax 5 ---GASAQKS ---------- ---------- ---------- -----KFFGR 17
P.falciparum 51 RRNASSSFHN SFNRNNISIN KDNGYNNIRK IKNKYGDDYF PFRFYKNINN 100
P.yoelii 54 TKSSIIKKNK NKS-----LG IITK------ ---------- ---ENIINLI 79
P.berghei 54 TKSSIVKKNK NKS-----LG IITK------ ---------- ---ENIINLI 79
P.chabaudi 54 TKSSIIKKNK SKS-----LG IITK------ ---------- ---ENIINLI 79
P.vivax 18 SASPERSKST YES------- ---------- ---------- -----VIGCV 35
P.falciparum 101 YKCSNVNKYN YKDKYKYEKG YTSKNTKICN FRNNRIKTYK NTYHNIINFI 150
P.yoelii 80 RHNKSDVRKI RSILFLKAYE HSELFHKKKQ DRVEKLKLN- ----KYNKCV 124
P.berghei 80 RHNNSDIRKI RSILFLKAYE HSELFHKKKQ DRVEKLKLN- ----EYNKCI 124
P.chabaudi 80 RHNKSDVRKI RSILFLKAYE HSELFHKKKQ DRLEKLKLN- ----KYNKCI 124
P.vivax 36 RENKNDRRRI RYSLFLKAYE NAELVQMERQ NKREKLKLRM NEWSKWNTCI 85
P.falciparum 151 KENRNDMKKV RYFLFLRAYE NSELFQINKQ NKKEKHNLR- ----KHNKCI 195
P.yoelii 125 EIKDAKEKER LKLLKLLKSY NIKYEGYTNL YDLCDYSIYF SEQNYKNGEE 174
P.berghei 125 EIKDAKEKER LKLLKLLKSY NIKYEGYTNL YDLCDYSIYF SEQNYKNGEE 174
P.chabaudi 125 EVKDAKEKER LKLLKLLKSY NIKYEGYTNL YDLCDYSIYF SEQNNKNGKE 174
P.vivax 86 HMKGAKEKQR LKLLNMLKNY QIKYQTYSNL KDLCDYSVYF DKPS------ 129
P.falciparum 196 DIKDAKEKER LKLLKLLKSY DIKYEGYTDL YTLCNYSIYY EDK------- 238
P.yoelii 175 IKDKQLCKIN EKNQKKKYDC DNFLYYLVSI GISYNDIIEM ASVFENMKYL 224
P.berghei 175 TKGNKICKIN EKNQKKKYDC DNFLYYLVSI GISYNDIIEM ASVFENMKYL 224
P.chabaudi 175 VTDIQMYKIN EKNKKKKDDN DNFLYYLVSI GISYNDIIEM ASVFENMKYL 224
P.vivax 129 ---IGTVPKE SKSILEEAQR DNFFFHLVSM GVSYNDIILV SSVFQNREHL 176
P.falciparum 238 -------KLV EELLNKQ-NC NNFFYYLVSI GISYNDIIHM ASVFENMEYL 280
P.yoelii 225 KYCNLYMLPW IYKKVNEFHN YDVNTFVLYC VVYSITTLSS SLYLFIKYKT 274
P.berghei 225 KYCNLYMLPW IYKKVNEFHN YDVNTFVLYC VVYSITTLSS SLYLFIKYKT 274
P.chabaudi 225 KYCNLYMLPW IYKKVNEFHN YDVNTFVLHC VVYSITTLSS SLYLFIKYKT 274
P.vivax 177 KNCNLFMLPW IYRKLNDFHS MDVITFLIHC VGYSLSTFPS SLFLFLQYRS 226
P.falciparum 281 KYGNLYMLPW IYKKLNKFYN FDVTTFILHC IIYSISTLNS SLFLFIKYKT 330
TM1
P.yoelii 275 IAIIFPLFIT YIFFSAPFLL QEINGGRFVL DGCISFFNSI NTCHLPIAIS 324
P.berghei 275 IAIIFPLFIT YVFFSAPFLL QEINGGRFVL DGCISFFNSI NTCHLPIAIS 324
P.chabaudi 275 IAIIFPLFIT YMFFSAPFLL QEINGGRFVL DGCISFFNSI NSYHLPIAIS 324
P.vivax 227 LAIVFPLFFT YMCLSLPFLF QEINCGRFVL DGCLSFFVSL DYYHLPIGII 276
P.falciparum 331 FAIIFPLFIT YLLLSTPLVL QEINSGRFVL DGCISFFWSI NNYHLPIGII 380
TM2
TM3
P.yoelii 325 LIISYILSII KCIDSICLHL HYFSYYFLDK NPWVYKNLNN KICSKFNGNK 374
P.berghei 325 LIISYILSII KCIDSICLHL HYFSYYFLEK NPWVYKNLNN KICSKFNGNK 374
P.chabaudi 325 LLISYILSII KCIDSICLHL HYFSYYFIDK NPWVYKNLNN KICSKFNGNK 374
P.vivax 277 LIISYVLSII NCVDIICLQV IYFSFYLAPN NPWVYRNVDA KICSKFNASR 326
P.falciparum 381 LIISYILSII KCIDFISLHL LYLSSYLFEN NPWIYKNIDS KICSKFNGSK 430
TM3
P.yoelii 375 NICNISRNIC SYNIADGKCE INTMKLSMKM YDTLLRKYTE PKTKNFGEDI 424
P.berghei 375 NICNISRNIC SYNIADGKCE INTMKLSMKM YDTLLRKYTE PKTKNFGGSI 424
P.chabaudi 375 NICNISRNIC SYNITDGKCE INTMKLSMKM YDTLLRKYTE PKTINFGGNV 424
P.vivax 327 SICDFARNIC FYNEATAVCE LNRVKLGTKI YDTLLSKYAE PKSEKFAAST 376
P.falciparum 431 NICDFSRNIC YYNDQTNICE INKIKLGTKI YDMLLNKYIP PKSEKFPILV 480
P.yoelii 425 VLYSFLFLFI YNSFSKYKSS NKLIKLFTFL IIFIFTIDII SLRDFSLIEL 474
P.berghei 425 VLYSFLFLFL YNSFSKYKSS NKLIKLFAFL IIFIFTIDII SLRDFSLIEL 474
P.chabaudi 425 VLYSFLFLFL YNSFSKYKSS NKLIKLFTFL IIFIFTIDII SLRDFSLLEL 474
P.vivax 377 VLLSFGLLIL YNAFSKFKTS HKVLKIHLSI LILVFASFII TMRDFSLLEF 426
P.falciparum 481 ILASFLSLIL YNAFSKYKTS HKFLKIFLFL LISLFLMNII TVWDFTLFEF 530
TM4
TM5
P.yoelii 475 LSADLNISKI FNILSNHEVW ISCMIHCIVN MSFHSGIYFY TSKGLRLGIN 524
P.berghei 475 LSVDLNISKI FNILSNHEVW ISCMMHCIVN MSFHSGIYFY TSKGLRLGIN 524
P.chabaudi 475 LSVDLNVSKI FSILSNHEVW ISCMIHCIVN MSFHSGIYFY TSKGLRLGID 524
P.vivax 427 LLADFGWERV RGVLLDHEVW IACMMHCALS MSLHSGMYFY TAKGLRLGVN 476
P.falciparum 531 LLSDFNFNKI VDIILNYEVW ILCMLHCIVN LSIHSGLYFY TSKGLRLGIN 580
TM6
P.yoelii 525 IIYCTYLIVM CCFLFDILIF ITFSVIIGNN LKNIENNYYF LLKLIKRNFY 574
P.berghei 525 IIYCTYLIVM CCFLFDILIF ITFSVIIGKN LKNIENNYYF LLKLIKRNFY 574
P.chabaudi 525 IIYCTYLIVM CCFLFDILIF ITFSVIIGKN LKNIENNYYF LLKLIKRNFY 574
P.vivax 477 VVRCACLTAL CCFLLDMLLF VTFSNIIGTH IKDIAKNYAY LVKLVKRNVF 526
P.falciparum 581 VVKSTYIITL SCFLVDMLIF VAFSNIIGKY LKDINRNYSF LLKLIKKNIF 630
TM7
P.yoelii 575 YILVPVAHNY YNKFTLFLSI IFAFIYLTFM LISASKRIDV LFLSLNDIFH 624
P.berghei 575 YILVPVAHNY YNKFTLFLSI IFAFIYLNFM LISASKRIDI LFLSLNDIFH 624
P.chabaudi 575 YILVPVAHNY YNKFTLFLSI IFAFIYLTFM LISASKRVDI LFLSLNDIFH 624
P.vivax 527 YILLPVGNNC FSKFSFFLGI NISVVFLAFM LLAASKRVEI LFLSFDDVNF 576
P.falciparum 631 YILIPVGNNL YNKFTLFLGI YLGIIFLTFL LLSASKRIDI LFLSINDMYP 680
TM8
P.yoelii 625 FRGNTQNKII TLGWIFIFCL YYIYKSININ YLDICITQLS HIIILLILFY 674
P.berghei 625 FRGNTKKKII ALGWIFIFCL YYIYKSININ YLDICITQLS HIIILLILFY 674
P.chabaudi 625 FRGPTKQKMI TLGWIFIFCL YYIYRSININ YLDICITQLS HIIILLILFY 674
P.vivax 577 FK--PKSRWF DVRWVFLFLL YYLYSSVDIT FLDLFFTEMS QIVTLLILFY 624
P.falciparum 681 LN---SKKHI TIVWIVIFFI YINYIFLDNE IRYILCQYVY QLITLLILFY 727
TM9
TM10
P.yoelii 675 INFNFFWIRG IKKTAQKIGL FPLSLNMILI FLNEFIFMYC EIRLKLQNRV 724
P.berghei 675 INFNFFWIRG INKTTKKIGL FPLALNMILI FLNEFIFMYF EILLKLQNRV 724
P.chabaudi 675 INFNFFWIRG IKKTAKKIGL FPLALNMILI FLNEFTFMYF EIFLKLQNRV 724
P.vivax 625 INFNFFWVRG FNKTMDKFGM CPLLCQVVLT ALNLFLFFYF EIIFRLPNRV 674
P.falciparum 728 INFNFFWLSG IKETVNKLGK LPLISKFFFT FLNEFSLLYL EIIYNVKYRI 777
TM10
TM11
P.yoelii 725 LLYFIRQFIN IFIIPLFSIF TYSVFQWISC RSPHS---LL KEITK-IYLL 770
P.berghei 725 LLYFIRQFIN IFIIPLFSIL IYSTLQWISY RNSNSTLFGL KEIAK-INLL 773
P.chabaudi 725 LLYFIRQFIN IFIIPLCSIF TYSVFQCIAC RNPGSAPFGL KEMTK-SYLL 773
P.vivax 675 PLYLVRQLVN ICVIPLVSVL LSRCASFGGP KQPRGG--GL RQILADAYEL 722
P.falciparum 778 SFFFIRQFIN ILIIPLLSML ISSYLSNMRK KRKTKVKKGI KYILQNSYSL 827
TM12
P.yoelii 771 IIGNIDKSKH IQLEFNKNSK YIDWFNIYLI IFFRYFAMDL IFMCFLHLWN 820
P.berghei 774 IIGNIDKSKH IQIEFNKNSK YMNWYNIYLI IFFRYFAMDL IFMCFLHLWN 823
P.chabaudi 774 IVGDIDKSKH IQLEFKNNSK YMKWFNIYLI IFFRYFAMDL IFMCFLHLWN 823
P.vivax 723 ATECADKSKN IQLEPSQTS- --KWFNLYMV LFCKYLGIDL IFMCFVHVGS 769
P.falciparum 828 AIEYTSKNKN IQLEYIQKMK YTKWFNIYII FFLKYIGLDI ILMCIVYVGN 877
TM13
P.yoelii 821 EFLIKNEAFF NLENIIWGID PYLFFFLLYV IYVYICYLHV PLLILIKKKN 870
P.berghei 824 EFLIKNEAFF NLKNIIWGID PYFFFFLLYV IYVYICYLHV PLLILIKKKN 873
P.chabaudi 824 EFLIKNEAFF NLENIIFGID PYLFFFLLYV IYVYICYLHV PLLILIKKKK 873
P.vivax 770 SIFSIKENFF KKKNVHLQTD PYLLFFLLFL CYVYVAYVNV PLLQLIKRRK 819
P.falciparum 878 KLCSQNEIYL KKRNINILLS QY-FLFIIFL LYVYISYINI PLLHIIKRKL 926
TM14
P.yoelii 871 IFKINNFNIL DYHIPFDKIK RNQKNSFYGE FSIRG----- - 905
P.berghei 874 IFKVNNFNIL DYHIPFDKIK RNKKNSFYGE FSLRG----- - 908
P.chabaudi 874 IFKVNNFNIL DYPIPFDQIK QNKKSSFYGE FSIRG----- - 908
P.vivax 820 LFPVNKFSIL DYPVCPEEPR RPRRGLLFEE FKRGRLKKSA S 860
P.falciparum 927 FFKPNNFNVL DYPVSFEKIK HQKKNSLFSE FINM------ - 960

Supplementary Fig. 4. GEP1 sequence alignment among *Plasmodium* parasites. Aligned GEP1 amino acid sequences from *P. falciparum*, *P. vivax*, *P. chabaudi*, *P. berghei*, and *P. yoelii* (GeneID in PlasmoDB: PF3D7_0515500, PVP01_1018400, PCHAS_1114700, PBANKA_1115100, PY17X_1116300). Fourteen predicted transmembrane domains (TM1- TM14) are underlined.

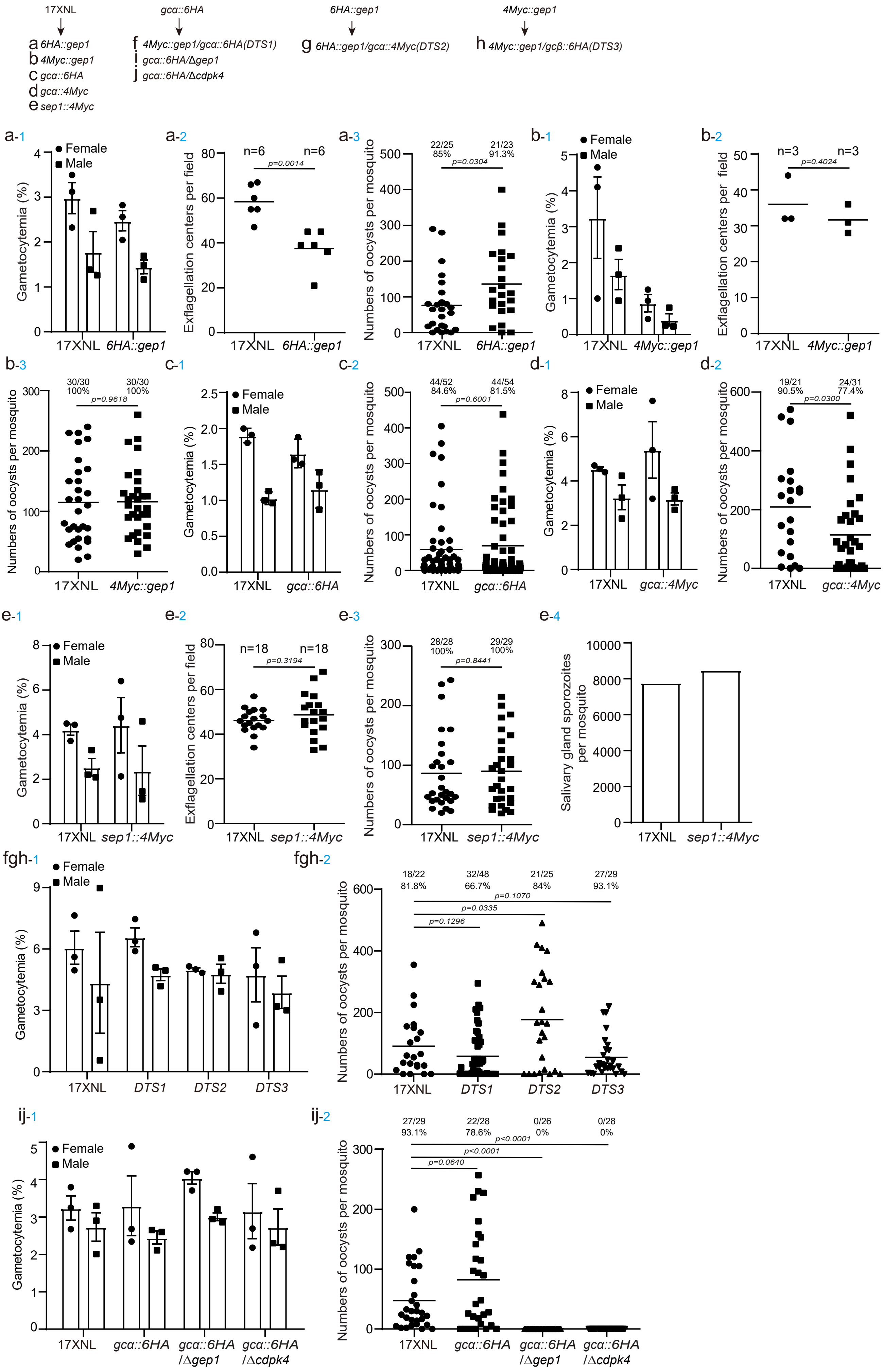
17XNL
gcα::6HA
6HA::gep1
4Myc::gep1
a 6HA::gep1
b 4Myc::gep1
c gcα::6HA
d gcα::4Myc
e sep1::4Myc
f 4Myc::gep1/gcα::6HA(DTS1)
i gcα::6HA/Δgep1
j gcα::6HA/Δcdpk4
g 6HA::gep1/gcα::4Myc(DTS2)
h 4Myc::gep1/gcβ::6HA(DTS3)
a-1
Female
Male
Gametocytemia (%)
17XNL 6HA::gep1
a-2
n=6 n=6
p=0.0014
Exflagellation centers per field
a-3
22/25 85%
21/23 91.3%
p=0.0304
Numbers of oocysts per mosquito
b-1
4Myc::gep1
b-2
n=3 n=3
p=0.4024
b-3
30/30 100%
30/30 100%
p=0.9618
c-1
c-2
44/52 84.6%
44/54 81.5%
p=0.6001
d-1
gcα::4Myc
d-2
19/21 90.5%
24/31 77.4%
p=0.0300
e-1
sep1::4Myc
e-2
n=18 n=18
p=0.3194
e-3
28/28 100%
29/29 100%
p=0.8441
e-4
Salivary gland sporozoites per mosquito
fgh-1
DTS1 DTS2 DTS3
fgh-2
18/22 81.8%
32/48 66.7%
21/25 84%
27/29 93.1%
p=0.1070
p=0.0335
p=0.1296
ij-1
gcα::6HA/Δgep1
gcα::6HA/Δcdpk4
ij-2
27/29 93.1%
22/28 78.6%
0/26 0%
0/28 0%
p<0.0001
p<0.0001
p=0.0640

Supplementary Fig. 5. Parasite growth in mouse and mosquito of some strains. Male and female gametocytes in mouse, *in vitro* exflagellation of male gametocytes, midgut oocyst formation and salivary gland sporozoite formation in mosquito of the modified parasites, including the *6HA::gep1* (**a**), *4Myc::gep1* (**b**), *gcα::6HA* (**c**), *gcα::4Myc* (**d**), *sep1::4Myc* (**e**), *DTS1* (**f**), *DTS2* (**g**), *DTS3* (**h**), and *gcα::6HA/Δgep1* (**i**, GEP1 disruption in the *gcα::6HA*) and *gcα::6HA/Δcdpk1* (**j**, CDPK1 disruption in the *gcα::6HA*). Gametocytemia count was repeated three times, exflagellation and mosquito infection was performed one time to ensure the normal life cycle progression of these parasites. n in the exflagellation experiments is the numbers of microscopic fields counted (40X). x/y on the top of the oocyst count is the number of mosquito containing oocyst / the number of mosquito dissected; the percentage number is the mosquito infection prevalence. Gametocytemia data are shown as mean ± SEM. Two-tailed unpaired Student's t test was used in the exflagellation and oocyst counting.

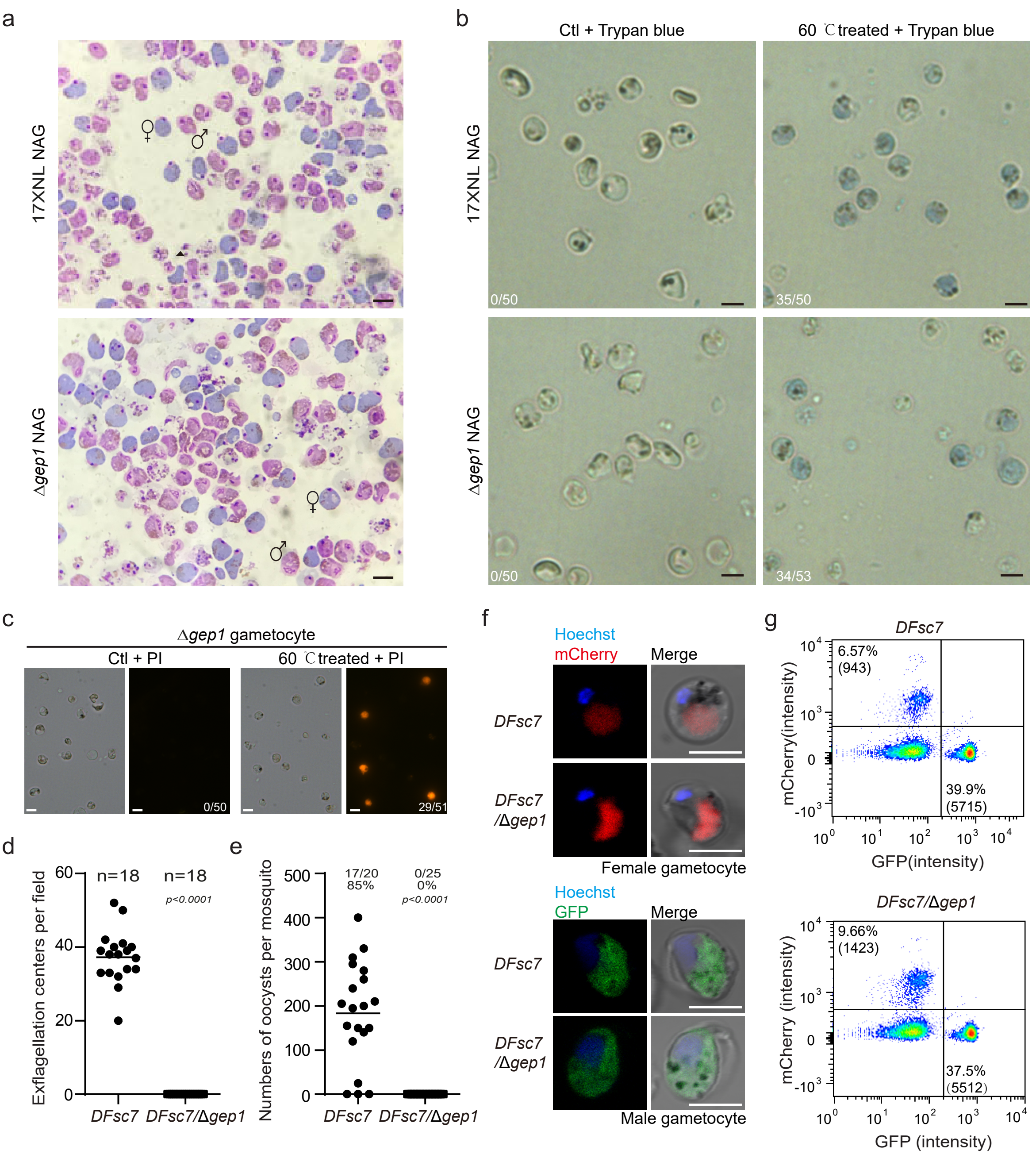

a
17XNL NAG
Δgep1 NAG
b
Ctl + Trypan blue
60 ℃ treated + Trypan blue
17XNL NAG
Δgep1 NAG
0/50
35/50
0/50
34/53
c
Δgep1 gametocyte
Ctl + PI
60 ℃ treated + PI
0/50
29/51
d
n=18
n=18
p<0.0001
Exflagellation centers per field
DFsc7
DFsc7/Δgep1
e
17/20
85%
0/25
0%
p<0.0001
Numbers of oocysts per mosquito
DFsc7
DFsc7/Δgep1
f
Hoechst
mCherry
Merge
DFsc7
DFsc7 /Δgep1
Female gametocyte
Hoechst
GFP
Merge
DFsc7
DFsc7 /Δgep1
Male gametocyte
g
DFsc7
6.57% (943)
39.9% (5715)
mCherry(intensity)
GFP(intensity)
DFsc7/Δgep1
9.66% (1423)
37.5% (5512)
mCherry (intensity)
GFP (intensity)

Supplementary Fig. 6. Gametocytes without GEP1 are viable.

a, Giemsa staining of the purified non-activated gametocytes (NAG). **b,** Cell viability analysis of the gametocytes by Trypan blue staining. **c,** Cell viability analysis of the Δ*gep1* gametocytes by propidium iodide (PI) staining. x/y in the **b** and **c** are the number of cell displaying signal / the number of cell counted. **d**, EC formation of the *DFsc7* and *DFsc7/*Δ*gep1* parasites after XA stimulation *in vitro*. n is the numbers of microscopic fields counted. **e,** Day 7 midgut oocysts counts from mosquitoes infected with *DFsc7* or *DFsc7/*Δ*gep1* parasites. Mosquito infection prevalence is shown above. **f,** GFP expression in male gametocytes and mCherry expression in female gametocytes of the *DFsc7* and *DFsc7/*Δ*gep1* parasites. **g,** Flow cytometry detection of GFP and mCherry fluorescence in male and female gametocytes of the *DFsc7* and *DFsc7/*Δ*gep1* parasites. Cell number and percentage are shown in the quadrants of the FACS plots. Scale bar = 5 μm for all images in this figure. Experiments in this figure were independently repeated three times with similar results. Two-tailed unpaired Student's t test in **d** and **e**.

a

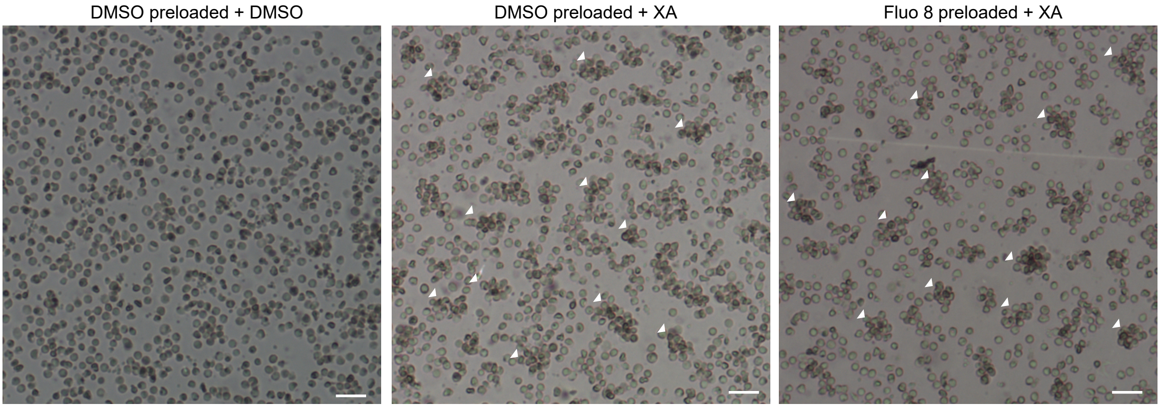

DMSO preloaded + DMSO
DMSO preloaded + XA
Fluo 8 preloaded + XA

b

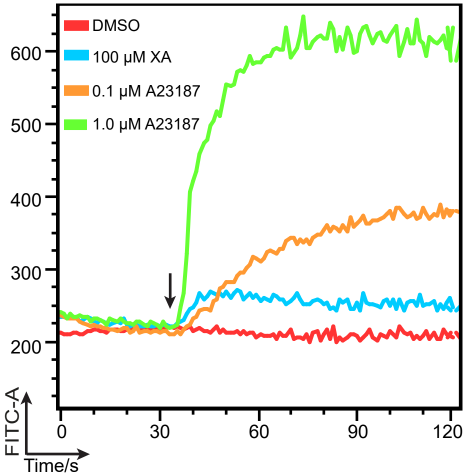

DMSO
100 μM XA
0.1 μM A23187
1.0 μM A23187
600
500
400
300
200
FITC-A
0
30
60
90
120
Time/s

Supplementary Fig. 7. Cellular Ca^{2+} mobilization in activated gametocytes.

a, WT gametocytes pre-loaded with Fluo-8 are capable of forming XA-stimulated ECs (white arrows) *in vitro*. Scale bar = 20 μm. **b,** Cellular Ca^{2+} signals in Fluo-8 pre-loaded WT gametocytes in response to A23187 (Ca^{2+} ionophore) and XA stimulation using flow cytometry analysis. Purified gametocytes were preloaded with Ca^{2+} probe Fluo-8, and signals were collected 30 seconds before addition of A23187, XA, or DSMO. Black arrows indicate the time for chemical addition. Experiments in this figure were independently repeated three times with similar results.

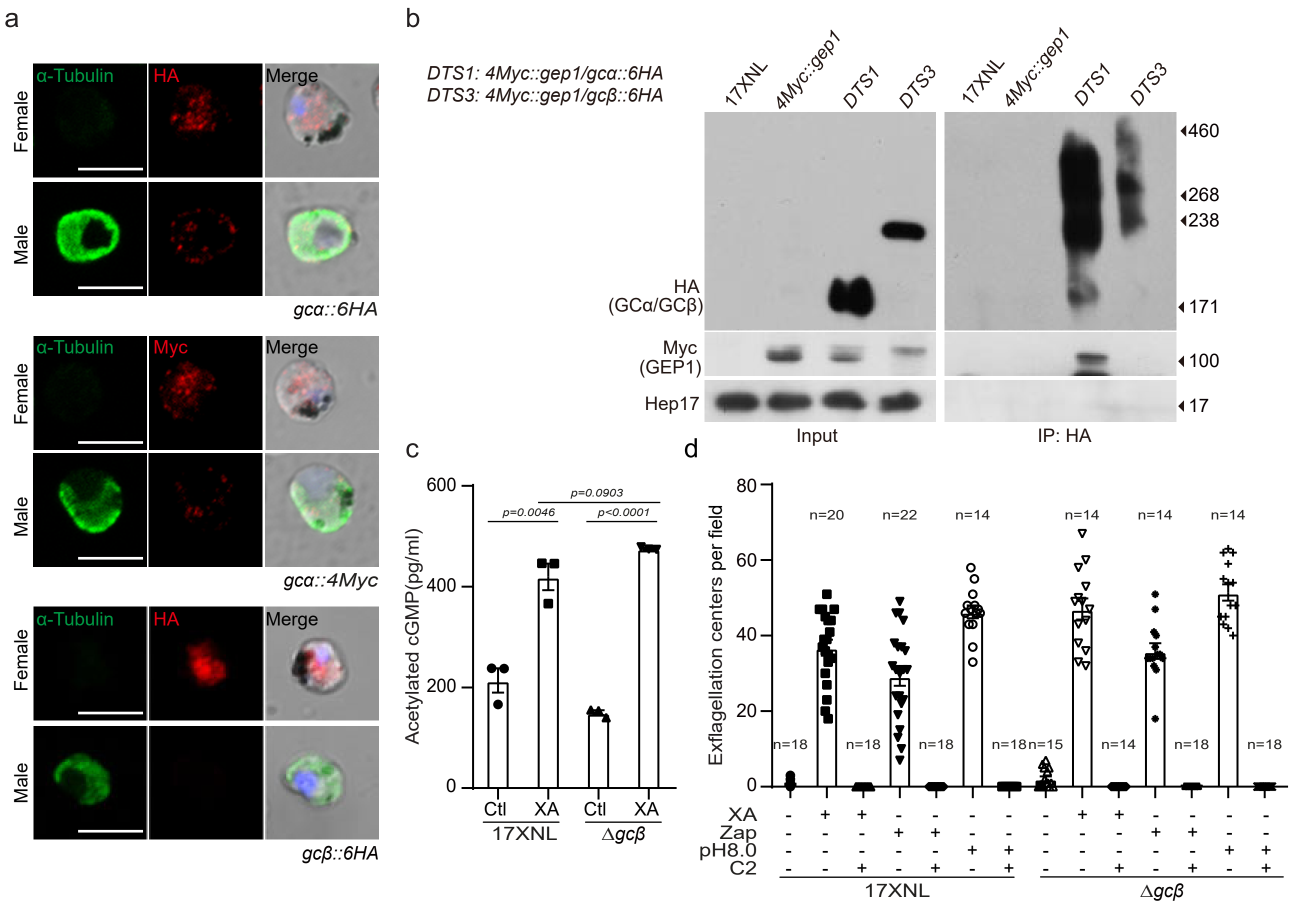

a
α-Tubulin
HA
Merge
Female
Male
gcα::6HA
Myc
gcα::4Myc
gcβ::6HA
b
DTS1: 4Myc::gep1/gcα::6HA
DTS3: 4Myc::gep1/gcβ::6HA
17XNL
4Myc::gep1
DTS1
DTS3
HA (GCα/GCβ)
Myc (GEP1)
Hep17
460
268
238
171
100
17
Input
IP: HA
c
Acetylated cGMP(pg/ml)
600
400
200
0
p=0.0903
p=0.0046
p<0.0001
Ctl
XA
17XNL
Δgcβ
d
Exflagellation centers per field
80
60
40
20
n=18
n=20
n=22
n=14
n=15
XA
Zap
pH8.0
C2

Supplementary Fig. 8. GCβ is not involved in cGMP signaling of gametogenesis. **a,** IFA of GCα and GCβ expression in female and male gametocytes of the *gcα::6HA*, *gcα::4Myc*, and *gcβ::6HA* parasites. α-Tubulin is a male gametocyte specific protein. Scale bar = 5 μm. **b,** Co-immunoprecipitation of GEP1 and GCβ in gametocyte extract of the *4Myc::gep1/gcβ::6HA* parasites (Double Tagged Strain 3, *DTS3*). IP-HA, anti-HA antibody was used in pulldown. **c**, Enzyme immunoassay detecting intracellular cGMP level in XA-stimulated gametocytes of the 17XNL and △*gcβ* parasites. Cells were incubated with 100 μM XA at 22°C for 2 min before assay. Ctl is control group without XA stimulation. **d**, *In vitro* EC formation of the 17XNL and △*gcβ* parasites after stimulation with XA (100 μM), Zaprinast (Zap, 100 μM), or pH 8.0 alone at 22°C, or conjugated with compound 2 (C2, 5 μM). Experiments in this figure were independently repeated three times. Data are represented as mean ± SEM in **c** and **d**, two-tailed unpaired Student's t test in **c**.

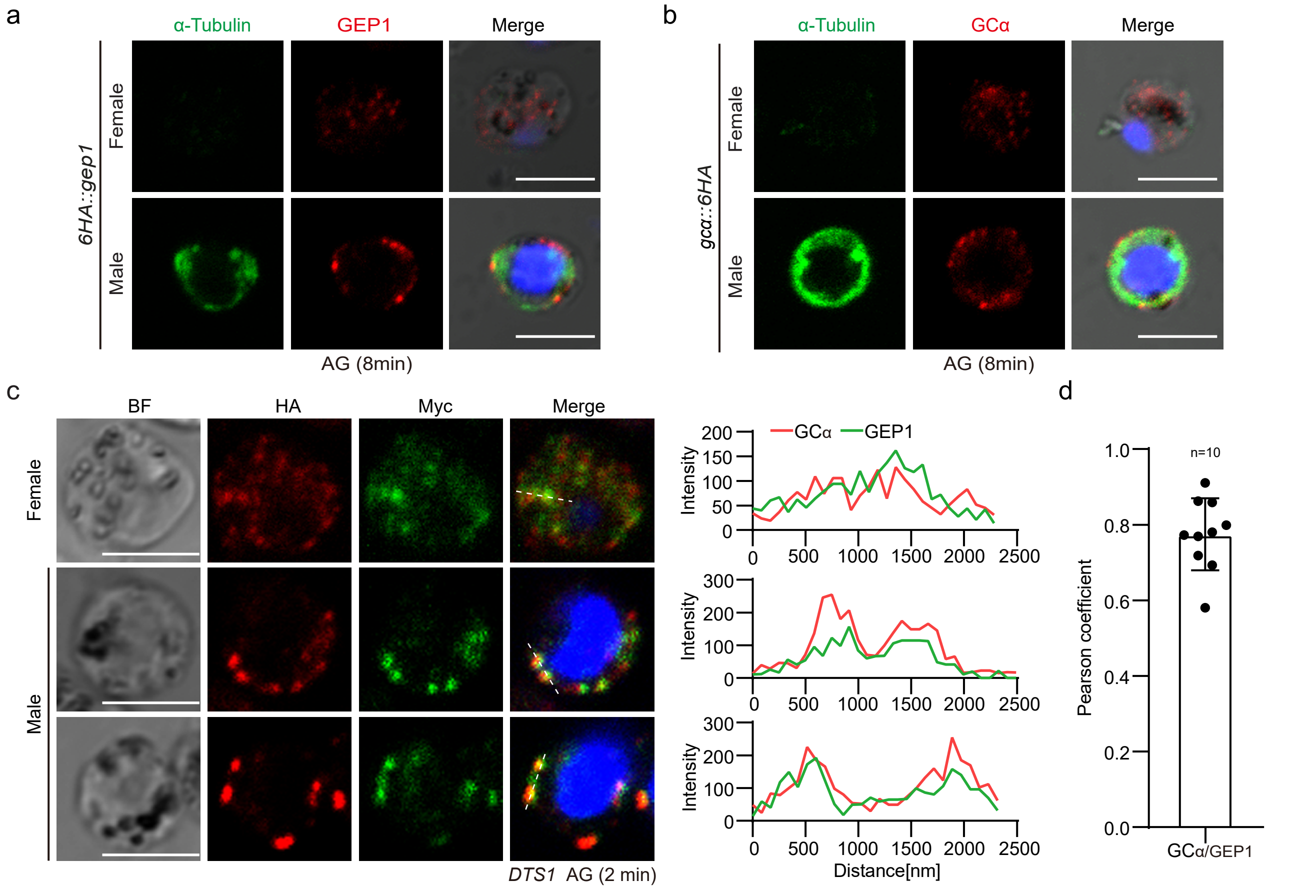

a
α-Tubulin
GEP1
Merge
6HA::gep1
Female
Male
AG (8min)
b
α-Tubulin
GCα
Merge
gcα::6HA
Female
Male
AG (8min)
c
BF
HA
Myc
Merge
Female
Male
DTS1 AG (2 min)
GCα
GEP1
Intensity
Distance[nm]
d
n=10
Pearson coefficient
GCα/GEP1

Supplementary Fig. 9. GEP1 co-localizes with GCα in activated gametocytes.

a, Co-staining of GEP1 and α-Tubulin expressions in the activated gametocytes (AG) of the *6HA::gep1* parasite 8 min post XA stimulation. **b**, Co-staining GCα and α-Tubulin in the *gcα::6HA* gametocytes 8 min post XA stimulation. **c**, Co-staining of GEP1 and GCα in the *DTS1* gametocytes 2 min post XA stimulation using anti-HA (GCα) and anti-Myc (GEP1) antibodies (left panel). Activated male gametocytes were observed with enlarged nucleus. Cross sections (white dash line) of the cells show the co-localization of GEP1 and GCα (right panel). Scale bar = 5 μm. **d**, Pearson coefficient analysis for GEP1 and GCα co-localization shown in **c**, data are shown as mean ± SD from n=10 cells measured. Scale bar = 5 μm for all image in this figure. Experiments in this figure were independently repeated three times with similar results.

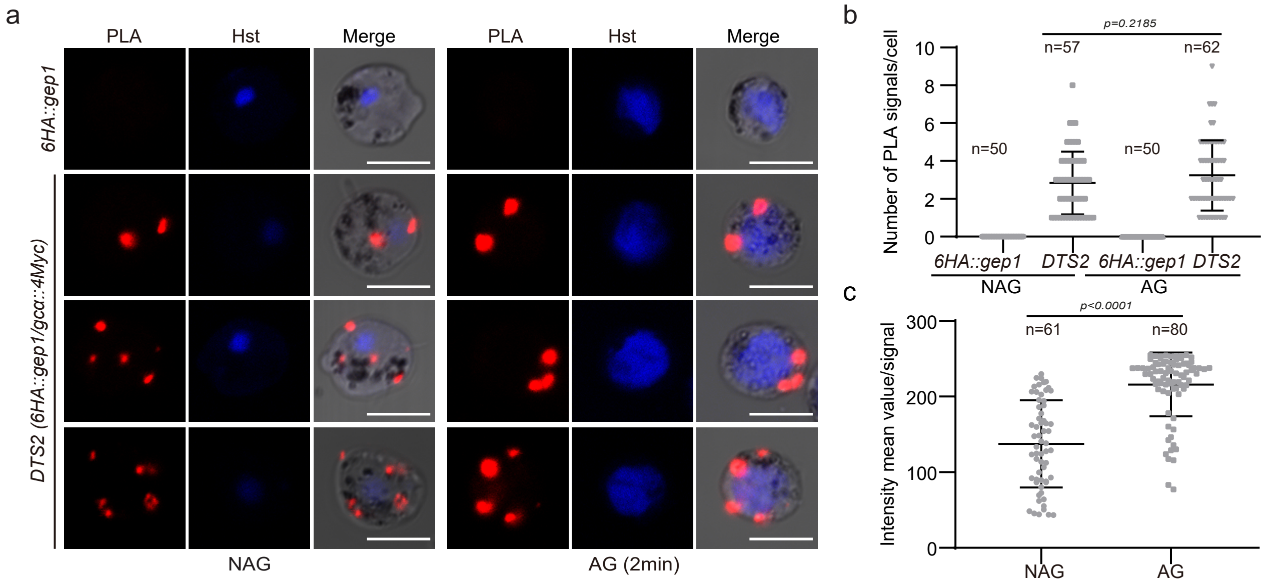
a
PLA
Hst
Merge
PLA
Hst
Merge
6HA::gep1
DTS2 (6HA::gep1/gcα::4Myc)
NAG
AG (2min)
b
p=0.2185
n=57
n=62
n=50
n=50
Number of PLA signals/cell
0
2
4
6
8
10
6HA::gep1
DTS2
6HA::gep1
DTS2
NAG
AG
c
p<0.0001
n=61
n=80
Intensity mean value/signal
0
100
200
300
NAG
AG

Supplementary Fig. 10. XA stimulation likely enhances GEP1/GCα interaction.

a, Proximity Ligation Assay (PLA) detecting protein interaction between GEP1 and GCα in *DTS2* gametocytes. NAG: non-activated, AG: 2 min after XA stimulation. Activated male gametocytes were observed with enlarged nucleus. Scale bar = 5 μm. **b**, Number of PLA signal dot in each cell shown in **a**, n is the number of cells counted. **c**, Fluorescence intensity value for each PLA signal dot shown in **a**. n is the number of PLA signal dot measured. Experiment was repeated three times independently with similar results. Data are represented as mean ± SD; two-tailed unpaired Student's t test in **b** and **c**.

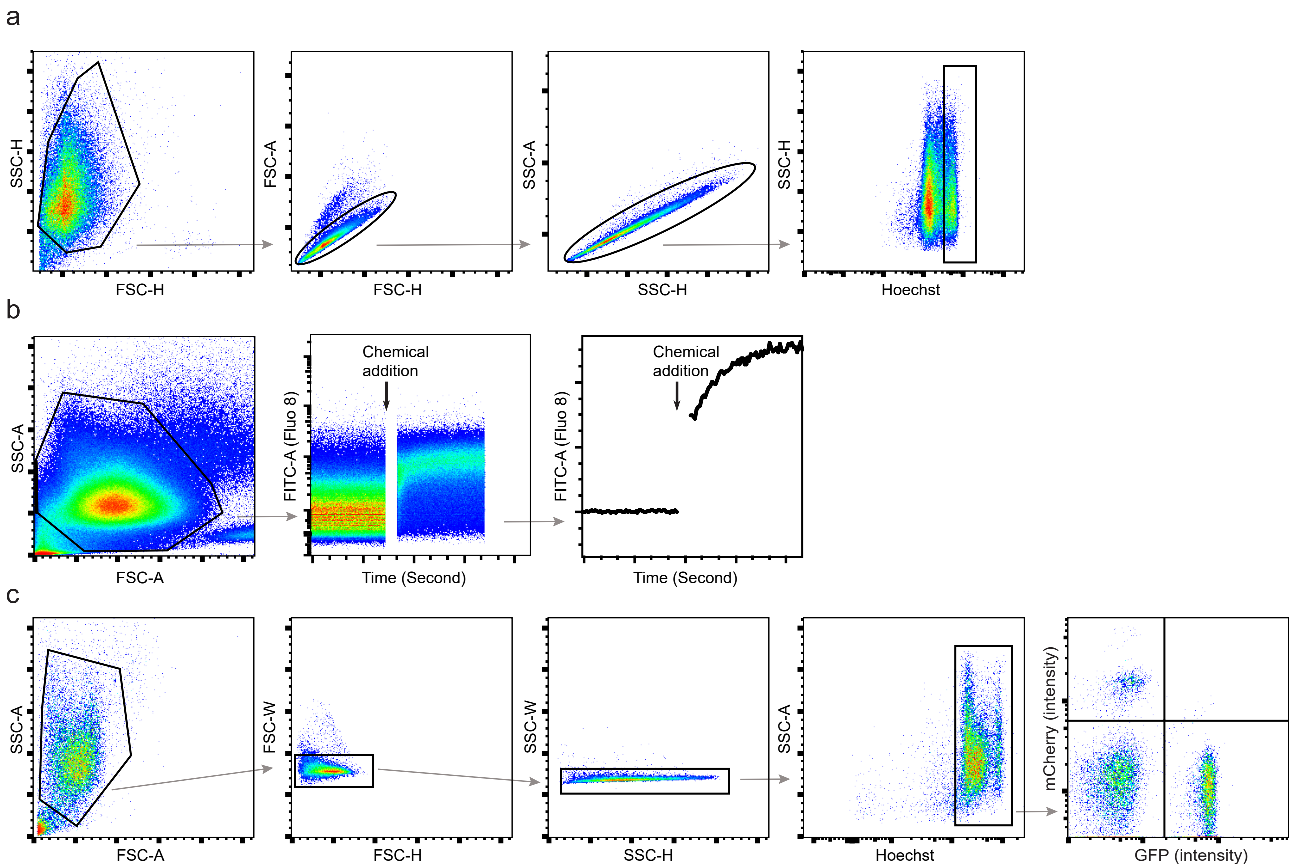

a
SSC-H
FSC-H
FSC-A
FSC-H
SSC-A
SSC-H
SSC-H
Hoechst
b
SSC-A
FSC-A
FITC-A (Fluo 8)
Chemical addition
Time (Second)
FITC-A (Fluo 8)
Chemical addition
Time (Second)
c
SSC-A
FSC-A
FSC-W
FSC-H
SSC-W
SSC-H
SSC-A
Hoechst
mCherry (intensity)
GFP (intensity)

Supplementary Fig. 11. Gating strategies used for cell sorting in flow cytometry. **a**, Gating strategy to sort activated male gametocytes with rounds of genome duplication from the purified gametocytes (including male and female) pre-stained with Hoechst 33342 presented on Figure. 3b. **b**, Gating strategy to sort gametocyte preloaded with Fluo-8 presented on Figure. 3d and Supplementary Figure. 7b. Black arrows indicate the time for A23187, XA or DMSO addition. Signals were collected at 30 sec before until 90 sec post addition. **c**, Gating strategy to sort male (GFP+) and female (mCherry+) gametocytes from Hoechst 33342 pre-stained parasites containing gametocytes and asexual blood stage parasites presented on Supplementary Figure. 6g. Infected mice were phenylhydrazine treated for inducing gametocytes and sulfadiazine treated to reduce asexual blood stage parasites. Parasites were further purified via nycodenz centrifugation before flow cytometry analysis.

Supplementary Table 1. List of candidate gene for screening in this study.

Gene ID	Description	Ortholog in *P.falciparum*	Ortholog in *P.beighei*	Number of TM predicted	Blood-stage phenotype in *P.berghei* from *PlasmoGEM* database	Blood-stage phenotype in this study
PY17X_1243400	7-helix-1 protein, putative	PF3D7_0525400	PBANKA_1240200	3	Dispensable	Dispensable
PY17X_1431500	integral membrane protein GPR180, putative	PF3D7_1213500	PBANKA_1429300	7	Dispensable	Dispensable
PY17X_0918700	serpentine receptor, putative	PF3D7_1131100	PBANKA_0917100	7	Slow	Dispensable
PY17X_1433900	serpentine receptor, putative	PF3D7_1215900	PBANKA_1431600	8	Not tested	Dispensable
PY17X_0524600	serpentine receptor, putative	PF3D7_0422800	PBANKA_0523200	8	Dispensable	Dispensable
PY17X_1421700	GPCR-like receptor SR25, putative	PF3D7_0713400	PBANKA_1420000	8	Essential	Essential
PY17X_0605800	sexual stage-specific protein G37, putative	PF3D7_1204400	PBANKA_0603300	7	Not tested	Dispensable
PY17X_0617400	conserved Plasmodium membrane protein, unknown function	PF3D7_0717000	PBANKA_0614700	7	Not tested	Dispensable
PY17X_0914700	conserved Plasmodium membrane protein, unknown function	PF3D7_1135300	PBANKA_0913200	7	Essential	Essential
PY17X_1128600	protease, putative	PF3D7_0628400	PBANKA_1127100	8	Dispensable	Dispensable
PY17X_1313900	conserved Plasmodium protein, unknown function	PF3D7_1446300	PBANKA_1310000	8	Not tested	Dispensable
PY17X_0702400	folate transporter 1, putative	PF3D7_0828600	PBANKA_0702100	12	Dispensable	Dispensable
PY17X_0933500	folate transporter 2, putative	PF3D7_1116500	PBANKA_0931500	11	Dispensable	Dispensable
PY17X_0309400	GDP-fructose; GMP antiporter, putative	PF3D7_0212000	PBANKA_0308800	8	Not tested	Dispensable
PY17X_0936300	UDP-galactose transporter, putative	PF3D7_1113300	PBANKA_0934300	8	Essential	Dispensable
PY17X_1436400	phosphate translocator, putative	PF3D7_1218400	PBANKA_1434000	10	Dispensable	Dispensable
PY17X_0823700	major facilitator superfamily domain-containing protein, putative	PF3D7_0919500	PBANKA_0820400	12	Dispensable	Dispensable
PY17X_0820300	major facilitator superfamily domain-containing protein, putative	PF3D7_0916000	PBANKA_0817000	11	Dispensable	Dispensable
PY17X_1116300	amino acid transporter, putative	PF3D7_0515500	PBANKA_1115100	14	Dispensable	Dispensable
PY17X_0307300	transporter, putative	PF3D7_0209600	PBANKA_0306700	13	Essential	Essential
PY17X_0917400	amino acid transporter	PF3D7_1132500	PBANKA_0915900	15	Not tested	Dispensable
PY17X_0609400	amino acid transporter, putative	PF3D7_1208400	PBANKA_0606900	10	Dispensable	Dispensable
PY17X_1448600	amino acid transporter, putative	PF3D7_1231400	PBANKA_1446100	10	Essential	Essential
PY17X_1241000	multidrug resistance protein 1, putative	PF3D7_0523000	PBANKA_1237800	11	Essential	Essential
PY17X_1446300	multidrug resistance-associated protein 2, putative	PF3D7_1229100	PBANKA_1443800	11	Slow	Dispensable
PY17X_1315500	multidrug resistance protein 2, putative	PF3D7_1447900	PBANKA_1311700	10	Slow	Dispensable
PY17X_0904900	ABC transporter B family member 3, putative	PF3D7_1145500	PBANKA_0903500	6	Not tested	Dispensable
PY17X_0403400	ABC transporter B family member 4, putative	PF3D7_0302600	PBANKA_0401200	3	Dispensable	Dispensable
PY17X_1358700	ABC transporter B family member 5, putative	PF3D7_1339900	PBANKA_1353300	4	Slow	Dispensable
PY17X_0928600	protein GCN20, putative	PF3D7_1121700	PBANKA_0926600	0	Dispensable	Dispensable
PY17X_1370600	ABC transporter B family member 6, putative	PF3D7_1352100	PBANKA_1364800	4	Essential	Essential
PY17X_0610800	ABC transporter B family member 7, putative	PF3D7_1209900	PBANKA_0608300	6	Not tested	Dispensable
PY17X_1145400	ABC transporter E family member 1, putative	PF3D7_1368200	PBANKA_1144100	0	Essential	Essential
PY17X_1019600	ABC transporter G family member 2, putative	PF3D7_1426600	PBANKA_1018000	5	Slow	Dispensable
PY17X_1425800	ABC transporter F family member 1, putative	PF3D7_0813700	PBANKA_1423800	0	Essential	Essential
PY17X_1222000	ABC transporter I family member 1, putative	PF3D7_0319700	PBANKA_1218800	13	Essential	Essential
PY17X_1031600	FeS assembly ATPase SufC, putative	PF3D7_1413500	PBANKA_1029200	1	Essential	Dispensable
PY17X_0407300	ER membrane protein complex subunit 5, putative	PF3D7_0306700	PBANKA_0405100	2	Essential	Essential
PY17X_0929900	CorA-like Mg2+ transporter protein, putative	PF3D7_1120300	PBANKA_0927900	2	Not tested	Dispensable
PY17X_1018500	CorA-like Mg2+ transporter protein, putative	PF3D7_1427600	PBANKA_1017000	2	Dispensable	Dispensable
PY17X_0703300	magnesium transporter, putative	PF3D7_0827700	PBANKA_0703000	9	Not tested	Dispensable
PY17X_1240600	inner membrane complex protein, putative	PF3D7_0522600	PBANKA_1237300	8	Dispensable	Dispensable
PY17X_1441100	vacuolar iron transporter, putative	PF3D7_1223700	PBANKA_1438600	5	Dispensable	Dispensable
PY17X_1367300	E1-E2 ATPase, putative	PF3D7_1348800	PBANKA_1361600	10	Not tested	Dispensable
PY17X_0109300	zinc transporter ZIP1, putative	PF3D7_0609100	PBANKA_0107700	8	slow	Essential
PY17X_1424200	cation diffusion facilitator family protein, putative	PF3D7_0715900	PBANKA_1422200	4	Dispensable	Dispensable
PY17X_1105200	MOLO1 domain-containing protein, putative	PF3D7_0504500	PBANKA_1104100	2	Dispensable	Dispensable
PY17X_1315200	conserved protein, unknown function	PF3D7_1447600	PBANKA_1311400	1	Dispensable	Dispensable
PY17X_1339400	transmembrane protein 43, putative	PF3D7_1471500	PBANKA_1334700	4	Dispensable	Dispensable
PY17X_1342800	conserved protein, unknown function	PF3D7_1322900	PBANKA_1338100	2	Dispensable	Dispensable
PY17X_1366100	conserved protein, unknown function	PF3D7_1347600	PBANKA_1360400	1	Dispensable	Dispensable
PY17X_1463300	dipeptidyl aminopeptidase 2, putative	PF3D7_1247800	PBANKA_1460700	1	Dispensable	Dispensable
PY17X_0911700	guanylyl cyclase, putative	PF3D7_1138400	PBANKA_0910300	19	slow	Essential
PY17X_1138200	guanylyl cyclase beta	PF3D7_1360500	PBANKA_1136700	22	slow	Dispensable
PY17X_0619700	LEM3/CDC50 family protein	PF3D7_0719500	PBANKA_0617000	2	Not tested	Dispensable
PY17X_0916600	LEM3/CDC50 family protein, putative	PF3D7_1133300	PBANKA_0915100	2	Dispensable	Dispensable
PY17X_0809500	P-type ATPase, putative	PF3D7_0319000	PBANKA_0806300	10	Dispensable	Dispensable
PY17X_1437200	aminophospholipid-transporting P-ATPase, putative	PF3D7_1219600	PBANKA_1434800	9	Essential	Essential
PY17X_1440800	phospholipid-ansporting ATPase, putative	PF3D7_1223400	PBANKA_1438300	10	slow	Essential

Supplementary Table 2. GEP1 interacted proteins detected by Mass spectrum.

Protein	Probability	Unique peptides	Gene_ID	Description	Protein size/aa
tr|V7PTB0|V7PTB0_9APIC	1	21	PY17X_1347900	conserved Plasmodium protein, unknown function	2308
tr|V7PT05|V7PT05_9APIC	1	19	PY17X_1226000	tyrosine--tRNA ligase, putative	372
**tr|V7PFK1|V7PFK1_9APIC**	1	15	PY17X_0911700	**guanylyl cyclase, putative**	3850
tr|V7PBU4|V7PBU4_9APIC	1	15	PY17X_1109100	conserved protein, unknown function	390
tr|V7PHN7|V7PHN7_9APIC	1	13	PY17X_0404000	HAD superfamily protein, putative	1115
tr|V7PBN0|V7PBN0_9APIC	1	10	PY17X_1114400	deoxyribodipyrimidine photo-lyase, putative	849
tr|V7PNU6|V7PNU6_9APIC	1	10	PY17X_0807500	conserved Plasmodium protein, unknown function	1878
tr|V7PDR5|V7PDR5_9APIC	1	9	PY17X_0922400	conserved Plasmodium protein, unknown function	1961
tr|V7PGJ3|V7PGJ3_9APIC	1	9	PY17X_0706700	conserved Plasmodium protein, unknown function	1108
tr|V7PVM5|V7PVM5_9APIC	1	9	PY17X_1221300	oocyst capsule protein Cap380, putative	3290
tr|V7PV53|V7PV53_9APIC	1	8	PY17X_0113800	myosin E, putative	2403
tr|V7PDP7|V7PDP7_9APIC	1	7	PY17X_0501900	reticulocyte binding protein, putative	2748
tr|V7PGA5|V7PGA5_9APIC	1	6	PY17X_0623600	conserved Plasmodium protein, unknown function	1140
tr|V7PJ59|V7PJ59_9APIC	1	6	PY17X_0520600	conserved Plasmodium protein, unknown function	225
tr|V7PU86|V7PU86_9APIC	1	6	PY17X_1322800	conserved protein, unknown function	800
tr|V7PYL5|V7PYL5_9APIC	1	6	PY17X_0103000	SAC3 domain-containing protein, putative	1203
tr|V7PAS9|V7PAS9_9APIC	1	5	PY17X_0710300	2-oxoglutarate dehydrogenase E1 component, putative	1038
tr|V7PBN8|V7PBN8_9APIC	1	5	PY17X_1370200	inner membrane complex protein 1f, putative	1122
tr|V7PI71|V7PI71_9APIC	1	5	PY17X_0526500	reticulocyte binding protein, putative	2757
tr|V7PN10|V7PN10_9APIC	1	5	PY17X_1016800	rRNA (adenosine-2'-O-)-methyltransferase, putative	2379
tr|V7PNJ6|V7PNJ6_9APIC	1	5	PY17X_1135900	Sas10 domain-containing protein, putative	761
tr|V7PP62|V7PP62_9APIC	1	5	PY17X_0216200	Plasmodium exported protein, unknown function	278
tr|V7PRL1|V7PRL1_9APIC	1	5	PY17X_1223000	Cg2 protein, putative	2439
tr|V7PDA4|V7PDA4_9APIC	1	4	PY17X_1102000	fam-a protein	609
tr|V7PIB6|V7PIB6_9APIC	1	4	PY17X_0411100	eukaryotic translation initiation factor 3 subunit K,	235
tr|V7PMC7|V7PMC7_9APIC	1	4	PY17X_1032900	mini-chromosome maintenance complex-binding protein, putative	910
tr|V7PQH8|V7PQH8_9APIC	1	4	PY17X_1452400	CCR4-NOT transcription complex subunit 4, putative	1408
tr|V7PSJ6|V7PSJ6_9APIC	1	4	PY17X_1430600	glycerol-3-phosphate 1-O-acyltransferase, putative	583
tr|V7PXA8|V7PXA8_9APIC	1	4	PY17X_0103400	conserved Plasmodium protein, unknown function	499
tr|V7PXF5|V7PXF5_9APIC	1	4	PY17X_0106800	conserved Plasmodium protein, unknown function	1921
tr|V7PD97|V7PD97_9APIC	1	4	PY17X_0911300	WD repeat-containing protein, putative	1647
tr|V7PJS9|V7PJS9_9APIC	1	4	PY17X_0417200	formate-nitrite transporter, putative	311
tr|V7PKF4|V7PKF4_9APIC	1	4	PY17X_0419100	nucleoporin NUP221, putative	2003
tr|V7PUE6|V7PUE6_9APIC	1	4	PY17X_0101221	reticulocyte binding protein, putative	2771
tr|V7PBU9|V7PBU9_9APIC	1	3	PY17X_0301500	repetitive organellar protein, putative	1980
tr|V7PDJ6|V7PDJ6_9APIC	1	3	PY17X_0915300	DNA-directed RNA polymerase I subunit RPA2, putative	1470
tr|V7PDS4|V7PDS4_9APIC	1	3	PY17X_0309500	conserved Plasmodium protein, unknown function	987
tr|V7PFR2|V7PFR2_9APIC	1	3	PY17X_0934500	histone-lysine N-methyltransferase SET7, putative	707
tr|V7PI90|V7PI90_9APIC	1	3	PY17X_0419300	copper-transporting ATPase, putative	1981
tr|V7PIU0|V7PIU0_9APIC	1	3	PY17X_0709200	protein transport protein SEC61 subunit beta, putative	79
tr|V7PNL8|V7PNL8_9APIC	1	3	PY17X_0215800	AP2 domain transcription factor AP2-L, putative	1269
tr|V7PP81|V7PP81_9APIC	1	3	PY17X_0832600	conserved Plasmodium protein, unknown function	131
tr|V7PPU3|V7PPU3_9APIC	1	3	PY17X_1135500	U3 small nucleolar RNA-associated protein 21, putative	1246
tr|V7PQK0|V7PQK0_9APIC	1	3	PY17X_0213300	cation transporting ATPase, putative	1682
tr|V7PR31|V7PR31_9APIC	1	3	PY17X_1453700	p25-alpha family protein, putative	152
tr|V7PRL8|V7PRL8_9APIC	1	3	PY17X_1136300	zinc finger protein, putative	571
tr|V7PRR3|V7PRR3_9APIC	1	3	PY17X_1140100	conserved Plasmodium protein, unknown function	1415
tr|V7PS22|V7PS22_9APIC	1	3	PY17X_1238000	EELM2 domain-containing protein, putative	825
tr|V7PS95|V7PS95_9APIC	1	3	PY17X_1233600	ribonuclease, putative	2677
tr|V7PXT9|V7PXT9_9APIC	1	3	PY17X_1340100	histone deacetylase, putative	1640
tr|V7PU48|V7PU48_9APIC	1	3	PY17X_1331200	fam-a protein	387
tr|V7PBE1|V7PBE1_9APIC	1	2	PY17X_1109300	conserved protein, unknown function	230
tr|V7PE52|V7PE52_9APIC	1	2	PY17X_0923400	protein phosphatase, putative	288
tr|V7PEX6|V7PEX6_9APIC	1	2	PY17X_1205600	WD repeat-containing protein, putative	2513
tr|V7PEY8|V7PEY8_9APIC	1	2	PY17X_1105900	actin-like protein, putative	556
tr|V7PFG6|V7PFG6_9APIC	1	2	PY17X_0910000	conserved Plasmodium protein, unknown function	166
tr|V7PFI0|V7PFI0_9APIC	1	2	PY17X_1213100	conserved protein, unknown function	175
tr|V7PG25|V7PG25_9APIC	1	2	PY17X_0944100	conserved Plasmodium protein, unknown function	708
tr|V7PGV0|V7PGV0_9APIC	1	2	PY17X_0912800	CLPTM1 domain-containing protein, putative	644
tr|V7PGX6|V7PGX6_9APIC	1	2	PY17X_1201900	conserved Plasmodium protein, unknown function	932
tr|V7PI44|V7PI44_9APIC	1	2	PY17X_0404900	inner membrane complex protein 1e, putative	512
tr|V7PI48|V7PI48_9APIC	1	2	PY17X_0405400	circumsporozoite (CS) protein	427
tr|V7PIM7|V7PIM7_9APIC	1	2	PY17X_0704400	ubiquitin conjugation factor E4 B, putative	1240
tr|V7PIX7|V7PIX7_9APIC	1	2	PY17X_0607700	DNA-directed RNA polymerase III subunit RPC2,	1414
tr|V7PJT8|V7PJT8_9APIC	1	2	PY17X_0404100	DNA-directed RNA polymerases I, II, and III subunit RPABC2, putative	152
tr|V7PQ08|V7PQ08_9APIC	1	2	PY17X_1437000	formin 2, putative	2823
tr|V7PQ99|V7PQ99_9APIC	1	2	PY17X_1444900	conserved Plasmodium protein, unknown function	814
tr|V7PRJ4|V7PRJ4_9APIC	1	2	PY17X_1464800	protein phosphatase PPM4, putative	734
tr|V7PS26|V7PS26_9APIC	1	2	PY17X_1238300	protein phosphatase PPM9, putative	712
tr|V7PTL0|V7PTL0_9APIC	1	2	PY17X_1341000	GTPase-activating protein, putative	616
tr|V7PU13|V7PU13_9APIC	1	2	PY17X_0700600	fam-a protein	338
tr|V7PUT1|V7PUT1_9APIC	1	2	PY17X_0109900	palmitoyltransferase DHHC2, putative	284
tr|V7PUW8|V7PUW8_9APIC	1	2	PY17X_1456000	HD superfamily phosphohydrolase protein, putative	782
tr|V7PWB5|V7PWB5_9APIC	1	2	PY17X_1240300	18S rRNA (guanine-N(7))-methyltransferase, putative	274
tr|V7PYZ7|V7PYZ7_9APIC	1	2	PY17X_0111500	basal complex transmembrane protein 1, putative	634
tr|V7PBB3|V7PBB3_9APIC	0.9999	3	PY17X_1111900	stearoyl-CoA desaturase, putative	944
tr|V7PI49|V7PI49_9APIC	0.9999	2	PY17X_0416400	zinc finger protein, putative	271
tr|V7PJQ2|V7PJQ2_9APIC	0.9999	2	PY17X_1022100	peptidyl-prolyl cis-trans isomerase, putative	435
tr|V7PPF6|V7PPF6_9APIC	0.9999	2	PY17X_1424900	conserved protein, unknown function	393
tr|V7PR12|V7PR12_9APIC	0.9999	2	PY17X_1417200	U4/U6.U5 tri-snRNP-associated protein 2, putative	640

tr|V7PVK6|V7PVK6_9APIC	0.9998	4	PY17X_1220100	ATP-dependent RNA helicase DDX42, putative	720
tr|V7PKI5|V7PKI5_9APIC	0.9998	4	PY17X_0421700	reticulocyte binding protein, putative	2730
tr|V7PQ59|V7PQ59_9APIC	0.9998	2	PY17X_1429100	protein phosphatase PPM7, putative	305
tr|V7PTE3|V7PTE3_9APIC	0.9998	2	PY17X_1347700	ribose-phosphate pyrophosphokinase, putative	541
tr|V7PAT4|V7PAT4_9APIC	0.9997	3	PY17X_0720800	RNA-binding protein NOB1, putative	491
tr|V7PCA3|V7PCA3_9APIC	0.9997	3	PY17X_0717500	cdc2-related protein kinase 3	1263
tr|V7PQS3|V7PQS3_9APIC	0.9997	3	PY17X_1445600	conserved Plasmodium protein, unknown function	1113
tr|V7PBW3|V7PBW3_9APIC	0.9997	2	PY17X_1107500	transcription factor 25, putative	842
tr|V7PIS2|V7PIS2_9APIC	0.9997	2	PY17X_0707800	RWD domain-containing protein, putative	228
tr|V7PGU6|V7PGU6_9APIC	0.9995	2	PY17X_1215200	conserved Plasmodium protein, unknown function	2140
tr|V7PH62|V7PH62_9APIC	0.9995	2	PY17X_1206500	U3 small nucleolar RNA-associated protein 25, putative	995
tr|V7PWR8|V7PWR8_9APIC	0.9995	2	PY17X_1332700	serine/threonine protein phosphatase UIS2, putative	1339
tr|V7PXB4|V7PXB4_9APIC	0.9995	2	PY17X_0103900	cation/H+ antiporter, putative	440
tr|V7PHN2|V7PHN2_9APIC	0.9994	2	PY17X_0403900	CLP1 P-loop domain-containing protein, putative	1402
tr|V7PF60|V7PF60_9APIC	0.9992	2	PY17X_1204500	conserved protein, unknown function	1075
tr|V7PSZ8|V7PSZ8_9APIC	0.9992	2	PY17X_1354600	conserved oligomeric Golgi complex subunit 3, putative	1329
tr|V7PDY2|V7PDY2_9APIC	0.9991	2	PY17X_0916800	conserved Plasmodium protein, unknown function	1073
tr|V7PJX8|V7PJX8_9APIC	0.999	2	PY17X_1015000	large subunit GTPase 1, putative	763
tr|V7PHA3|V7PHA3_9APIC	0.9989	2	PY17X_0511900	conserved Plasmodium protein, unknown function	1500
tr|V7PR35|V7PR35_9APIC	0.9989	2	PY17X_0503500	serine/threonine protein phosphatase 8, putative	2124
tr|V7PVS2|V7PVS2_9APIC	0.9988	2	PY17X_1356100	1-deoxy-D-xylulose 5-phosphate synthase, putative	1047
tr|V7PSE4|V7PSE4_9APIC	0.9987	2	PY17X_1461300	conserved Plasmodium protein, unknown function	511
tr|V7PT67|V7PT67_9APIC	0.9987	2	PY17X_1351300	conserved Plasmodium protein, unknown function	492
tr|V7PMY2|V7PMY2_9APIC	0.9986	2	PY17X_1133200	histone-lysine N-methyltransferase, putative	511
tr|V7PPG6|V7PPG6_9APIC	0.9986	2	PY17X_0211200	conserved protein, unknown function	713
tr|V7PFE7|V7PFE7_9APIC	0.9985	2	PY17X_1209400	histone deacetylase 2, putative	2032
tr|V7PGT8|V7PGT8_9APIC	0.9983	2	PY17X_0706000	conserved protein, unknown function	1006
tr|V7PCP9|V7PCP9_9APIC	0.9982	2	PY17X_0312800	conserved Plasmodium protein, unknown function	354
tr|V7PVZ3|V7PVZ3_9APIC	0.9981	2	PY17X_1231400	periodic tryptophan protein 2, putative	1040
tr|V7PCA7|V7PCA7_9APIC	0.998	1	PY17X_0303800	DNA-directed RNA polymerase II 16 kDa subunit,	133
tr|V7PCB5|V7PCB5_9APIC	0.998	1	PY17X_0718600	histone acetyltransferase, putative	1028
tr|V7PDC0|V7PDC0_9APIC	0.998	1	PY17X_1120400	ATP synthase-associated protein, putative	128
tr|V7PDN8|V7PDN8_9APIC	0.998	1	PY17X_1109600	conserved Plasmodium protein, unknown function	2719
tr|V7PDY6|V7PDY6_9APIC	0.998	1	PY17X_0715000	ribosome assembly protein RRB1, putative	441
tr|V7PEH6|V7PEH6_9APIC	0.998	1	PY17X_0933900	serine esterase, putative	1470
tr|V7PEZ8|V7PEZ8_9APIC	0.998	1	PY17X_1105400	centrosomal protein CEP120, putative	525
tr|V7PFL4|V7PFL4_9APIC	0.998	1	PY17X_0613900	pantothenate kinase 2	677
tr|V7PH11|V7PH11_9APIC	0.998	1	PY17X_0610000	mago-binding protein, putative	150
tr|V7PKB7|V7PKB7_9APIC	0.998	1	PY17X_1038600	RNA-binding protein 8A, putative	107
tr|V7PL90|V7PL90_9APIC	0.998	1	PY17X_0815500	conserved protein, unknown function	856
tr|V7PMQ8|V7PMQ8_9APIC	0.998	1	PY17X_0204600	bromodomain protein, putative	1337
tr|V7PNQ7|V7PNQ7_9APIC	0.998	1	PY17X_1402800	ATP-dependent RNA helicase DHR1, putative	1557
tr|V7PP89|V7PP89_9APIC	0.998	1	PY17X_0214900	ribosome biogenesis protein BRX1, putative	426
tr|V7PPX4|V7PPX4_9APIC	0.998	1	PY17X_1434600	cell traversal protein for ookinetes and sporozoites	185
tr|V7PPX5|V7PPX5_9APIC	0.998	1	PY17X_1138200	guanylyl cyclase beta	3015
tr|V7PPZ0|V7PPZ0_9APIC	0.998	1	PY17X_0811500	E3 ubiquitin-protein ligase, putative	548
tr|V7PQA0|V7PQA0_9APIC	0.998	1	PY17X_1146600	conserved Plasmodium protein, unknown function	2674
tr|V7PQU3|V7PQU3_9APIC	0.998	1	PY17X_1447700	WD repeat-containing protein, putative	434
tr|V7PR30|V7PR30_9APIC	0.998	1	PY17X_0503800	pre-mRNA-splicing factor RDS3, putative	111
tr|V7PRG6|V7PRG6_9APIC	0.998	1	PY17X_1219600	mRNA-capping enzyme subunit beta, putative	613
tr|V7PRY1|V7PRY1_9APIC	0.998	1	PY17X_1233800	zinc finger protein, putative	2333
tr|V7PSC4|V7PSC4_9APIC	0.998	1	PY17X_1237800	zinc finger protein, putative	645
tr|V7PSC6|V7PSC6_9APIC	0.998	1	PY17X_1369000	conserved Plasmodium protein, unknown function	864
tr|V7PSF4|V7PSF4_9APIC	0.998	1	PY17X_1243200	structural maintenance of chromosomes protein 6, putative	1606
tr|V7PT91|V7PT91_9APIC	0.998	1	PY17X_1450800	homocysteine S-methyltransferase, putative	507
tr|V7PTT1|V7PTT1_9APIC	0.998	1	PY17X_1462800	conserved protein, unknown function	178
tr|V7PUX3|V7PUX3_9APIC	0.998	1	PY17X_1456500	DNA gyrase subunit B, putative	930
tr|V7PV18|V7PV18_9APIC	0.998	1	PY17X_1243000	zinc finger protein, putative	674
tr|V7PVD4|V7PVD4_9APIC	0.998	1	PY17X_1368000	conserved Plasmodium protein, unknown function	2660
tr|V7PX98|V7PX98_9APIC	0.998	1	PY17X_0102600	MYND-type zinc finger protein, putative	262
tr|V7PEP9|V7PEP9_9APIC	0.9979	3	PY17X_0939500	conserved Plasmodium protein, unknown function	691
tr|V7P9G4|V7P9G4_9APIC	0.9979	1	PY17X_1320000	syntaxin-6, putative	212
tr|V7PBF4|V7PBF4_9APIC	0.9979	1	PY17X_1308100	zinc finger protein, putative	1223
tr|V7PBN9|V7PBN9_9APIC	0.9979	1	PY17X_1113900	conserved protein, unknown function	251
tr|V7PC70|V7PC70_9APIC	0.9979	1	PY17X_1304100	tubulin epsilon chain, putative	500
tr|V7PCT4|V7PCT4_9APIC	0.9979	1	PY17X_0315400	SUZ domain-containing protein, putative	830
tr|V7PCW5|V7PCW5_9APIC	0.9979	1	PY17X_1319100	LCCL domain-containing protein, putative	879
tr|V7PFV2|V7PFV2_9APIC	0.9979	1	PY17X_0938000	endonuclease/exonuclease/phosphatase family protein, putative	653
tr|V7PGI1|V7PGI1_9APIC	0.9979	1	PY17X_0705600	SAYSvFN domain-containing protein, putative	151
tr|V7PNG3|V7PNG3_9APIC	0.9979	1	PY17X_1133600	splicing factor 3B subunit 5, putative	85
tr|V7PNH1|V7PNH1_9APIC	0.9979	1	PY17X_1005300	gametocyte-specific protein, putative	177
tr|V7PPK0|V7PPK0_9APIC	0.9979	1	PY17X_1412100	M1-family alanyl aminopeptidase, putative	1064
tr|V7PQ92|V7PQ92_9APIC	0.9979	1	PY17X_1129800	amino acid transporter AAT1, putative	626
tr|V7PQP2|V7PQP2_9APIC	0.9979	1	PY17X_1442900	small subunit rRNA processing factor, putative	418
tr|V7PRI3|V7PRI3_9APIC	0.9979	1	PY17X_1463700	pre-mRNA-splicing factor RBM22, putative	393
tr|V7PTE5|V7PTE5_9APIC	0.9979	1	PY17X_1454700	periodic tryptophan protein 1, putative	528
tr|V7PUE5|V7PUE5_9APIC	0.9979	1	PY17X_1225800	AP-3 complex subunit delta, putative	1436
tr|V7PH97|V7PH97_9APIC	0.9978	1	PY17X_0616200	conserved Plasmodium protein, unknown function	367
tr|V7PLF5|V7PLF5_9APIC	0.9978	1	PY17X_0822900	conserved Plasmodium protein, unknown function	585
tr|V7PNE8|V7PNE8_9APIC	0.9978	1	PY17X_1132200	phosphoinositide phosphatase SAC1, putative	814
tr|V7PRS5|V7PRS5_9APIC	0.9978	1	PY17X_1229400	methionine aminopeptidase 1c, putative	680
tr|V7PSD6|V7PSD6_9APIC	0.9978	1	PY17X_1459600	WD repeat-containing protein 82, putative	374
tr|V7PLQ9|V7PLQ9_9APIC	0.9977	2	PY17X_1004000	6-cysteine protein	416
tr|V7PM67|V7PM67_9APIC	0.9977	2	PY17X_0837300	conserved Plasmodium protein, unknown function	743
tr|V7PQ77|V7PQ77_9APIC	0.9977	2	PY17X_1443000	conserved Plasmodium protein, unknown function	782

tr|V7PE13|V7PE13_9APIC	0.9977	1	PY17X_0316800	Plasmodium exported protein, unknown function	248
tr|V7PGY6|V7PGY6_9APIC	0.9977	1	PY17X_0521200	conserved Plasmodium protein, unknown function	710
tr|V7PNC6|V7PNC6_9APIC	0.9977	1	PY17X_0805100	phosphopantetheine adenylyltransferase	1270
tr|V7PNR0|V7PNR0_9APIC	0.9977	1	PY17X_0811600	conserved Plasmodium protein, unknown function	113
tr|V7PPD4|V7PPD4_9APIC	0.9977	1	PY17X_0214200	alpha/beta hydrolase, putative	561
tr|V7PQT0|V7PQT0_9APIC	0.9977	1	PY17X_1457700	ATP-dependent RNA helicase DBP9, putative	867
tr|V7PWV2|V7PWV2_9APIC	0.9977	1	PY17X_1365100	conserved Plasmodium protein, unknown function	562
tr|V7P9J2|V7P9J2_9APIC	0.9976	1	PY17X_1317600	signal recognition particle subunit SRP54, putative	500
tr|V7PFN1|V7PFN1_9APIC	0.9976	1	PY17X_0931900	heat shock protein 90, putative	852
tr|V7PFS2|V7PFS2_9APIC	0.9976	1	PY17X_0914700	conserved Plasmodium membrane protein, unknown function	416
tr|V7PKJ1|V7PKJ1_9APIC	0.9976	1	PY17X_1033700	rhomboid protease ROM8, putative	637
tr|V7PKU7|V7PKU7_9APIC	0.9976	1	PY17X_1027500	conserved Plasmodium protein, unknown function	4192
tr|V7PNX9|V7PNX9_9APIC	0.9976	1	PY17X_1409500	protein phosphatase PPM6, putative	658
tr|V7PQJ8|V7PQJ8_9APIC	0.9976	1	PY17X_1137900	ER membrane protein complex subunit 3, putative	256
tr|V7PBS2|V7PBS2_9APIC	0.9975	1	PY17X_1317500	serine/threonine protein kinase, putative	375
tr|V7PJE2|V7PJE2_9APIC	0.9975	1	PY17X_1028600	NADP-specific glutamate dehydrogenase, putative	470
tr|V7PQY0|V7PQY0_9APIC	0.9975	1	PY17X_1414800	lipoate-protein ligase 1, putative	411
tr|V7PRQ4|V7PRQ4_9APIC	0.9975	1	PY17X_1436000	thrombospondin-related apical membrane protein	349
tr|V7PBZ0|V7PBZ0_9APIC	0.9974	1	PY17X_1312200	eukaryotic translation initiation factor 2-alpha kinase 1, putative	1496
tr|V7PLV1|V7PLV1_9APIC	0.9974	1	PY17X_0812000	GTPase-activating protein, putative	891
tr|V7PLV8|V7PLV8_9APIC	0.9974	1	PY17X_0215700	AP-4 complex subunit beta, putative	892
tr|V7PNS6|V7PNS6_9APIC	0.9974	1	PY17X_0809800	cleavage and polyadenylation specificity factor, putative	954
tr|V7PQ90|V7PQ90_9APIC	0.9974	1	PY17X_1145600	ribosomal protein L1, putative	312
tr|V7PS98|V7PS98_9APIC	0.9974	1	PY17X_1234000	conserved oligomeric Golgi complex subunit 4, putative	1058
tr|V7PTX9|V7PTX9_9APIC	0.9973	1	PY17X_1466200	transcription initiation factor IIA subunit 2, putative	177
tr|V7PU03|V7PU03_9APIC	0.9973	1	PY17X_1330800	DNA-directed RNA polymerase III subunit RPC4,	339
tr|V7PU30|V7PU30_9APIC	0.9973	1	PY17X_1328100	inner membrane complex sub-compartment protein 3, putative	150
tr|V7PNX8|V7PNX8_9APIC	0.9972	2	PY17X_0804500	conserved Plasmodium protein, unknown function	3003
tr|V7PDB9|V7PDB9_9APIC	0.9972	1	PY17X_0902700	membrane associated erythrocyte binding-like protein	1701
tr|V7PI34|V7PI34_9APIC	0.9972	1	PY17X_0414600	vesicle transport v-SNARE protein, putative	197
tr|V7PUK6|V7PUK6_9APIC	0.9972	1	PY17X_0102000	reticulocyte binding protein, putative	611
tr|V7PLN3|V7PLN3_9APIC	0.9971	1	PY17X_1005700	conserved Plasmodium protein, unknown function	855
tr|V7PN97|V7PN97_9APIC	0.9971	1	PY17X_0825400	conserved Plasmodium protein, unknown function	2161
tr|V7PTS5|V7PTS5_9APIC	0.997	2	PY17X_1335900	rab GTPase activator, putative	1581
tr|V7PK32|V7PK32_9APIC	0.997	1	PY17X_0408900	conserved Plasmodium protein, unknown function	280
tr|V7PPE5|V7PPE5_9APIC	0.997	1	PY17X_1423900	GTP-binding translation elongation factor, putative	756
tr|V7PER3|V7PER3_9APIC	0.9969	1	PY17X_1113000	CDK-activating kinase assembly factor MAT1, putative	260
tr|V7PI60|V7PI60_9APIC	0.9969	1	PY17X_0406400	ubiquitin-conjugating enzyme E2, putative	139
tr|V7PPU9|V7PPU9_9APIC	0.9969	1	PY17X_1136000	conserved Plasmodium protein, unknown function	1446
tr|V7PUL6|V7PUL6_9APIC	0.9969	1	PY17X_1232200	nucleolar protein 10, putative	568
tr|V7PGL4|V7PGL4_9APIC	0.9968	1	PY17X_0942000	DNA repair protein RAD51, putative	349
tr|V7PPS8|V7PPS8_9APIC	0.9968	1	PY17X_0815800	conserved Plasmodium protein, unknown function	1219
tr|V7PDK9|V7PDK9_9APIC	0.9966	2	PY17X_1112200	RNA pseudouridylate synthase, putative	9786
tr|V7PS28|V7PS28_9APIC	0.9966	1	PY17X_1224600	protein transport protein USE1, putative	320
tr|V7PMC4|V7PMC4_9APIC	0.9965	2	PY17X_1033400	conserved Plasmodium protein, unknown function	980
tr|V7PT14|V7PT14_9APIC	0.9965	1	PY17X_1356200	exoribonuclease, putative	812
tr|V7PUP0|V7PUP0_9APIC	0.9965	1	PY17X_1233600	ribonuclease, putative	2677
tr|V7PI88|V7PI88_9APIC	0.9964	1	PY17X_0524900	AP-4 complex subunit sigma, putative	146
tr|V7PSK3|V7PSK3_9APIC	0.9963	1	PY17X_1431700	DNA-directed RNA polymerases I, II, and III subunit RPABC3, putative	143
tr|V7PAT9|V7PAT9_9APIC	0.9962	1	PY17X_0711000	Snf2-related CBP activator, putative	1825
tr|V7PGX8|V7PGX8_9APIC	0.9961	1	PY17X_0607900	conserved Plasmodium protein, unknown function	678
tr|V7PPB9|V7PPB9_9APIC	0.996	1	PY17X_1406400	conserved protein, unknown function	512
tr|V7PNF4|V7PNF4_9APIC	0.9959	1	PY17X_1006300	transcription factor, putative	373
tr|V7PR83|V7PR83_9APIC	0.9959	1	PY17X_1457000	polyadenylation factor subunit 2, putative	481
tr|V7PT86|V7PT86_9APIC	0.9959	1	PY17X_1450400	conserved Plasmodium protein, unknown function	1545
tr|V7PK92|V7PK92_9APIC	0.9957	1	PY17X_1006900	AAA family ATPase, putative	625
tr|V7PN06|V7PN06_9APIC	0.9957	1	PY17X_0503400	conserved Plasmodium protein, unknown function	252
tr|V7PGS7|V7PGS7_9APIC	0.9956	1	PY17X_0523200	ribosome-recycling factor, putative	266
tr|V7PS12|V7PS12_9APIC	0.9954	1	PY17X_1222800	cg1 protein, putative	997
tr|V7PSD2|V7PSD2_9APIC	0.9953	1	PY17X_1238900	conserved protein, unknown function	906
tr|V7PHT7|V7PHT7_9APIC	0.995	1	PY17X_0613500	trafficking protein particle complex subunit 5, putative	184
tr|V7PQE1|V7PQE1_9APIC	0.995	1	PY17X_1434400	conserved Plasmodium protein, unknown function	727
tr|V7PWG8|V7PWG8_9APIC	0.995	1	PY17X_0116400	PIR protein	632
tr|V7PF24|V7PF24_9APIC	0.9949	1	PY17X_1209900	protein GPR89, putative	985
tr|V7PHB0|V7PHB0_9APIC	0.9949	1	PY17X_0520000	conserved Plasmodium membrane protein, unknown function	442
tr|V7PMT8|V7PMT8_9APIC	0.9949	1	PY17X_1129700	polyadenylate-binding protein 3, putative	535
tr|V7PD44|V7PD44_9APIC	0.9946	1	PY17X_0604900	major facilitator superfamily domain-containing protein, putative	1040
tr|V7PRY5|V7PRY5_9APIC	0.9941	1	PY17X_1220200	peptidase, putative	873
tr|V7PAC6|V7PAC6_9APIC	0.9939	1	PY17X_1306300	conserved protein, unknown function	298
tr|V7PDF5|V7PDF5_9APIC	0.9939	1	PY17X_0905900	SUMO-activating enzyme subunit 1, putative	369
tr|V7PHN1|V7PHN1_9APIC	0.9939	1	PY17X_0511200	conserved protein, unknown function	1140
tr|V7PJI1|V7PJI1_9APIC	0.9939	1	PY17X_0409600	dual specificity protein phosphatase, putative	471
tr|V7PKU5|V7PKU5_9APIC	0.9939	1	PY17X_0829200	conserved Plasmodium protein, unknown function	362
tr|V7PB62|V7PB62_9APIC	0.9938	1	PY17X_1116200	conserved protein, unknown function	779
tr|V7PF12|V7PF12_9APIC	0.9938	1	PY17X_1104300	protein kinase, putative	684
tr|V7PJV1|V7PJV1_9APIC	0.9938	1	PY17X_1017300	conserved Plasmodium protein, unknown function	1079
tr|V7PTX5|V7PTX5_9APIC	0.9937	1	PY17X_1332300	zinc finger protein, putative	349
tr|V7PF90|V7PF90_9APIC	0.9936	1	PY17X_1215600	GDP-L-fucose synthase, putative	343
tr|V7PJR6|V7PJR6_9APIC	0.9936	1	PY17X_1020400	conserved Plasmodium protein, unknown function	365
tr|V7PIZ6|V7PIZ6_9APIC	0.9926	1	PY17X_1031200	DnaJ protein, putative	381
tr|V7PKQ1|V7PKQ1_9APIC	0.9926	1	PY17X_1029700	conserved Plasmodium protein, unknown function	1194

tr|V7PMY3|V7PMY3_9APIC	0.9926	1	PY17X_1018300	exosome complex component RRP41, putative	246
tr|V7PRM7|V7PRM7_9APIC	0.9926	1	PY17X_1224800	cell division cycle ATPase, putative	1162
tr|V7PDF8|V7PDF8_9APIC	0.9925	1	PY17X_1117000	conserved Plasmodium protein, unknown function	198
tr|V7PV90|V7PV90_9APIC	0.9925	1	PY17X_1248600	conserved Plasmodium protein, unknown function	96
tr|V7PIV8|V7PIV8_9APIC	0.9921	1	PY17X_1215900	SPRY domain-containing protein, putative	2029
tr|V7PLN0|V7PLN0_9APIC	0.9916	1	PY17X_0803600	conserved Plasmodium protein, unknown function	1255
tr|V7PSS8|V7PSS8_9APIC	0.9915	2	PY17X_1220000	conserved Plasmodium protein, unknown function	1751
tr|V7PK17|V7PK17_9APIC	0.9914	2	PY17X_1002800	pre-mRNA-splicing factor CLF1, putative	703
tr|V7PI27|V7PI27_9APIC	0.9914	1	PY17X_0621000	Ham1-like protein, putative	196
tr|V7PFL6|V7PFL6_9APIC	0.9908	1	PY17X_1215500	translation initiation factor eIF-2B subunit delta, putative	1039
tr|V7PFS5|V7PFS5_9APIC	0.9908	1	PY17X_0935800	conserved Plasmodium protein, unknown function	700
tr|V7PFZ3|V7PFZ3_9APIC	0.9904	1	PY17X_0624200	Rab GTPase activator and protein kinase, putative	1634
tr|V7PQQ3|V7PQQ3_9APIC	0.9903	1	PY17X_1456800	vacuolar protein sorting-associated protein 16, putative	965
tr|V7PQT1|V7PQT1_9APIC	0.9902	1	PY17X_1446300	multidrug resistance-associated protein 2, putative	1975
tr|V7PTZ1|V7PTZ1_9APIC	0.99	1	PY17X_1430200	asparagine-rich antigen, putative	993
tr|V7PGT1|V7PGT1_9APIC	0.9897	2	PY17X_0911400	conserved Plasmodium protein, unknown function	1744
tr|V7PIW3|V7PIW3_9APIC	0.9892	1	PY17X_0510300	EF-hand calcium-binding domain-containing protein, putative	908
tr|V7PNS0|V7PNS0_9APIC	0.9891	1	PY17X_0810300	stomatin-like protein, putative	398
tr|V7PVP2|V7PVP2_9APIC	0.9891	1	PY17X_1358700	ABC transporter B family member 5, putative	820
tr|V7PKR9|V7PKR9_9APIC	0.9888	1	PY17X_1028500	conserved Plasmodium protein, unknown function	2793
tr|V7PNZ8|V7PNZ8_9APIC	0.9888	1	PY17X_1411000	RNA-binding protein, putative	154
tr|V7PED3|V7PED3_9APIC	0.9882	1	PY17X_0942800	DEAD/DEAH box helicase, putative	1109
tr|V7PNU2|V7PNU2_9APIC	0.9878	1	PY17X_0807900	mediator of RNA polymerase II transcription subunit 10, putative	257
tr|V7PMM4|V7PMM4_9APIC	0.9876	1	PY17X_0827200	DNA-directed RNA polymerase II subunit RPB3, putative	335
tr|V7PWC1|V7PWC1_9APIC	0.9873	2	PY17X_1240600	inner membrane complex protein, putative	486
tr|V7PI62|V7PI62_9APIC	0.9873	1	PY17X_0416900	inorganic pyrophosphatase, putative	367
tr|V7PUT0|V7PUT0_9APIC	0.9872	1	PY17X_1345400	mitochondrial fission 1 protein, putative	141
tr|V7PKZ0|V7PKZ0_9APIC	0.9869	2	PY17X_0834700	peptidyl-prolyl cis-trans isomerase, putative	590
tr|V7PD57|V7PD57_9APIC	0.9868	1	PY17X_1105200	MOLO1 domain-containing protein, putative	275
tr|V7PU62|V7PU62_9APIC	0.9853	2	PY17X_1324900	conserved Plasmodium protein, unknown function	2979
tr|V7PMY5|V7PMY5_9APIC	0.9847	1	PY17X_0833100	transcription initiation factor TFIID subunit 7, putative	389
tr|V7PRP3|V7PRP3_9APIC	0.9845	1	PY17X_1138500	protein arginine N-methyltransferase 5, putative	729
tr|V7PLY2|V7PLY2_9APIC	0.9832	1	PY17X_1011200	conserved protein, unknown function	374
tr|V7PHJ7|V7PHJ7_9APIC	0.9823	2	PY17X_0607100	conserved protein, unknown function	1196
tr|V7PJQ3|V7PJQ3_9APIC	0.9809	1	PY17X_1012000	conserved protein, unknown function	277
tr|V7PFH9|V7PFH9_9APIC	0.9808	1	PY17X_0910600	conserved Plasmodium protein, unknown function	2069
tr|V7PMS4|V7PMS4_9APIC	0.9806	1	PY17X_1128400	eukaryotic translation initiation factor 2-alpha kinase, putative	2580
tr|V7PXK9|V7PXK9_9APIC	0.9775	1	PY17X_0109300	zinc transporter ZIP1, putative	344
tr|V7PWK6|V7PWK6_9APIC	0.9761	1	PY17X_1248100	phosphoenolpyruvate/phosphate translocator, putative	518
tr|V7PDX1|V7PDX1_9APIC	0.9756	1	PY17X_0927200	calcium-dependent protein kinase 7	1913
tr|V7PRZ4|V7PRZ4_9APIC	0.9745	1	PY17X_1235000	AP2 domain transcription factor AP2-O2	2129
tr|V7PBN5|V7PBN5_9APIC	0.9743	1	PY17X_1102800	conserved protein, unknown function	381
tr|V7PFR0|V7PFR0_9APIC	0.9688	2	PY17X_0609100	conserved Plasmodium protein, unknown function	4285
tr|V7PX28|V7PX28_9APIC	0.967	1	PY17X_1358100	conserved Plasmodium protein, unknown function	271
tr|V7PEF2|V7PEF2_9APIC	0.9652	1	PY17X_0306900	exosome complex component MTR3, putative	271
tr|V7PG51|V7PG51_9APIC	0.9651	1	PY17X_0926000	conserved protein, unknown function	492
tr|V7PHH0|V7PHH0_9APIC	0.9632	2	PY17X_0928100	JmjC domain-containing protein, putative	447
tr|V7PQI2|V7PQI2_9APIC	0.9557	1	PY17X_1403200	methyltransferase, putative	227
tr|V7PTT3|V7PTT3_9APIC	0.9547	1	PY17X_1337600	mediator of RNA polymerase II transcription subunit 6, putative	205
tr|V7PTD9|V7PTD9_9APIC	0.9531	1	PY17X_1415500	conserved protein, unknown function	524
tr|V7PMS3|V7PMS3_9APIC	0.9452	1	PY17X_0823100	gamma-glutamylcysteine synthetase	999
tr|V7PEM6|V7PEM6_9APIC	0.9447	1	PY17X_0312600	conserved Plasmodium protein, unknown function	830
tr|V7PJ48|V7PJ48_9APIC	0.9413	1	PY17X_1035600	DNA damage-inducible protein 1, putative	385
tr|V7PSD9|V7PSD9_9APIC	0.9392	3	PY17X_1240400	conserved Plasmodium protein, unknown function	8949
tr|V7PDX8|V7PDX8_9APIC	0.937	1	PY17X_0313900	autophagy-related protein 11, putative	1423
tr|V7PIS1|V7PIS1_9APIC	0.933	1	PY17X_0513200	methyltransferase, putative	577
tr|V7PJE5|V7PJE5_9APIC	0.9298	1	PY17X_1020200	conserved Plasmodium protein, unknown function	364
tr|V7PZB8|V7PZB8_9APIC	0.921	1	PY17X_1250700	PIR protein	316
tr|V7PE58|V7PE58_9APIC	0.9198	2	PY17X_0935400	GTPase-activating protein, putative	372
tr|V7PAK3|V7PAK3_9APIC	0.9149	1	PY17X_1371500	fam-c protein	94
tr|V7PMS9|V7PMS9_9APIC	0.9094	1	PY17X_1128800	DNA methyltransferase 1-associated protein 1, putative	385
tr|V7PJB0|V7PJB0_9APIC	0.9063	1	PY17X_1031000	zinc finger protein, putative	697

Supplementary Table 3. Primers and oligonucleotides used in this study.

Oligo sequence for candidate genes (except gep1) knockout plasmid construction							
Gene ID	Gene name	Gene size (bp)/ deleted gene size (bp)	Left homologous arm		Right homologous arm		Target site of sgRNA	
			Forward primer	Reverse primer	Forward primer	Reverse primer	Oligo (Forward)	Oligo (Reverse)
PY17X_1243400	7-helix-1 protein, putative	1410 / 756	CGGGGTACCTATATGTGTAACTATTAGGG	CATGCCATGGAGCCACAATGACCTTTTATT	CCGCTCGAGGGAAGTGCACATGGGTATGC	CCCCTTAAGCAGGTCTATCTGGAATGTTA	TATTGCAAATCAATCAATTTATTG	AAACCAATAAATTGATTGATTTGC
PY17X_1431500	integral membrane protein GPR180, putative	2080 / 327	CGGGGTACCTACATTAATGCTTGATATTG	CATGCCATGGCATATTCTATATTGAATCC	CCGCTCGAGCACTAAGCTATGGTATAACA	CCCCTTAAGTAAGCGTATGAAACATGCAC	TATTGTTTATTCACAAAATGGTAT	AAACATACCATTTTGTGAATAAAC
PY17X_0918700	serpentine receptor, putative	1878 / 498	CGGGGTACCTTAATTAGGACCAATGTAA	CATGCCATGGACCTTGACTTGACGTATCC	CCGCTCGAGCTAGTTGGTGATGATAAA	CCCCTTAAGATAAAGACAGACGATAT	TATTGGTAATACTATATCAACATA	AAACTATGTTGATATAGTATTACC
PY17X_1433900	serpentine receptor, putative	1776 / 545	CGGGGTACCTTCATGTATTAACAATGGA	CATGCCATGGATATATATCACGTCTGTG	CCGCTCGAGCAGAACACTCAGATTTGGA	CCCCTTAAGCACACTGATATCCATACTT	TATTGTCATCTTCTTCATAAGAAG	AAACCTTCTTATGAAGAAGATGAC
PY17X_0524600	serpentine receptor, putative	2122 / 434	CGGGGTACCTATATTGTTATGTGTGTACA	CATGCCATGGGATTACACCAATTTGATATG	CCGCTCGAGGATATAGACAATCAATTAGA	CCCCTTAAGGTTTGATGATTCTGTATCTC	TATTGTATCATCAAAAGTAGTATA	AAACTATACTACTTTTGATGATAC
PY17X_1421700	GPCR-like receptor SR25, putative	1347 / 531	CGGGGTACCAGATTACTACGTTGTCGATT	CATGCCATGGAGTTCATCACCACTGTCATC	CCGCTCGAGGCCATCTTATTACAGTATGC	CCCCTTAAGTGACTTTTGATGGTAAAGAC	TATTGAATTTGCTACTATAGTATG	AAACCATACTATAGTAGCAAATTC
PY17X_0605800	sexual stage-specific protein G37, putative	2513 / 459	CGGGGTACCTTAGTAACACCTCAACTTATA	CATGCCATGGGTGGTATATCCTTAAAGACC	CCGCTCGAGGCATGTATCAGTAGGTATAG	CCGGAATTCGTATACCCCATAATGACGAT	TATTGTAAATTTCGCTTCATTGCTA	AAACTAGCAATGAAGCGAAATTTAC
PY17X_0617400	conserved Plasmodium membrane protein, unknown function	2400 / 415	CGGGGTACCCTATAATTCCTTGTAGACGT	CATGCCATGGGATAGCAATTCGTTTAACTA	CCGCTCGAGGTATGTTTACAACTGTTGCA	CCGGAATTCAAAGTTGATACTGGTATCTA	TATTGTTAATTTCGTCTTAGTTAT	AAACATAACTAAGACGAAATTAAC
PY17X_0914700	conserved Plasmodium membrane protein, unknown function	1251 / 343	CGGGGTACCTTACACGTGGCGAATGATAT	CATGCCATGGCGTAGTTCACTAGTAAATGA	CCGCTCGAGGGCTTATTATCAATTATACTC	CCGGAATTCGATGATATAGCTGTACTTATC	TATTGAATATAAAAGAAAGAGTCG	AAACCGACTCTTTCTTTTATATTC
PY17X_1128600	protease, putative	2155 / 385	CGGGGTACCTCCTTCGCTATATGTGCGAT	CATGCCATGGCAAGATATATGAAATTGCCT	CCGCTCGAGCGTTTATCTTTATCTGTTAG	CCGGAATTCGGCAGTTCTGTGTATTCATT	TATTGGTTTTTCTTGTATAGCAAA	AAACTTTGCTATACAAGAAAAACC
PY17X_1313900	conserved Plasmodium protein, unknown function	3636 / 626	CGGGGTACCCGGCATATGTGGATGTTAGC	CATGCCATGGGCCCAAACAACAACAATCTT	CCGCTCGAGAGGGTAGTGAGGAATGTTCAT	CCCCTTAAGCCATTTGTTACTATATATGG	TATTGTTTTGAAAAATATAGAATT	AAACAATTCTATATTTTTCAAAAC
PY17X_0702400	folate transporter 1, putative	1867 / 1102	CCCAAGCTTATCATGTTTCCGTTACTA	CATGCCATGGAGGGTATAATATAATCGA	CCGCTCGAGGTTGTTGAAAAAAGGGAT	CCGGAATTCTGAACTGACCATTACTGC	TATTGATACTTATCAGCTTCATCC	AAACGGATGAAGCTGATAAGTATC
PY17X_0933500	folate transporter 2, putative	1362 / 1404	CCCAAGCTTTGATGTCTTTATGTATTTCG	CATGCCATGGTATTTGTGTTCTTCTTTCTC	CCGCTCGAGATGAGTGGCAAACCCAAAAT	CCGGAATTCTGGGGTTTTCGTCTCTACAA	TATTGGTTTCCGACCTTCTTTTAT	AAACATAAAAGAAGGTCGGAAACC
PY17X_0309400	GDP-fructose; GMP antiporter, putative	1325 / 1325	CCCAAGCTTCCCCACACATACATATCTTT	CATGCCATGGTTGTCTATTTGTTCAGCTTA	CCGCTCGAGATGAACAAGTCAGACAACAC	CCGGAATTCTACCAATTTTGTGAAGAAAC	TATTGTGGTAATTGAAAATAATAC	AAACGTATTATTTTCAATTACCAC
PY17X_0936300	UDP-galactose transporter, putative	1044 / 1044	CCCAAGCTTAATTAACACCTTAACAGC	CATGCCATGGTCCTCACATAGTTATCAA	CCGCTCGAGCCATTTCGTTTCGTACTT	CCGGAATTCAGAAACTGGAGGAGATGT	TATTGAAATGCATCAAACAACATT	AAACAATGTTGTTTGATGCATTTC
PY17X_1436400	phosphate translocator, putative	2650 / 1462	CCCAAGCTTCATACCTAAAGAGGAGAA	CATGCCATGGAAATTAACAGACTGTCTC	CCGCTCGAGTAAGTGCTCTCTGCTCGT	CCGGAATTCGTTATTGTTGTAATCTGA	TATTGATGTTTTTGATACTTAAAA	AAACTTTTAAGTATCAAAAACATC
PY17X_0823700	major facilitator superfamily domain-containing protein, putative	1470 / 1470	CGGGGTACCTTGTTCTCTTTCTCCATCTA	CATGCCATGGATTTTTGTAAGGTACATCAT	CCGCTCGAGTTTCACACTTATTCATTTTG	CCGGAATTCATTGAGTGCGAGAGTAGAAA	TATTGGATATGATATATGCCCAGA	AAACTCTGGGCATATATCATATCC
PY17X_0820300	major facilitator superfamily domain-containing protein, putative	4033 / 1312	CCCAAGCTTCACAAACAGACACATAACAG	CATGCCATGGATCTCTAGAATCAGCTCAAT	CCGCTCGAGGAAGAGAGTAAAACCCCATT	CCGGAATTCATCGAAGATATCATTGACGT	TATTGAAGCTATAAAACAAGCAAA	AAACTTTGCTTGTTTTATAGCTTC
PY17X_0307300	transporter, putative	3986 / 363	CCCAAGCTTAATGAAAGGATAAGAGTGT	CATGCCATGGATTGGGGTAACATGTGCTA	CCGCTCGAGAATCGAACTATACAAGAAG	CCGGAATTCGAATATGTCCATTACCAG	TATTGAGCATATTTTCATTATCTA	AAACTAGATAATGAAAATATGCTC
PY17X_0917400	amino acid transporter	4068 / 444	CCCAAGCTTCTGTCTAATGGTCCCAATAA	CATGCCATGGGAATAACACCTCCCCTCTTT	CCGCTCGAGATTTTGAGTTAGAAGAAG	CCGGAATTCATACATTTCAACAGATGG	TATTGTATCATGTTCATGCTCTTT	AAACAAAGAGCATGAACATGATAC
PY17X_0609400	amino acid transporter, putative	4545 / 370	CCCAAGCTTCGTGCTCATTTTGTGTTC	CATGCCATGGAGCTTCAATTTGTCTGTT	CCGCTCGAGAAACATTCTGGTTGGCTA	CCGGAATTCGACTTCATTTCATTTGCC	TATTGAGACATGTTAAGAGAAAATG	AAACCATTTTCTCTTAACATGTCTC
PY17X_1448600	amino acid transporter, putative	5556 / 388	CCCAAGCTTATTATGAGTAACCTTCGC	CATGCCATGGACTTAAACAAACGCAAAT	CCGCTCGAGGGAAATCTGTTTGTATGC	CCGGAATTCTTTTAATTGGTGTTGGAC	TATTGTTTAATTGATGAAGTAGGA	AAACTCCTACTTCATCAATTAAAC
PY17X_1241000	multidrug resistance protein 1, putative	4266 / 684	CGGGGTACCGAAATCTACCGTTGAGTTGT	CATGCCATGGATTACACTTTCTCCACAAT	CCGCTCGAGTGGGTTCAACTTGCATGG	CCCCTTAAGGTTCAACTATTACACTTCC	TATTGTTGATAGAAATCAAAATAA	AAACTTATTTTGATTTCTATCAAC
PY17X_1446300	multidrug resistance-associated protein 2, putative	5928 / 665	CGGGGTACCCTACATATCCATGATGAATG	CATGCCATGGACACTATGCGTAACTATAT	CCGCTCGAGTCGATATGGAAGTAACTG	CCCCTTAAGGCATACTTGATATGAATTCAC	TATTGATTATTATGAATATAATAG	AAACCTATTATATTCATAATAATC
PY17X_1315500	multidrug resistance protein 2, putative	2877 / 481	CGGGGTACCCACATTAGTTGACATGATTGC	CATGCCATGGGTATATGGCATATCATTACC	CCGCTCGAGGGTTTAGCATCAACTGAAGT	CCCCTTAAGAATTAGATAATGCACCAC	TATTGATTATTATGAATATAATAG	AAACCTATTATATTCATAATAATC
PY17X_0904900	ABC transporter B family member 3, putative	2624 / 755	CGGGGTACCGGAAATCGAAGTTATAGACA	CATGCCATGGCCTTAAAAGACACAACCCT	CCGCTCGAGTAGCACGTCTACACACAACG	CCGGAATTCTCACCTTGGGAAAGTGGATC	TATTGATTATTATGAATATAATAG	AAACCTATTATATTCATAATAATC
PY17X_0403400	ABC transporter B family member 4, putative	3876 / 577	CGGGGTACCGGAAATGCTGAAGATGTAA	CATGCCATGGTTGGCTAAATGTACTAATAT	CCGCTCGAGCACCACATTGTGACATATCC	CCCCTTAAGTGTAACTGTGTGTTGGCTAG	TATTGATTATTATGAATATAATAG	AAACCTATTATATTCATAATAATC
PY17X_1358700	ABC transporter B family member 5, putative	2463 / 679	CGGGGTACCGCTTGGTTTAGGAATTGTTGG	CATGCCATGGCATATAAGGACCTATTCAG	CCGCTCGAGATGTTCATTATCATCTACC	CCCCTTAAGAACACACAATAAATCTGC	TATTGATTATTATGAATATAATAG	AAACCTATTATATTCATAATAATC
PY17X_0928600	protein GCN20, putative	2322 / 991	CGGGGTACCGAAGTATGGGTAATGAGAA	CATGCCATGGCCCCATATATCTATGTGTG	CCGCTCGAGCTTGGTTTTGACTCCAACCTTC	CCCCTTAAGCTCTTAATCTGATAAGCACAC	TATTGATTATTATGAATATAATAG	AAACCTATTATATTCATAATAATC
PY17X_1370600	ABC transporter B family member 6, putative	3087 / 552	CGGGGTACCGGCCTACATGTATAAATGC	CATGCCATGGTACAAGATATGAGAGAGT	CCGCTCGAGCAATGAACATAGTAATGACG	CCCCTTAAGGTAGTAAAGACACTATTCC	TATTGAATCGGCTGCATAATTAAG	AAACCTTAATTATGCAGCCGATTC
PY17X_0610800	ABC transporter B family member 7, putative	2418 / 605	CGGGGTACCGCATATATGGATGTATGGG	CATGCCATGGCTACCTACCTTAACAATCC	CCGCTCGAGATCTATGATATGTCTACTC	CCCCTTAAGGATTCACCATTAAGGAATC	TATTGATTATTATGAATATAATAG	AAACCTATTATATTCATAATAATC
PY17X_1145400	ABC transporter E family member 1, putative	2055 / 690	CGGGGTACCCCGTTTATATCAGTAAACCA	CATGCCATGGCGGTGCTGCTACGATAAATGG	CCGCTCGAGTATGGGGAAAGGCAGGTGCA	CCGGAATTCCGTGTTCGACAACAAAAGCTG	TATTGAATGGCGAGATATATTATC	AAACGATAATATATCTCGCCATTC
PY17X_1019600	ABC transporter G family member 2, putative	1971 / 769	CGGGGTACCCGACATAGACAATGAATGTATAGTG	CATGCCATGGTCTAAGTTTCCATTATCGACTG	CCGCTCGAGGAAGAAGAAGGATATTATATGGA	CCCCTTAAGATTCAGCTAATGCTTGTGACA	TATTGATTATTATGAATATAATAG	AAACCTATTATATTCATAATAATC
PY17X_1425800	ABC transporter F family member 1, putative	3792 / 608	CGGGGTACCTATCATATGGTGTATAAG	CATGCCATGGGCCAACTTTCACACATATC	CCGCTCGAGCAATGGGTGTGGAAAATCG	CCGGAATTCTCACTTCTTTTATCACTTC	TATTGATGATGATAATGATGATTGT	AAACACAATCATCATTATCATCATC
PY17X_1222000	ABC transporter I family member 1, putative	8548 / 520	CGGGGTACCCTTGAAATGAACTCAATGCTC	CATGCCATGGCATACAAGCAAGATCTATG	CCGCTCGAGCGACGCATGCACATATATG	CCCCTTAAGGTCCTATGGAGGTTTGTCGTAG	TATTGCCCAAACGTTTAAACTATT	AAACAATAGTTTAAACGTTTGGGC
PY17X_1031600	FeS assembly ATPase SufC, putative	1083 / 809	CGGGGTACCGCTCACAGATAAGGATTAGTA	CATGCCATGGGATCATCTGTATTAGAATATTCC	CCGCTCGAGGCACAATTTGTTGAGTAGATG	CCCCTTAAGCTCATTGACTATTGGTAGTG	TATTGATTATTATGAATATAATAG	AAACCTATTATATTCATAATAATC
PY17X_0407300	ER membrane protein complex subunit 5, putative	706 / 706	CCCAAGCTTGGGTTGTCACATATTTTAT	CATGCCATGGCCTTTATTTGTATTTCCTC	CCGCTCGAGGTTATCATTGCCCCATTTC	CCGGAATTCGAGTTATTCCTTTAGCATTTTAG	TATTGCAGTTATGATAACCTTGAT	AAACATCAAGGTTATCATAACTGC
PY17X_0929900	CorA-like Mg2+ transporter protein, putative	1466 / 538	CGGGGTACCAATAATACGTGGTTGCTTAC	CATGCCATGGTACGAATATCAACAAACTCA	CCGCTCGAGATAATAACCACAACCCAACA	CCGGAATTCTTTCCAAATCACTACAATCTT	TATTGTTGTTTGGTCTTAAAACATA	AAACTATGTTTTAAGACCAAACAAC
PY17X_1018500	CorA-like Mg2+ transporter protein, putative	1443 / 478	CCCAAGCTTGGAAGATAACTGTGTTCTGA	CATGCCATGGCGAATACAATGGTTTAGTTT	CCGCTCGAGGTTCATAAGATAAGTAACGGAG	CCGGAATTCTATTCGAGTAGCATTTCAGT	TATTGAACGAAAGTGATAGTGATG	AAACCATCACTATCACTTTCGTTC
PY17X_0703300	magnesium transporter, putative	1723 / 999	CCCAAGCTTATTTTTACAACCTTCCAGTC	CATGCCATGGCAACAGTGATGTATAGAGCAG	CCGCTCGAGATGGTAATACTAAAAGGTGC	CCGGAATTCTAGACTACTAAGGAAAACGAT	TATTGCAGTTGTTGCTCCGTTTGT	AAACACAAACGGAGCAACAACTGC
PY17X_1240600	inner membrane complex protein, putative	1461 / 1461	CCCAAGCTTTCCCTATTCAAACCTACAG	CATGCCATGGGTTTTCAATATAATCACCCT	CCGCTCGAGTAGCCATTTTTTCATTGTTC	CCGGAATTCATAGATGTGATGGGTGCTG	TATTGGGATAATTTTGACCGTAAT	AAACATTACGGTCAAAATTATCCC
PY17X_1441100	vacuolar iron transporter, putative	981 / 981	CCCAAGCTTTATGGGGTTTTGTCGTG	CATGCCATGGATTATTGCCAGTCAGGTTA	CCGCTCGAGGCCGAACTAAATGAAAAC	CCGGAATTCTTTACAACATCACCATTATT	TATTGCAATTGTTTCCGGTTGTGT	AAACACACAACCGGAAACAATTGC
PY17X_1367300	E1-E2 ATPase, putative	5705 / 1253	CCCAAGCTTTAACGATTAAAGAGATGTAG	CATGCCATGGGCCAATAGACAATAGAAA	CCGCTCGAGAATAATGGAATAAACTCGG	CCGGAATTCATATGTATCTTCTAATCTTGGA	TATTGTCGTATGGAAATAGCCTTT	AAACAAAGGCTATTTCCATACGAC
PY17X_0109300	zinc transporter ZIP1, putative	2055 / 1462	CCCAAGCTTAAAAATAAAAGGCTGTGAT	CATGCCATGGCGAGTCGTGTAAATAAATAA	CCGCTCGAGTATTTTGCCCCCTTTTTG	CCGGAATTCTGTGAATGCTGATGTGGAG	TATTGTGTGCAATACCTTATATTT	AAACAAATATAAGGTATTGCACAC
PY17X_1424200	cation diffusion facilitator family protein, putative	1557 / 452	CCCAAGCTTAAATTACTACCACAGCGTAC	CATGCCATGGTCGGAGGTTATATTTTTATG	CCGCTCGAGATGTTTGTTACTGCTTTTGT	CCGGAATTCAAGAGGCTATCATTACTCCT	TATTGACAGACCTCTAGTTGGAAA	AAACTTTCCAACTAGAGGTCTGTC
PY17X_1138200	guanylyl cyclase beta	11179 / 864	CGGGGTACCCATTTAATACACACACTTGTATGT	CATGCCATGGACCTCGCTCTTTATTTTATCTG	CCGCTCGAGTGATTCGTTTAAAATCGATGGAT	CCGGAATTCATGCAATAATAATAGTTCAATCA	TATTGTAGCAATTAGATGGGAAAA	AAACTTTTCCCATCTAATTGCTAC
PY17X_0619700	LEM3/CDC50 family protein	1397 / 1397	CGGGGTACCTACGAAATAAATACATGCTAAT	CATGCCATGGACTATGTACATTTTTTTATGACCCA	CATGCCATGGGCTCCAAAAAAGGGGGGAAAAG	CCGGAATTCCGAATTTTTATTTATTTTAAAATATG	TATTGGAATTTTATATTTAATAAT	AAACATTATTAAATATAAAATTCC
PY17X_0916600	LEM3/CDC50 family protein, putative	1122 / 1122	CGGGGTACCAGATCGAACAAGTTGAGCATT	CATGCCATGGATATATTATTAACATTTATCAGAAC	CATGCCATGGGACGAATAAATTAACATAATATA	CCGCTCGAGTCACTATGCAAATTGTACACTC	TATTGGTAATGGGCTTGGAAATGG	AAACCCATTTCCAAGCCCATTACC
PY17X_0809500	P-type ATPase, putative	5295 / 962	CGGGGTACCACACATATCGTTATCCCAATTA	CATGCCATGGTCACATGAAAGCGAAAAAGGA	CCGCTCGAGGGTAGAAAATTTTTGCACACAAT	CCGGAATTCTATGTGTTTCTATACACATGTG	TATTAATTAGAGATTAAATAAATC	AAACGATTTATTTAATCTCTAATT
PY17X_1437200	aminophospholipid-transporting P-ATPase, putative	4838 / 1011	CCGGAATTCATCCAATTATTTAAAAAATCTTGA	CCGCTCGAGCCTTTATGTGTTGCGGCATT	CATGCCATGGATCTGAAGGATCAAGCCATAGA	CGGGGTACCCATAGTAACAATTAAACAAATAGG	TATTGAAACACAAATACGCATCAA	AAACTTGATGCGTATTTGTGTTTC

PY17X_1440800	phospholipid-transporting ATPase, putative	5978 / 1030	CGGGGTACCACATTATTTAGATAATTCTGTAGG	CATGCCATGGCTTCAAATCCGTTAAAATTTACA	CCGCTCGAGTGATGGAGAAACTGATTGGA	CCGGAATTCGTTTCCATTTCCATCTCCTA	TATTGCTTTACGTTAAGCAAAAAA	AAACTTTTTTGCTTAACGTAAAGC
PY17X_0911700	guanylyl cyclase, putative	11553 / 994	CGGGGTACCCTTTCTGCATTCTTAGAATTAAC	CATGCCATGGGTTATTTTTATTGTTTCTTATTTGC	CCGCTCGAGCTGGATATGTGAATAAAGAATT	CCGGAATTCACTTTACAAGTTTATCAAATGTGC	TATTGTCGAAACATCGCTAGTAAA	AAACTTTACTAGCGATGTTTCGAC
PY17X_1105200	MOLO1 domain-containing protein, putative	1594 / 948	CCCAAGCTTGTTGGTATATATCTTTCCCA	CATGCCATGGTGAAGGGCTTTTAAATGGTT	CCGCTCGAGGCTGTAGCTATAGTACCAGA	CCCCTTAAGCGTGTTGGTCAGTTCTGTAA	TATTGCATTCTTATTTTGTTTGCT	AAACGCATTCTTATTTTGTTTGCT
PY17X_1315200	conserved protein, unknown function	765 / 765	CGGGGTACCCGTGAAGTTCAATTTATCCC	CATGCCATGGAGTGCCTTATCCAGAAAGTC	CCGCTCGAGGAAACCAAACAAGAGACGAA	CCGGAATTCCTCAAAGGACATATACTTAC	TATTGATTGTGGAAATTCATCTAC	AAACGTAGATGAATTTCCACAATC
PY17X_1339400	transmembrane protein 43, putative	1869 / 1412	CCCAAGCTTTTTAAATGTCTTATGCTCTG	CATGCCATGGTATCAAACGCTAAACCATAT	CCGCTCGAGCAGAAAGCAAGATTACTAAA	CCGGAATTCCCATTATCTACTTCGTTATT	TATTGTCTATAGTAGATCTAAATG	AAACACCCACTGATACATTTCAAC
PY17X_1342800	conserved protein, unknown function	2358 / 1213	CGGGGTACCCGTTATGCCGAAGAAATTTT	CATGCCATGGGTAATAATGTTCCTTCACAA	CCGCTCGAGGTAATATTGCATGAACACGT	CCGGAATTCCAACGTGTGTTGTAACTAAA	TATTGGTAGAAAGACACCTAAGAG	AAACCTCTTAGGTGTCTTTCTACC
PY17X_1366100	conserved protein, unknown function	2603 / 1347	CCCAAGCTTCTATTCAGTGTTTATATTAAGC	CATGCCATGGGGATAACAAAAAGAGAAGTA	CCGCTCGAGTAAGGACAGCCATATTAAAG	CCGGAATTCCAATTTGGAACTCGGTTACA	TATTGGAGCAAATTGGATTTGCTC	AAACGAGCAAATCCAATTTGCTCC
PY17X_1463300	dipeptidyl aminopeptidase 2, putative	2800 / 1367	CCCAAGCTTGTATTTTTGGTTGTAGAGTG	CATGCCATGGAATATAATGCCACTGTTCAG	CCGCTCGAGCAGAGTGCTATAAAACAGAT	CCGGAATTCTGTAGCTTATCTTTGTCGAT	TATTGATACTTCGGAACATTGTGG	AAACCCACAATGTTCCGAAGTATC
**Primers for PCR-genotyping parasite with candidate genes (except gep1) knockout**								
**Gene name**	**Gene ID**	**P1**	**P2**	**P3**	**P4**	**P5**	**P6**	
PY17X_1243400	7-helix-1 protein, putative	ATAACATTTAAACAGATAGGCG	CAGGTCTATCTGGAATGTTA	GAGAATAAAAGGTCATTGTGGC	CTATTAAACCTGCATAACCTTC	GAGAAACATGCAAATCAATCAA	CTATTAAACCTGCATAACCTTC	
PY17X_1431500	integral membrane protein GPR180, putative	TACAAATAACGAAGGTATAGCA	CTATGTATTTATGCTTACGTGG	TACATTAATGCTTGATATTG	CACAATTTCATATTACGCAA	TACAAATAACGAAGGTATAGCA	AACATACAACCATTACGTGCTT	
PY17X_0918700	serpentine receptor, putative	ATATGCCAAATAAACATGCCAA	ATAAAGACAGACGATAT	TATGAACATATAAGAACGCCAA	CTCTCGTTAATAATTACGTAGG	GTGTGTTACCAAATTGATAGCA	CTCTCGTTAATAATTACGTAGG	
PY17X_1433900	serpentine receptor, putative	TCATTATGATTATTCCCCAGCG	TCCTCCAAATCTGAGTGTTCTG	TTCATGTATTAACAATGGA	TGAAATATCTTCAATTGAAT	TCATTATGATTATTCCCCAGCG	CTCCTAACAAAATATATGGCGC	
PY17X_0524600	serpentine receptor, putative	GATGAGGTGTAAAGACTTCGGT	CTTCATACCTTGCTCTTCACAT	TATATTGTTATGTGTGTACA	ATTATTACACTGAAGCGTTC	GATGAGGTGTAAAGACTTCGGT	CACTTGAAAGAATAACGGATCC	
PY17X_1421700	GPCR-like receptor SR25, putative	TGAAGTGAAGTAGTTAGTTTTACT	ATGCATAAACAGAAAAATAGAATATAAC	CGAGTTTGGTAATAATGAAAGAGAT	ATGTGCACATATATATATAGTGC	TGAAGTGAAGTAGTTAGTTTTACT	AGCAAATTCTTGAATTACTAATGC	
PY17X_0605800	sexual stage-specific protein G37, putative	ATCAAGATAGATATGGTGGTCC	CTCTATACCTACTGATACATGC	TTAGTAACACCTCAACTTATA	TTTAACCTGCTACTACCAGTGG	ATCAAGATAGATATGGTGGTCC	TGCTATGGACAATTCGGGACAA	
PY17X_0617400	conserved Plasmodium membrane protein, unknown function	ATGTATGTTCAGTATACATACA	AAAGTTGATACTGGTATCTA	GTTACCATAGGTCAAGCTCTTA	GCCATACAAAATGCAAAACCCA	GCTAGCCAATAACTAAGACGAA	GCCATACAAAATGCAAAACCCA	
PY17X_0914700	conserved Plasmodium membrane protein, unknown function	GTTATTTGGAGTTACATAAATGT	ATCTGTGCCCCAAACTTCTAAAAAG	GTTATGCGCAATTAAAATCAAACT	AGGGCACCAATGAAAAGAGAA	GTTATTTGGAGTTACATAAATGT	ATTGCCATGGACTTATTTGTTTGCATC	
PY17X_1128600	protease, putative	TTGGATTCCTCACATTAAACTA	GAATCTATAATTGAAAACGCAG	TCCTTCGCTATATGTGCGAT	AATATAACCCTGCTGAAGAGCA	TTGGATTCCTCACATTAAACTA	GTAATATCATGAATGCACACCG	
PY17X_1313900	conserved Plasmodium protein, unknown function	TTTATTCGATGGCATTTCAT	CCATTTGTTACTATATATGG	AAGATTGTTGTTGTTTGGGCTT	TTGTTTGCTATGTTTTATGTCC	ATGATACCCAAGAAATTGAGAG	TTGTTTGCTATGTTTTATGTCC	
PY17X_0702400	folate transporter 1, putative	AAAACTGTTAATGCTCTT	ACTAGATGGTGTTGACAT	TTTACATTTCCACCCGTT	TCATGTCCATTTTCTGTG	TTTACATTTCCACCCGTT	GCAAAGAACCTCAACTCC	
PY17X_0933500	folate transporter 2, putative	AAGGAAAAATCAAAGAAT	CGTGCCTTAGTACGTGAT	ATACATGAGTCTGCACCCTA	GATTCTCATCCATTAGGTTC	ATACATGAGTCTGCACCCTA	GTATTTCTGTAATGGCTTCA	
PY17X_0309400	GDP-fructose; GMP antiporter, putative	ATAATGCCGAAAGGTTAA	AAATCCTGATCTCATCAT	GTGCAAACTTAACTAAAATACCCCG	ATCATACTTTTTCACACAAACAAACTG	ATAATGCCGAAAGGTTAA	CATCCTCCCTTTCTCTCT	
PY17X_0936300	UDP-galactose transporter, putative	CATATCCATGTTTAGGTT	TCACCATTGAAACTAGAA	CAGCAAGTTTAGTAGAGCAAGT	ATTGACAATGATGCAAATATCCAAGC	CATATCCATGTTTAGGTT	TTATCTTGTCTTGGTCCT	
PY17X_1436400	phosphate translocator, putative	CAAACCCAAAGGAAGAAC	ATTGATACGAGCAGAGAG	TAGATCTATATACACATCCCTGTGAT	ATCACAGCACCGAGAGATACAAAGCC	CAAACCCAAAGGAAGAAC	GCTTCCTCGTTAGGCTTG	
PY17X_0823700	major facilitator superfamily domain-containing protein, putative	TATTTGTGTGTGCAGTCT	AAAAATGGGCAAATCTACAC	TTTATACAAAATGATGTACCTTAC	ATTGAATAGAAGAAGTTACACTATG	TATTTGTGTGTGCAGTCT	TAAAGGGGTATCCATAGT	
PY17X_0820300	major facilitator superfamily domain-containing protein, putative	GGGTGTATGTATATATGTGTAAGTACTT	ACCGTAAATGTATGCATGGCTCAC	TAAATAGTATGAGGGCAAA	TGGTAAATAAACAACAGGT	AGTGCAAACAGTACGAACT	TGGTAAATAAACAACAGGT	
PY17X_0307300	transporter, putative	TTTCCGTATGTTTAGTATCT	CTTGTTCATAACTTCTTGTA	TTTTGTATTATTGTTAGA	GATATTTGTTCAGGTGTA	TTTCCGTATGTTTAGTATCT	TCTGTTTTCTCAAATCTGTA	
PY17X_0917400	amino acid transporter	AGAAAAAAGTAAGTGGTGTG	GTTTTCTATCTGTTTGCTCA	CCAAATATAAGAAAGAGGGGAGGTGT	ATTCTATCTAATATTGGCTCCC	AGAAAAAAGTAAGTGGTGTG	ATGTTCAGAATTTATCCTTA	
PY17X_0609400	amino acid transporter, putative	CCTCGTGTGTACACATGGCACCT	ATTTGGTATCATTGTCATAGATCG	TTGTGTTCATTTACACCCGT	TCAATATGAGGGTAAGCGGT	TTGTGTTCATTTACACCCGT	CCCTTATGCCTATCACTTTT	
PY17X_1448600	amino acid transporter, putative	TCGTTTTATCGAGCATGT	GCTCAGTTGCTGCTTTTT	TTGCTGCAATTCTATACATA	TCTTAGAACAACTTAACGCT	TTGCTGCAATTCTATACATA	TCAAATAAGCCTACAGACAA	
PY17X_1241000	multidrug resistance protein 1, putative	GTGATAATAATAAGAATAATA	CACTAGTTGTATTTCTCTTA	GAACAAGTAAATGCAGGAATAG	TATCCATATTTGTATCAGTAAC	GTTGATAGAAATCAAAATAA	TATCCATATTTGTATCAGTAAC	
PY17X_1446300	multidrug resistance-associated protein 2, putative	CTATGTATGCAAACAATTTGACAATGTT	ATTGTCGTGATATGGGCTTAA	GTAATTCATGGAATTCATG	CCGAATTAACATCAATATGATC	GGACTGATTTCGGCATTGGCCTAT	CCGAATTAACATCAATATGATC	
PY17X_1315500	multidrug resistance protein 2, putative	CTTATAGCGACATTTTTTGGATGTT	ACATGTGAATATAATACACCCG	GCTGTGTTAATAACATATAACCT	CAATAGTAGAATATAATGTTCC	GATTTAGGTAAAAAATGGGA	CAATAGTAGAATATAATGTTCC	
PY17X_0904900	ABC transporter B family member 3, putative	GTGTGTAAAACACTTAAGGA	CACCACTATGTATAGACATG	GAATTATAAGTTTAGGGTTGTGTCT	AAAGCMAAATAAAGGGCTAG	GTGTGTAAAACACTTAAGGA	CAATTTTGATCAATAATATC	
PY17X_0403400	ABC transporter B family member 4, putative	GAACAACAATATTCACGGATTCAAGAAT	ATTGTCTTCATTAGTGGATATGTCAC	CTGGACATACAGATAACAACAC	CTATCTAAACAGACAATTTTGG	TATACATGGTACTTCTAGTC	CTATCTAAACAGACAATTTTGG	
PY17X_1358700	ABC transporter B family member 5, putative	CGAATAACACATGGACAGCTTGTATCT	ACCCATAGAATGAAAAGGTAGATG	TCAGGAAAATCGACCATATGG	GTCCAGGCTTATCTTGTTCGCC	GATTAGACGATGAACTAAAGG	GTCCAGGCTTATCTTGTTCGCC	
PY17X_0928600	protein GCN20, putative	GATATAGTGACAAAATGGCTGTTGTATCAT	ATTCTCATTCCTCCACTTAGAGAGTTTAC	TGGCCTTTCACATAGCTTA	CTCGAGTCCATATCTACTTC	GTTAAGTTTATTTAAAGAAT	CTCGAGTCCATATCTACTTC	
PY17X_1370600	ABC transporter B family member 6, putative	TATGGCTGCATAAAAATTGG	GTTATTATACAATTGAATAT	TTCGGGGATTGCTTTATTCAAAGT	GTATTTTGTTCTGACTTGTTCAT	AGTATACAATCGACATATCC	GTATTTTGTTCTGACTTGTTCAT	
PY17X_0610800	ABC transporter B family member 7, putative	GGTACTATATATTCCTATGTAGT	AGTGTTTCTTCTTTTCCTCCATAC	GTGATGAAGTACAGTTAGAAG	TCCTACTATAGCAACAGATT	GCAGATTCGAGTTTCGCATG	TCCTACTATAGCAACAGATT	
PY17X_1145400	ABC transporter E family member 1, putative	GTAGTCTTGATTTATGGCA	GGTAAAAGAGTTCAATGTTT	CTGCGATACAGTTATATGATA	CATACCTGCTACTAATGTTTGTG	GAATGGCGAGATATATTATC	CATACCTGCTACTAATGTTTGTG	
PY17X_1019600	ABC transporter G family member 2, putative	GTATATATGCATATATAAAAGG	ATTTCACTATCATGATCATTATGAATTATTG	ATGCATCATACCAATCACCTA	GAATGATCCATATAATGCTA	GCATTTATAAGAGGTATAAG	GAATGATCCATATAATGCTA	
PY17X_1425800	ABC transporter F family member 1, putative	CATATATAAATACAAACATAT	CAACTTTTCAAAATCAGAAA	GGCGCAATAGACGTTAACT	ATTCTTCTTCCTCATCAGAA	GATGAGAATGATGATGAGAATG	ATTCTTCTTCCTCATCAGAA	
PY17X_1222000	ABC transporter I family member 1, putative	GATAATATTGATTTTTGTTCTC	CATTTGCACTGAGATTGTCGA	ACTCACATATATACAGATATG	CTGTAACTATGCTACTATCTG	GCATTATGTCGAATAGCATA	CTGTAACTATGCTACTATCTG	
PY17X_1031600	FeS assembly ATPase SufC, putative	GAGTAAAACGACACAAATGTGAAAT	ATTGTGAACCCTCATATATTGTACATGTTC	GGCTAGCTGTGTCATACTAT	CGCATATTTCTTCGCAATGCTC	GTTAATCATAGATCATTATG	CGCATATTTCTTCGCAATGCTC	
PY17X_0407300	ER membrane protein complex subunit 5, putative	CCTAACAATGTTGCACACAGTAATACT	AGCATTAACAAACTTGTTCCGATTGATG	TAGTATGCTTCTTTTTGCG	ATAGGTCTGTCCTCTTTGC	ATGACTCAACTTTTGCTCTA	ATAGGTCTGTCCTCTTTGC	
PY17X_0929900	CorA-like Mg2+ transporter protein, putative	GTCTGAAGCGCTTCCCCCCCTCAACT	ATCTTTTCGTATTAAAATTGTTGGGTTGTGG	TTTGAGTTTGTTGATATTCG	GGACCATACAGAAACAAAA	GTAGCTGATGATGGAAATG	GGACCATACAGAAACAAAA	
PY17X_1018500	CorA-like Mg2+ transporter protein, putative	GCTAGCTATATATGTAAATATGTTTT	GTAAAAGATAATGAACATTTTCTCCGTTAC	AAACTAAACCATTGTATTCG	GCTAATGTCCAAAAAATGTA	TGTAATGAAAAAGAACCCA	GCTAATGTCCAAAAAATGTA	
PY17X_0703300	magnesium transporter, putative	GCTAAATGCTGTAACATATCGAAT	ATCTCTCCTTTTTATCATCTTCTTTTGTTTCGTC	ACGCTGCTCTATACATCACT	AAATAGAATACTGCGAAGAT	AGTTGTTGCTCCGTTTGT	AAATAGAATACTGCGAAGAT	
PY17X_1240600	inner membrane complex protein, putative	CATTGAAAATTTGTAAAGTTGAAGCATTTG	ATGCACATGCTGCAATAGTAGCATATGTGAAC	AGGGTGATTATATTGAAAAC	TTTTCTTACACTTGAACACA	CTCATATAGGAGATCACCAT	TTTTCTTACACTTGAACACA	
PY17X_1441100	vacuolar iron transporter, putative	GAAGAAGTGGCATTACTAATTAACAAT	AGGTTGGTAACTAAAATGTTAAAAACGCA	TAACCTGACTGGCAATAAT	GTAAGACGATAAAATGTGAA	GTTAGCAGAAAGACAAAGAG	GTAAGACGATAAAATGTGAA	
PY17X_1367300	E1-E2 ATPase, putative	GTATGAATAAAATTGACAAATTTGTATACAT	ACCTGAATTGGTATTACCCGAGTTTATTCC	TTTTTCTATTGTCTATTGG	CATGTTTCTCTATTATTTTC	CTATTTCCATACGACTCTA	CATGTTTCTCTATTATTTTC	

PY17X_0109300	zinc transporter ZIP1, putative	GTTGATGTCGTTCTATATTCATT	ATCTTGAGATTTTGGCTCTGAAAAAGAGTGCAC	GTAATAAATAACTACGGTCA	GATAATAATGTGTCTTCCCT	TTTCTAAAGTCTATCAACGC	GATAATAATGTGTCTTCCCT	
PY17X_1424200	cation diffusion facilitator family protein, putative	CTTATTTTCATTGATAAAACGCTGCCT	ACAAAAGCGATAAATATATTTGCTAGTG	GAGCGTCACAGCTACATGACCAT	ATTGGATCTGTGATTGAATATTTAGGGTTATACC	CAAGTTGAGCAAGTTGATGGAT	ATTGGATCTGTGATTGAATATTTAGGGTTATACC	
PY17X_1138200	guanylyl cyclase beta	GTCTACACCTGACTGGACATA	ATGCAATAATAATAGTTCAATCA	ATGGAGGCTTAATATTGGGT	TAATTCTTAATGTATATAAAAGTATAGACA	GTCTACACCTGACTGGACATA	TGCGTCGAGTGATATCAAC	
PY17X_0619700	LEM3/CDC50 family protein	GGTCATTCTAATGTTATAAGA	GAAATGTTATTGCATATCCACC	CAAGACGATTCCTCTATATGTATGC	TCTACATAATAAAAGCATCGC	CACATTGTGTCTTATTTACAACC	TCTACATAATAAAAGCATCGC	
PY17X_0916600	LEM3/CDC50 family protein, putative	AATTTCCCTTTGGGGTTTCAC	ACATGTTAATATTTATCCGAATGGA	TGTGTGATTATACAATTGCTTATGA	GGAAATATATTACAAAACATAGTG	GGAAATATATTACAAAACATAGTG	TGGATGATACCATCACCATTTAG	
PY17X_0809500	P-type ATPase, putative	GGAAACGTGCATATATCCACA	TGTGTTTACAAGTGATGTACCTCT	ACTTAATGCATACCTTCCTG	ATACATATATATACGCGTATATTGTGT	GGAAACGTGCATATATCCACA	TAGTTGTAACGCCGATATCGA	
PY17X_1437200	aminophospholipid-transporting P-ATPase, putative	TCCTCCACCTTATAAACCATAT	TAGGTGGTAATAATATCACTG	TGAGTATAAAGCATACTCACAAAG	GGTAACTTAACATATTTATCATCAT	CATGAATATGTGTAAAAAGGACGA	TGAGTATAAAGCATACTCACAAAG	
PY17X_1440800	phospholipid-transporting ATPase, putative	TCATCGAAGAAACAAATGAGTA	ACCATATTGATTGTATTTAAACAT	CACTGTCAACAAAATTTATCATAC	GAAGAGATTGTAAATTTTAA	TCATCGAAGAAACAAATGAGTAA	TCTTCATCTTTACTTAAATTTCGT	
PY17X_0911700	guanylyl cyclase, putative	ACACACCCAGCACACATATTTAG	ATGTATTTAAATAAACCATTTGTAGC	ATATTGTTTGATGTTGGTTTTG	CTTGCTTACCTCTATTACTGCA	ACACACCCAGCACACATATTTAG	TCCCATAATGGTTGATTATTTG	
PY17X_1105200	MOLO1 domain-containing protein, putative	GTTATATCTACACATATGCATG	CGTGTTGGTCAGTTCTGTAA	ATGTTGCTTTATCGTCTT	ACAGCCAAACAGTAGTCA	GCATTCTTATTTTGTTTGCT	CGTGTTGGTCAGTTCTGTAA	
PY17X_1315200	conserved protein, unknown function	ACTAACTCGCTATTTAGCCT	CTCAAAGGACATATACTTAC	TATACGCATGTCAATCTT	TTCAGTGCAAATTATGTT	GATTGTGGAAATTCATCTAC	CTCAAAGGACATATACTTAC	
PY17X_1339400	transmembrane protein 43, putative	CATACACTATATGCATATATATAAGC	CATCTTTATTCATGATATTAACTG	AATATTTGTGGGGGTGGTGTTTA	CGTAGAAGATAATTGTACAG	GCAACATCGCTTACTATAATCC	CATCTTTATTCATGATATTAACTG	
PY17X_1342800	conserved protein, unknown function	TGCCATTTTCCCCCACCCTTTTTGTTAGG	TAAACATAATGGGAATAACCATATATGGG	GACGGTTTACAACTTATT	GTAATGATGGCTGTTCTA	GGTAGAAAGACACCTAAGAG	ACTTCGGCCATCAAATTATC	
PY17X_1366100	conserved protein, unknown function	GTGTAACATAAAAGGAAAAT	CTAAATAAACAAATGGACGT	GGTTTAAAATACCTTGAT	CTAACTCGATTTATAGGG	GGAATATTTACAATATTTTA	CTTTAATATGGCTGTCCTTA	
PY17X_1463300	dipeptidyl aminopeptidase 2, putative	TAGTTTGGTACTATTTAGCG	CATAAAATATCCATCCTAATC	ACACCCATTTTGCCTGAA	TCAACTGTTTCGCAAGGA	TACAAAACCAATGTCTAACC	AGAGATAGGCATCTTTGTAA	

Oligo sequence for gep1 (PY17X_1116300) knockout plasmid construction

Gene ID	KO	Gene size (bp)/ deleted gene size (bp)	Left homologous arm		Right homologous arm		Target site of sgRNA	
			Forward primer	Reverse primer	Forward primer	Reverse primer	Oligo (Forward)	Oligo (Reverse)
PY17X_1116300	gep1 N-terminal KO	3333 / 464	CCCAAGCTTTTGGCTAAGCAAATGTAT	CATGCCATGGCCAAAACGAAATTAAATC	CCGCTCGAGAAACAAGATAGAGTAGAA	CCGGAATTCAGATGAACTTAATGTTGT	TATTGTGTTAGTAACAATAAATAAA	AAACTTTATTTATTGTTACTAACAC
PY17X_1116300	gep1 C-terminal KO	3333 / 558	CGGGGTACCCCTTTATGCTTATATCAGCA	CATGCCATGGTGTGCAAAATGTACACATGC	CCGCTCGAGGGGCTATTACTATTATTGCA	CCCCTTAAGGTAGTGTTGTCCAATTTGAA	TATTGTTAAACCAGTCTATATATT	AAACAATATATAGACTGGTTTAAC
PY17X_1116300	gep1 full length KO	3333 / 3333	CGGGGTACCCACAACTCTACAAATAAACA	CATGCCATGGCTTTTTTTCAAACTTACAAA	CCGCTCGAGACAAACATTTTTCATATTTT	CCCCTTAAGGTAGTGTTGTCCAATTTGAA		
PY17X_1116300	gep1 full length replaced with mScarlet							

Primers for PCR-genotyping parasite with gep1 (PY17X_1116300) knockout

Gene ID	KO	P1	P2	P3	P4	P5	P6	P7
PY17X_1116300	gep1 N-terminal KO	ACGCACATTGGCCATAAACATG	CAAAGACACTTGCCATTTCAAT	GCTTGGCTAAGCAAATGTATCA	TCTTGCAATAAAAATGGAGCCG	TATTGACATTAGGATTTCGAAGTGT	TCTTGCAATAAAAATGGAGCCG	/
PY17X_1116300	gep1 C-terminal KO	ATATACTGCACATACCTGATTG	GGCACATACACATACACATATA	ATATTCCGTCTTTCAATGGA	CTTAAGAGTACGCGATATATAGACAAGA	ATATACTGCACATACCTGATTG	GAATTCGAGTTGGATATGCTTT	
PY17X_1116300	gep1 full length KO	ACGCACATTGGCCATAAACATG	CCCCTTAAGGTAGTGTTGTCCAATTTGAA	CGGGGTACCCACAACTCTACAAATAAACA	CTTAAGAGTACGCGATATATAGACAAGA	ACGCACATTGGCCATAAACATG	TCTTGCAATAAAAATGGAGCCG	
PY17X_1116300	gep1 full length replaced with mScarlet							ACGCACATTGGCCATAAACATG/CCGCTCGAGCTACTTGTACAGCTCGTCCA

Oligo sequence for gene knockout plasmid construction

Gene ID	Gene name	Gene size (bp)/ deleted gene size (bp)	Left homologous arm		Right homologous arm		Target site of sgRNA	
			Forward primer	Reverse primer	Forward primer	Reverse primer	Oligo (Forward)	Oligo (Reverse)
PY17X_0619400	nek4	1709/1709	CCCAAGCTTGTAAGCAAAGGTTAATACAC	CATGCCATGGTTGTGAGGACGTGTATAATA	CCGCTCGAGGAGTATAATATTACAGTCCA	CCGGAATTCGAAATGGTACACCCTGTTCT	TATTGAACAAGTATGAAAAAATAA	AAACTTATTTTTTCATACTTGTTC
PY17X_0935700	map2	1587/1587	CCCAAGCTTAGTCTCCCAATTTTTCTGTG	CATGCCATGGAAGGAGAATGTGCGTATCTA	CCGCTCGAGTATGTTTCGTCGAGAAAGGT	CCGGAATTCTTGGGAATATGAGCATTCGT	TATTGAAAAATTTATTCACGAATC	AAACGATTCGTGAATAAATTTTTC
PY17X_0617900	cdpk4	1792/465	CGGGGTACCGAAGTAGATGCAGCTAGAATA	CATGCCATGGGTTCTAATGCATCTCTTGCTG	CCGCTCGAGCTGCAAATGAATTAGCTCAAT	CCCCTTAAGTGTGTATATTCCACGATATTT	TATTGACAGAAATGAATTAATAAT	AAACATTATTAATTCATTTCTGTC

Primers for PCR-genotyping parasite with gene knockout

Gene ID	Gene name	P1	P2	P3	P4	P5	P6	
PY17X_0619400	nek4	CTTTTGAAAACCGATAAAAAGTG	CCGGAATTCGAAATGGTACACCCTGTTCT	CCCAAGCTTGTAAGCAAAGGTTAATACAC	TCACACCGTATCATATTGTGTC	GAGTAAGATTGTGTGATTTTGG	TCACACCGTATCATATTGTGTC	
PY17X_0935700	map2	GGGAAATACTGATAATAGCGACAA	CCGGAATTCTTGGGAATATGAGCATTCGT	CCCAAGCTTAGTCTCCCAATTTTTCTGTG	AGACACACTCACATTACGATTG	GATATATGGTCTACTGGTTGTA	AGACACACTCACATTACGATTG	
PY17X_0617900	cdpk4	GCTCTTCCTTTGGACATAGTTA	GGGCTGAAAACCAAATAAGTAT	GAAGTAGATGCAGCTAGAATA	AGACGGATAAAATGTCGGAT	AGTACACAAAAGTTAGCACAAG	CCTTTTCTAATCTCTCAGTTGA	

Oligo sequence for gene knockout in P.berghei ANKA plasmid construction

Gene ID	Gene name	Gene size (bp)/ deleted gene size (bp)	Left homologous arm		Right homologous arm		Target site of sgRNA	
			Forward primer	Reverse primer	Forward primer	Reverse primer	Oligo (Forward)	Oligo (Reverse)
PBANKA_1115100	gep1	3166/785	CCCAAGCTTGCACATTGATCATAAACTTG	CATGCCATGGCTTGTCTGTACATGGACAAA	CCGCTCGAGCGAATTTCATAATTATGATG	CCGGAATTCCATACATCCCTATTGATATA	TATTGTTATAATATAAAATATGAG	AAACCTCATATTTTATATTATAAC
PBANKA_0933700	map2	1572/807	CCCAAGCTTGTCTCCCAATTTTTATGTGT	CATGCCATGGTGATCGTATGGGAAAACGCT	CCGCTCGAGTCACGAATCAGGTATTATCC	CCGGAATTCCATCTGAAATAGATGGGTAT	TATTGCAATGAAGAAGATAAAAGT	AAACACTTTTATCTTCTTCATTGC

Primers for PCR-genotyping parasite with gene knockout in ANKA

Gene ID	Gene name	P1	P2	P3	P4	P5	P6	
PBANKA_1115100	gep1	GCACACACAAACTCTTATGCGCATAC	ACAAATCTCCCTCCATTTATTTCTTG	CGAGATTTGTCCATGTACAG	CTCAATAAGGGAGAAATCACGT	GGAAACGACAAATAAAGAAA	CATTATATGATATACCAATAG	
PBANKA_0933700	map2	GCTATTATGAAAAAAGGAGCTAACAT	TTATGTGGCCCTGGTTCTTCATTTTC	CGATTTTGTCAATCCATATATTT	GCCTGGTCAATTGTGATTCGTT	GATGATAGCACATATATAGAT	GTCCGAATCGGCTATTTCTA	

Oligo sequences for gene tagging plasmid construction

Gene name	Tag	Gene ID	Left homologous arm		Right homologous arm		Target site of sgRNA	
			Forward primer	Rerverse primer	Forward primer	Reverse primer	Oligo (Forward)	Oligo (Reverse)
sep1	C-terminal 4Myc	PY17X_0526200	CCCAAGCTTTTGCCTTTTTATTGGCCATA	CATGCCATGGGTGTGATGCTGGTGGATAA	CCCCTCGAGTTCTAATTTCTGATGGTTAT	CCCCTTAAGCACGGTACTACTTACAATAA	TATTGTATTAGTTTCTGGTGCGGG	AAACCCCGCACCAGAAACTAATAC
gep1	N-terminal 6HA	PY17X_1116300	CATGCCATGGCACAACTCTACAAATAAACA	CCGGATATCCTTTTTTTCAAACTTACAAA	CCGCTCGAGGAAACATCAAATAAAGAAAT	CCCCTTAAGTATACCAATAGATACAAGAT	TATTGAGCTATTTTATGAGAATAA	AAACTTATTCTCATAAAATAGCTC
gep1	N-terminal 4Myc	PY17X_1116300						
gcα	C-terminal 6HA	PY17X_0911700	CGGGGTACCGTGTATACCAAATTTAAATATGAGA	CATGCCATGGGCAAAATTGAACTTGTGTCTTTGGA	CCGCTCGAGTACTTTTAGAACTTTCTAATTTATG	CCGGAATTCCCATGTACTGTGAACATATCA	TATTAATCGTCATATAAAATATTT	AAACAAATATTTTATATGACGATT
gcα	C-terminal 4Myc	PY17X_0911700						
gcα	N-terminal 6HA	PY17X_0911700	CCCAAGCTTCTTTCTGCATTCTTAGAATTAACATGTATTATTGGAT	CATGCCATGGCTTTTATATCTTTATCTGTTATTTTTATTGTTTC	CCGCTCGAGATGCAGACGAAAAAAGGAAATGAAAAAGT	CCGGAATTCAGAAGTTGATCCATCTATGATGTGACATAATC	TATTCAATAGTAATATGAAAAATC	AAACGATTTTTCATATTACTATTG

Primers for PCR-genotyping with gene tagging

Gene name	Gene name	Gene ID	P9	P10	P11	P12		
sep1	C-terminal 4Myc	PY17X_0526200	CATGTGCAAAAATGAAATTAGCAAAAGC	CCCCTTAAGCACGGTACTACTTACAATAA	GCTAGCGCCGGTTCTGCTGCTAGA	GTAATGGTGATTCAGGGT		
gep1	N-terminal 6HA	PY17X_1116300	GCTTGGCTAAGCAAATGTATCA	CCCCTTAAGTATACCAATAGATACAAGAT	CATGCCATGGCACAACTCTACAAATAAACA	CAAAGACACTTGCCATTTCAAT		
gep1	N-terminal 4Myc	PY17X_1116300						
gcα	C-terminal 6HA	PY17X_0911700	TTAGAAATGGCATATTCAATG	CCATGTACTGTGAACATATCA	GTGTATACCAAATTTAAATATGAGA	ATGAAATTAATTTCCAAAATGGA		
gcα	C-terminal 4Myc	PY17X_0911700						
gcα	N-terminal 6HA	PY17X_0911700	CACACACCCAGCACACATATTTAGAT	ATCCAAGCAGCTTCTGCTGTTGC	CCTTGCTTACCTCTATTACTGCACCATTGAT	ATCTGCAGGTACTTGCTCATTTTCTACTAAGCG		

Oligo sequences for gcα promoter swap plasmid construction

Gene name	Tag	Gene ID	Left homologous arm		Right homologous arm		Target site of sgRNA	
			Forward primer	Rerverse primer	Forward primer	Reverse primer	Oligo (Forward)	Oligo (Reverse)
gcα	N-terminal 6HA & promoter swap	PY17X_0911700	CCCAAGCTTCTCAATATGGTTGCATATAT	CATGCCATGGAAGAAGATAATGTGCATACAC	CCGCTCGAGCAGACGAAAAAAGGAAATGA	CCGGAATTCGAAGTTGATCCATCTATGAT	TATTCAATAGTAATATGAAAAATC	AAACGATTTTTCATATTACTATTG
**Primers for PCR-genotyping with gcα promoter swap**								
**Gene name**	**Gene name**	**Gene ID**	**P9**	**P10**	**P11**	**P12**	**P13**	**P14**
gcα	N-terminal 6HA & promoter swap	PY17X_0911700	TCATCCAGGTGTAGACTAAAAT	CCGGAATTCGAAGTTGATCCATCTATGAT	CCCAAGCTTCTCAATATGGTTGCATATAT	AGCGTATAATACTACCTACAGT	AAACACAGACATAACTCCTTTAGA	AGCGTATAATACTACCTACAGT
**Primers for RT-PCR**								
**Gene name**	**Gene ID**	**Forward Primer**	**Reverse Primer**					
18s Rrna	PY17X_0522400	GGTTTTATAATTGGAATGATGGGAAT	ACGCTATTGGAGCTGGAATTACC					
gcα	PY17X_0911700	GTTGTAATCATAGTGATGGTTC	TCACATGTTATTCTTCCTCTAA					
**Peimers for gene in situ complementation**								
**Gene name**	**Tag**	**Gene ID**	**TS**		**Left homologous arm**		**Right homologous arm**	
			**Oligo (Forward)**	**Oligo (Reverse)**	**Forward primer**	**Reverse primer**	**Forward primer**	**Reverse primer**
Pygep1	C-terminal 6HA	PY17X_1116300	TATTGCGGACGCTAATCGTAGCTA	AAACTAGCTACGATTAGCGTCCGC	CGGGGTACCCCTTTATGCTTATATCAGCA	CATGCCATGGACCCCTTATTGAAAATTCAC	CCGCTCGAGACAAACATTTTTCATATTTT	CCCCTTAAGGTAGTGTTGTCCAATTTGAA
