# Supplementary figures and images for: An intracellular membrane protein GEP1 regulates xanthurenic acid induced gametogenesis of malaria parasites
Source: Nat Commun. 2020 Apr 9;11:1764. doi: 10.1038/s41467-020-15479-3 (PMC7145802; doi:10.1038/s41467-020-15479-3)

Fig. 2c

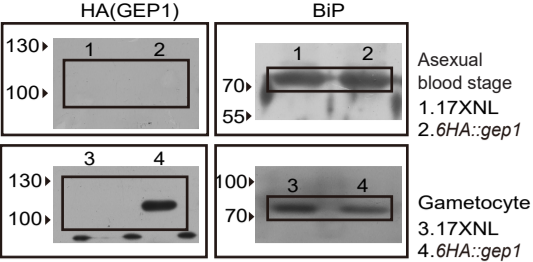

Fig. 2h

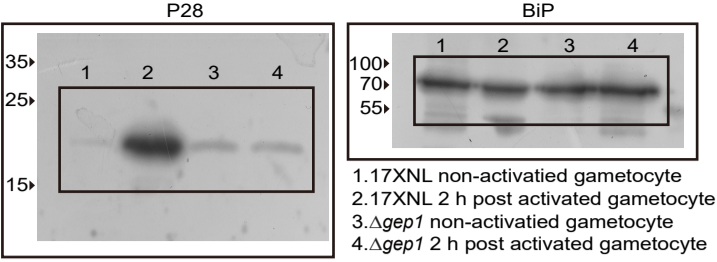

Fig. 5c

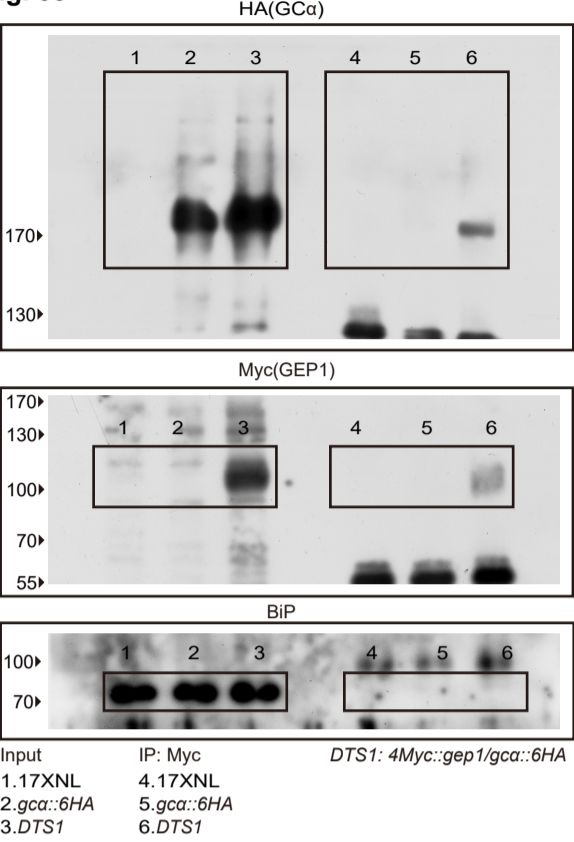

Fig. 5d

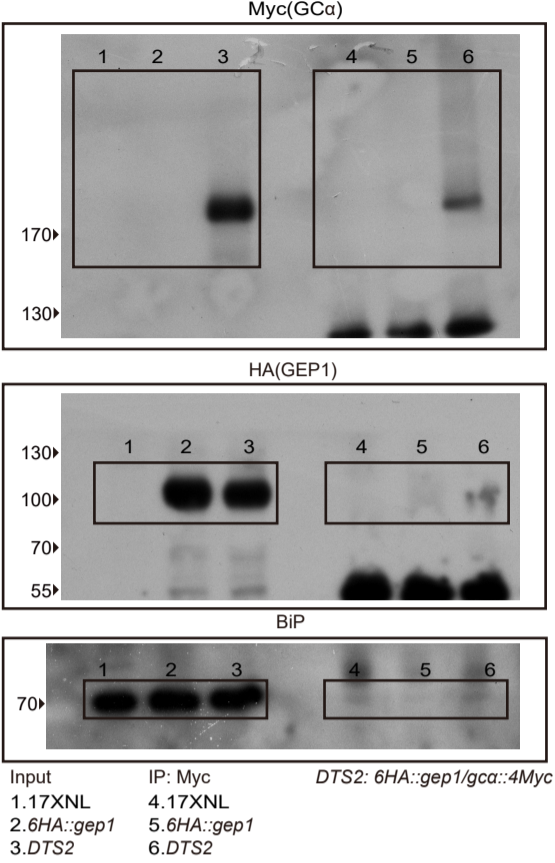

Fig. 6b

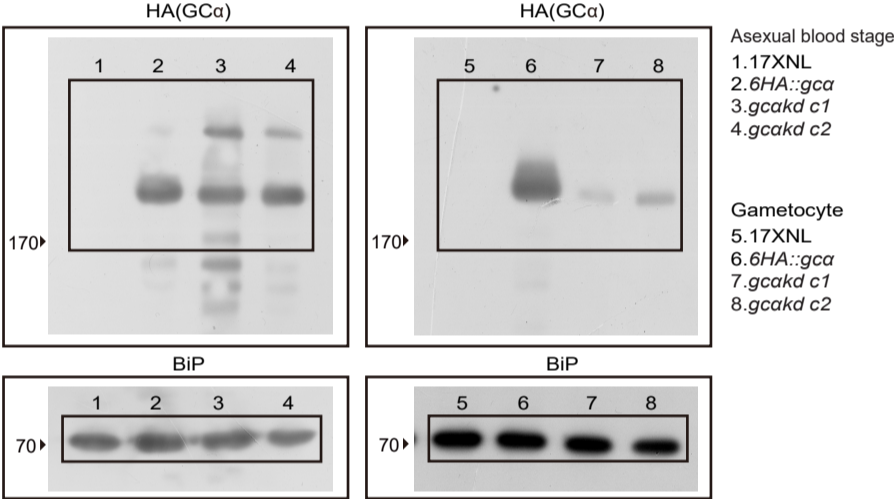

Fig. 7a

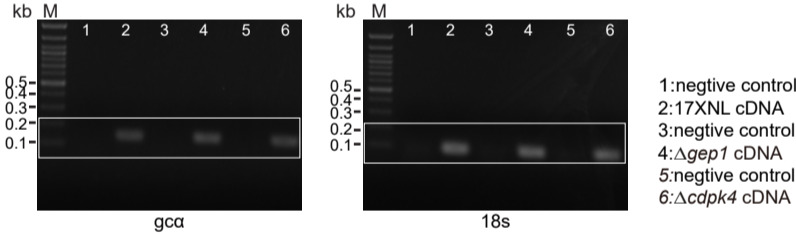

Fig. 7b

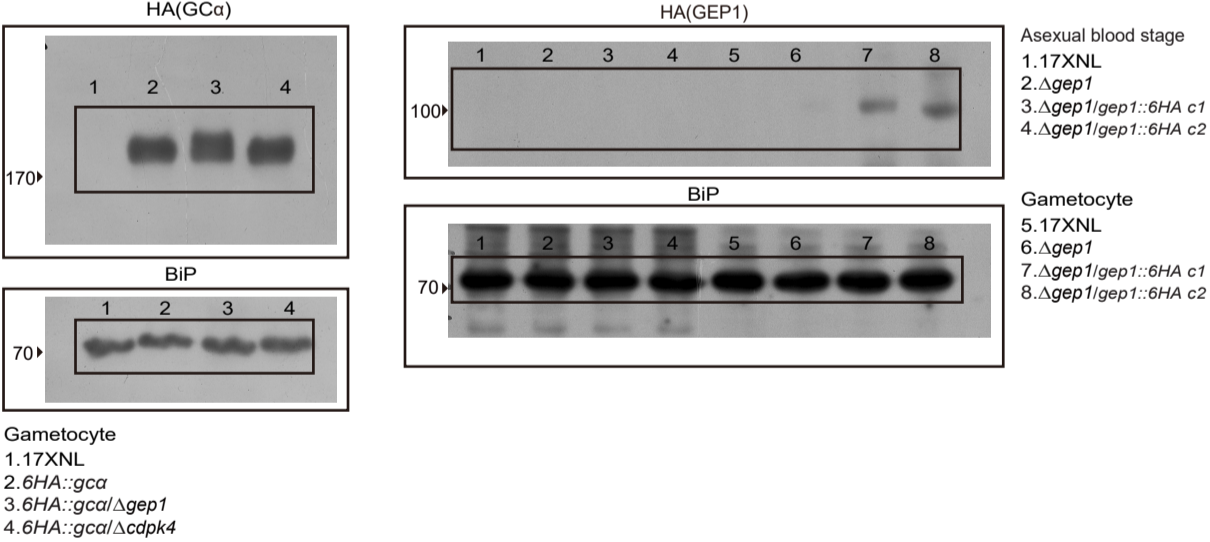

Fig. 7c

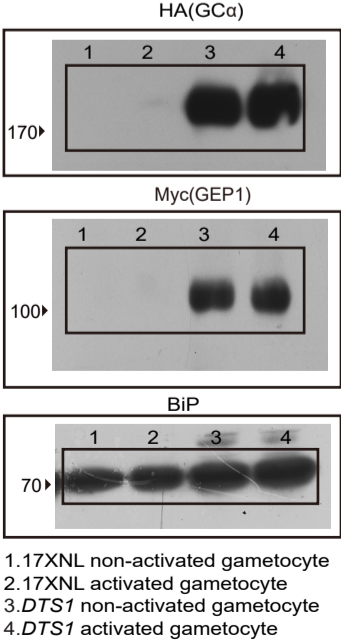

Supplementary Figure. 8b

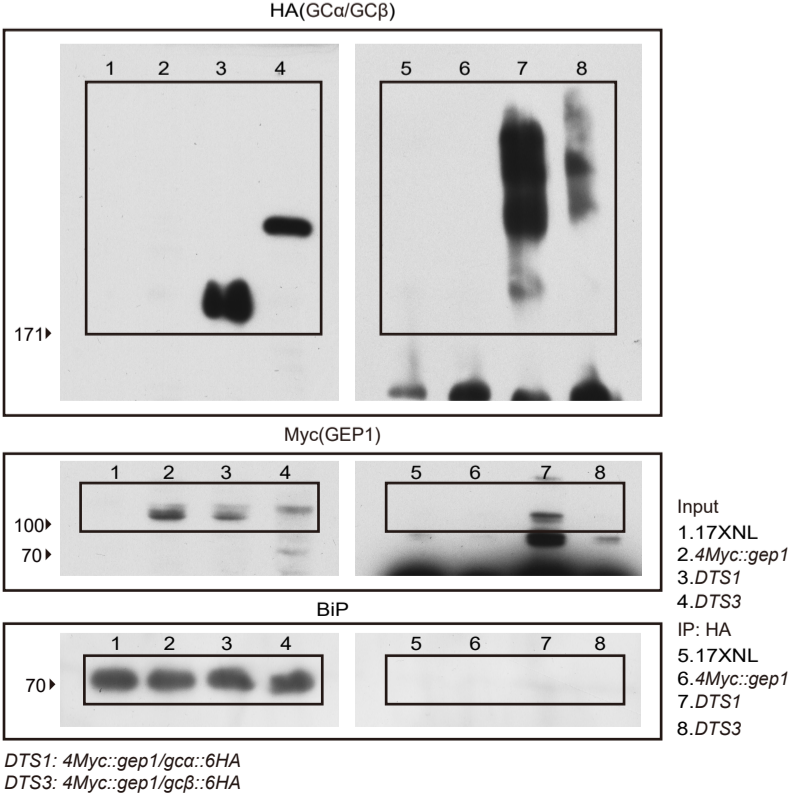

Supplement: Supplementary file 3 — Source Data [file 41467_2020_15479_MOESM3_ESM.zip › Source data/Western and gel scan.pdf]
